# Supervised discovery of interpretable gene programs from single-cell data

**DOI:** 10.1038/s41587-023-01940-3

**Published:** 2023-09-21

**Authors:** Russell Z. Kunes, Thomas Walle, Max Land, Tal Nawy, Dana Pe’er

**Affiliations:** 1 Computational and Systems Biology Program, Sloan Kettering Institute, Memorial Sloan Kettering Cancer Center, New York, NY USA; 2 Department of Statistics, Columbia University, New York, NY USA; 3 Clinical Cooperation Unit Virotherapy, German Cancer Research Center (DKFZ), Heidelberg, Germany; 4 Department of Medical Oncology, National Center for Tumor Diseases, Heidelberg University Hospital, Heidelberg, Germany; 5 German Cancer Consortium (DKTK), Heidelberg, Germany; 6 Howard Hughes Medical Institute, Chevy Chase, MD USA

**Keywords:** Computational models, Genetic databases, Breast cancer, Immunosurveillance, Cancer immunotherapy

## Abstract

Factor analysis decomposes single-cell gene expression data into a minimal set of gene programs that correspond to processes executed by cells in a sample. However, matrix factorization methods are prone to technical artifacts and poor factor interpretability. We address these concerns with Spectra, an algorithm that combines user-provided gene programs with the detection of novel programs that together best explain expression covariation. Spectra incorporates existing gene sets and cell-type labels as prior biological information, explicitly models cell type and represents input gene sets as a gene–gene knowledge graph using a penalty function to guide factorization toward the input graph. We show that Spectra outperforms existing approaches in challenging tumor immune contexts, as it finds factors that change under immune checkpoint therapy, disentangles the highly correlated features of CD8^+^ T cell tumor reactivity and exhaustion, finds a program that explains continuous macrophage state changes under therapy and identifies cell-type-specific immune metabolic programs.

## Abstract (web summary)

Spectra decomposes gene expression data into interpretable programs using prior knowledge.

## Main

A key challenge in the interpretation of single-cell RNA-sequencing (scRNA-seq) data is to retrieve coherent interpretable gene programs representing cellular processes and to quantify them in response to perturbation. Gene programs are sets of genes defined by common tasks, such as metabolic pathways or responses to inflammatory cues. Gene set scoring (for example, scanpy score_genes^1,2^) is a simple and widely used approach to query which known gene programs are active in which cells, but it is often confounded by gene set overlap and technical factors. The regulation of gene programs tends to be shared across cell subpopulations, creating collinearity in gene expression and imbuing high-dimensional cell-by-gene count matrices with low-dimensional structure. Matrix factorization can mine this structure to identify candidate gene programs^3,4^ and is a core tool in single-cell analysis; for example, factorization by principal component analysis appears in most analysis pipelines.

In principle, the power of factorization lies in summarizing biological activity as a set of cellular building blocks (a minimal vector representing the degree to which a cell activates each gene program) rather than a noisy vector of all observed genes or a single label denoting cell type. Yet, there are many ways to decompose a matrix, and unsupervised approaches, such as principal component analysis and non-negative matrix factorization (NMF), produce factors that are often difficult to interpret or are driven by technical artifacts, such as batch effects, ambient RNA or gene expression scale differences^4,5^. Supervised approaches use known gene sets to make detected factors more interpretable^6,7^, but preexisting gene sets are typically defined in different biological contexts than those under study. In addition, cell-type factors tend to prevail in factor analysis because expression differences between cells are dominated by cell type^5^. The popular practice of partitioning data by cell type and factoring each subset separately mitigates this issue but makes it impossible to find shared programs.

We developed Spectra (supervised pathway deconvolution of interpretable gene programs) to provide meaningful annotations of cell function by balancing prior knowledge with data-driven discovery (https://github.com/dpeerlab/spectra). Spectra incorporates existing gene sets and cell-type labels as prior information, explicitly models cell type and represents input gene sets as a gene–gene knowledge graph using a penalty function to guide factorization toward the input graph. The graph representation enables data-driven modification of the input to reflect biological context and the identification of novel gene programs from residual unexplained variation. The degree of reliance on prior knowledge can be tuned with a global parameter.

The minimization of cell-type influence allows Spectra to identify factors that are shared across cell types. We show that Spectra outperforms existing approaches and solves longstanding challenges in tumor immune contexts, including the identification of an interpretable tumor reactivity factor in CD8^+^ T cells and a new invasion program in macrophages, which associate with response and resistance to cancer immunotherapy, respectively. Our open-source software scales to large atlases and overcomes batch effects to find factors that are stable across cohorts and even tumor types and are robust enough to be associated with clinical variables.

## Results

### Spectra identifies interpretable gene programs

**Fig. 1:**
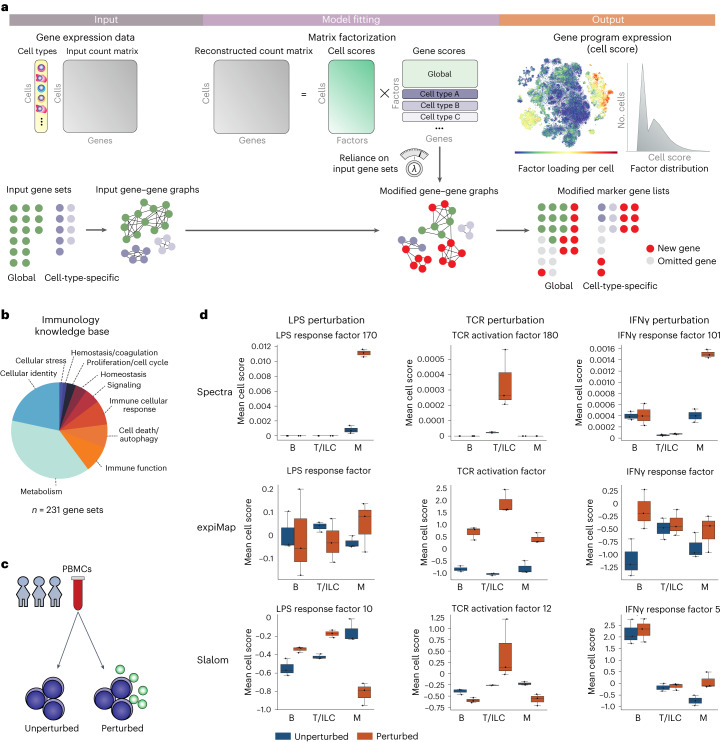
Spectra uses gene sets and cell types to guide gene program discovery from scRNA-seq data. **a**, As input, Spectra receives a gene expression count matrix with cell-type labels for each cell as well as predefined gene sets, which it converts to a gene–gene graph. The algorithm fits a factor analysis model using a loss function that optimizes reconstruction of the count matrix and guides factors to support the input gene–gene graph. As output, Spectra provides factor loadings (cell scores) and gene programs corresponding to cell types and cellular processes (factors). **b**, Gene set categories in the immunology knowledge base. **c**, Design of the perturbation experiments from Kartha et al.^8^. PBMCs (*n* = 23,754) from healthy human donors (*n* = 3) were incubated for 6 h with LPS, PMA or recombinant human IFNγ. **d**, Ability of different algorithms to identify gene programs associated with biological perturbations in the PBMC dataset. For select factors, mean per-donor cell scores are provided for T cells or innate lymphoid cells (T/ILCs), B cells (B) and myeloid cells (M; *n* = 3 donors). Boxes and lines represent interquartile range (IQR) and median, respectively; whiskers represent 1.5× IQR. Source data

We assume that each cell executes a small number of gene programs and that its observed expression is determined by the sum of its active programs. Spectra decomposes the cell-by-gene expression matrix into a cell-by-factor matrix that identifies and quantifies the programs executed by each cell and a factor-by-gene matrix representing the genes in each program (Fig. 1a and Methods). As input, the algorithm receives a normalized cell-by-gene count matrix, a cell-type annotation for each cell and either a list of gene sets or gene–gene relationships in the form of knowledge graphs. As output, Spectra provides a set of normalized global and cell-type-specific factor matrices that represent the gene loadings for each identified factor (gene scores), a sparse matrix of normalized factor loadings for each cell (cell scores) and a modified gene knowledge graph that represents factors inferred from the data (see Methods for a technical description of Spectra and parameter settings).

Two key features distinguish Spectra from other factorization methods, enabling it to identify more interpretable factors and discover new biology. First, Spectra uses known cell-type information and allows for cell-type-specific factors. Second, Spectra represents existing gene sets as an input gene–gene knowledge graph, enabling their data-driven modification and the derivation of entirely new factors.

Cell-type labels are provided as input to Spectra, which models the influence of a factor on gene expression relative to baseline expression per cell type, thereby mitigating its influence on the factors. The ability to incorporate cell-type-specific factors guides inference. For example, the T cell antigen receptor (TCR) activation program should be limited to T cells, but many of its genes are activated by additional programs in other cell types, which confuses traditional factor analysis.

Spectra attempts to balance prior knowledge and interpretability with faithfulness to the data. Its likelihood function ensures that the reconstituted matrix closely matches the input matrix, and its penalty function guides gene factorization toward the gene–gene knowledge graph (Methods). To capture prior knowledge, we use binary gene–gene relationships and encourage these gene pairs to share similar factors. Spectra takes input gene sets and turns each into a fully connected clique in the input graph, indicating their relationships. Factors are thus scored by how well they match the data and how many edges in the gene–gene graph support them.

Most gene sets are derived from multiple biological contexts, which differ from the context under study. Spectra can take a compilation of gene sets and determine the subset supported by the data. Encoding prior knowledge as a graph facilitates computational efficiency and allows Spectra to adapt gene programs by adding or removing edges in the input graph. The algorithm incorporates background edge and non-edge rates (provided as input parameters or learned from the data) to determine edge addition and removal rates. Critically, Spectra can detach factors from graph penalization to learn entirely new factors. In effect, Spectra attempts to explain as many of the input gene counts as possible by adapting the input gene graph (providing highly interpretable factors) and uses the residual unexplained counts to identify non-penalized factors that can capture entirely novel biology.

### Spectra factors predict ground truth signaling perturbations

We first curated a general resource of 231 immunological cell-type and cellular process gene sets that can be input into Spectra for analyzing any immune-related dataset (Fig. 1b, Supplementary Table 1 and Methods). To maximize how many processes can be dissected and to avoid size-driven effects, our cellular process gene sets have comparable size (median of 20 genes per set) and relatively little overlap (median of 40% pairwise overlap).

We used our immunology knowledge base to infer gene programs in a ground truth scRNA-seq dataset^8^ from human peripheral blood mononuclear cells (PBMCs) stimulated in vitro with interferon-γ (IFNγ), lipopolysaccharide (LPS) or phorbol myristate acetate (PMA), a protein kinase C activator used to mimic TCR activation (Fig. 1c). We ran Spectra in addition to expiMap^9^ and Slalom^6^ (factorization methods that also incorporate prior gene sets) and tested the association of factor cell scores with their corresponding perturbations. Only Spectra identified gene programs associated with all three perturbations in the correct condition and cell type (Fig. 1d), substantially outperforming Slalom and expiMap.

### Spectra identifies robust factors in immuno-oncology data

**Fig. 2:**
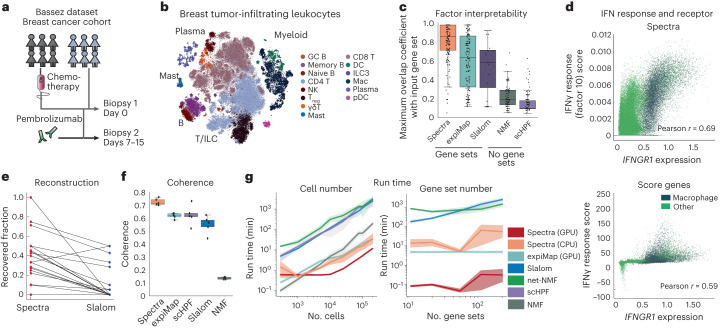
Evaluation of Spectra performance on simulated data and an immuno-oncology dataset. **a**, Treatment and scRNA-seq sampling regimen of individuals with breast cancer in the Bassez dataset^10^. **b**, t-Distributed stochastic neighbor embedding (t-SNE) of tumor-infiltrating leukocytes (*n* = 97,863 cells) from the Bassez dataset colored by cell type; B, B cell; DC, dendritic cell; γδT, γδ T cell; GC, germinal center; ILC3, type 3 innate lymphoid cell; Mac, macrophage; Mast, mast cell; NK, natural killer cell; pDC, plasmacytoid dendritic cell; Plasma, plasma cell; T, T cell; T_reg_, regulatory T cell. **c**, Maximum overlap coefficient of every global factor generated by Spectra (*n* = 152 global factors), expiMap^9^ (*n* = 155 factors, soft_mask = True), Slalom^6^ (*n* = 20 factors), NMF (*n* = 100 factors) and scHPF^4^ (*n* = 100 factors) with every input gene set. Boxes and lines represent IQR and median, respectively; whiskers represent 1.5× IQR range. **d**, Cell scores for Spectra and scanpy.score_genes^1,2^ factors plotted against MAGIC-imputed (*t* = 3) *IFNGR1* expression for each cell colored by cell type (*n* = 97,863 cells); IFN, interferon. **e**, Proportion of held-out genes recovered by Spectra or Slalom from the Bassez dataset for each input gene set tested. Lines connect identical input gene sets. **f**, Coherence (mean pairwise log-normalized co-occurrence rate among the top 50 markers) of factors generated by various factor analysis methods using a random sample of 10,000 cells from the Bassez dataset with 14 cell types and 20 input genes sets (Slalom) or 181 input gene sets (other methods). The experiment was repeated *n* = 5 times. Boxes and lines represent IQR and median, respectively; whiskers represent 1.5× IQR. **g**, Run time dependence on cell number with 35 gene sets (left) and gene set number with 25,000 cells (right). The experiment was performed using one cell type with the methods in **b** and netNMFsc (net-NMF)^7^ and was repeated *n* = 3 times; shading indicates 95% confidence interval; CPU, central processing unit. Source data

We next applied Spectra to scRNA-seq data from the challenging context of individuals with non-metastatic breast cancer before and after pembrolizumab (anti-PD-1) treatment (‘Bassez dataset’; Fig. 2a)^10^. The original study used clustering and gene set analysis to identify therapy-induced changes and used TCR sequencing to define the clonal T cell expansion status of each participant treated with anti-PD-1 as a surrogate for immune checkpoint therapy (ICT) response^10^.

**Extended Data Fig. 1:**
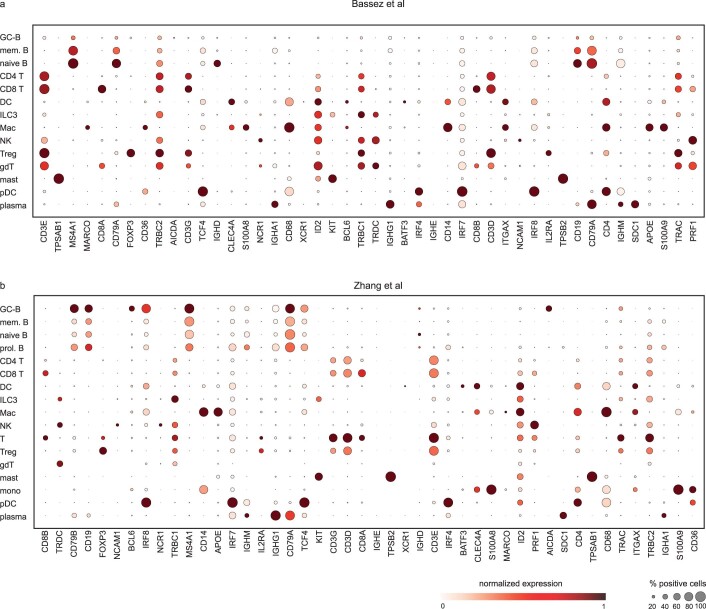
Cell type annotations in breast cancer datasets. Related to Fig. 2. Marker gene expression for 14 and 17 broad cell-type annotations in the Bassez (**a**; *n* = 97,863 cells) and Zhang (**b**; *n* = 150,985 cells) data, respectively. Each gene is normalized to its maximal expression, and the percentage of cells that express the gene within a cluster is indicated (% positive cells). ILC3, innate lymphoid cell type 3; T, T cell; gdT, gamma-delta T cell; pDC, plasmacytoid dendritic cell; NK, natural killer cell; B, B cell; mac, macrophage; mast, mast cell; mem, memory; mono, monocyte; prol, proliferating; Treg, regulatory T cell; DC, dendritic cell; GC-B, germinal center B cell; plasma, plasma cell. Source data

We annotated 14 broad cell types (including CD8^+^ T cells and macrophages), leaving Spectra to infer factors associated with finer cell-type distinctions, such as T cell activation or macrophage polarization (Fig. 2b, Extended Data Fig. 1, Supplementary Table 2 and Methods). Fitting the Spectra model with default parameters (Methods) and our cell-type labels and immunology knowledge base as input resulted in 152 global and 45 cell-type-specific factors, the latter including CD4^+^ T cells (*n* = 12), CD8^+^ T cells (*n* = 7) and myeloid cells (*n* = 6).

**Extended Data Fig. 2:**
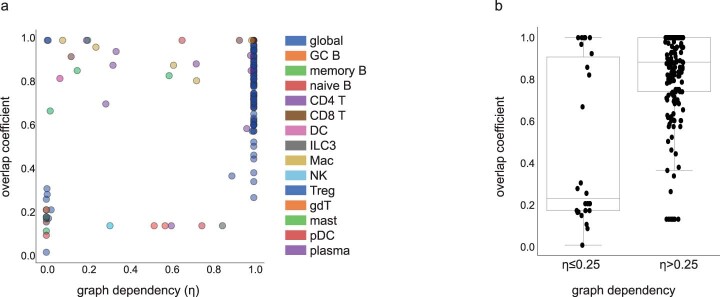
Dependence of Spectra factors on input gene sets. Related to Fig. 2. **a**, Maximum overlap coefficient for each factor (*n* = 197 factors), denoting overlap with input gene sets, plotted against Spectra’s graph dependency parameter (eta, *η*). Factors are colored by cell type. Most factors are highly dependent on the gene-gene graph and exhibit correspondingly high overlap coefficients. **b**, Maximum overlap coefficient of each factor (*n* = 197 factors) with input gene sets and a high (*η* ≥ 0.25) or low (*η* < 0.25) dependence parameter. Boxes and line represent interquartile range (IQR) and median, respectively; whiskers represent 1.5x IQR. ILC3, innate lymphoid cell type 3; T, T cell; gdT, gamma-delta T cell; pDC, plasmacytoid dendritic cell; NK, natural killer cell; B, B cell; mast, mast cell; Treg, regulatory T cell; DC, dendritic cell; GC B, germinal center B cell; plasma, plasma cell. Source data

We determined overlap with known gene sets to assess whether Spectra can identify biologically interpretable programs. For every factor, Spectra estimates a dependence parameter (*η*) that quantifies reliance on the gene–gene graph. Most factors (171) are strongly constrained by the graph (*η* ≥ 0.25), whereas 26 are novel (Extended Data Fig. 2). We found that factors with *η* ≥ 0.25 generally share over 50% of their genes with an input gene set, whereas the unbiased factorization approaches NMF and scHPF^4,5^ produce factors that do not agree with annotated gene sets (Fig. 2c), underscoring the difficulty of interpreting programs derived by these approaches.

**Extended Data Fig. 3:**
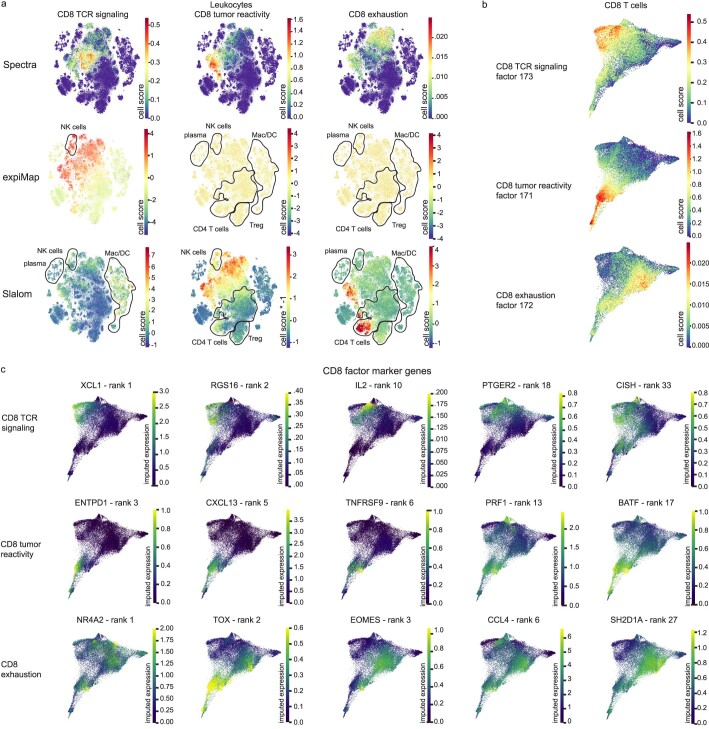
Spectra detects cell-type-specific gene programs. Related to Fig. 2. **a**, t-SNE embeddings colored by cell scores for the Spectra, expiMap or Slalom factors best representing CD8^+^ T-cell tumor reactivity and CD8^+^ TCR signaling (*n* = 97,863 cells). Black contours highlight aberrant expression in populations other than T cells. **b**, Force-directed layout (FDL) of CD8^+^ T cells (*n* = 31,925) colored by the cell scores of indicated factors. **c**, FDLs of CD8^+^ T cells (*n* = 31,925) colored by imputed expression (MAGIC *t* = 3) of relevant marker genes, ranked according to their gene scores. Many of these genes participate in multiple cellular processes (pleiotropy). For example, *CISH* is a member of the suppressor of cytokine signaling family that is induced by TCR activation, but also by IL-15 in NK cells, and is a critical regulator of dendritic cell differentiation. Source data

Spectra uses cell-type labels and cell-type-specific input gene sets to restrict factors to their appropriate cell type, ensuring more biologically sensible factor loadings; for example, Spectra limits CD8-specific TCR signaling, tumor reactivity and exhaustion factors to CD8^+^ T cells (Extended Data Fig. 3). By contrast, the gene set-based factorization method Slalom^6^ and autoencoder-based method expiMap^9^ misassign some TCR activity, CD8^+^ T cell exhaustion and tumor reactivity to the myeloid, natural killer (NK) cell and plasma cell lineages (Extended Data Fig. 3a), likely because many genes in these factors participate in multiple programs.

**Extended Data Fig. 4:**
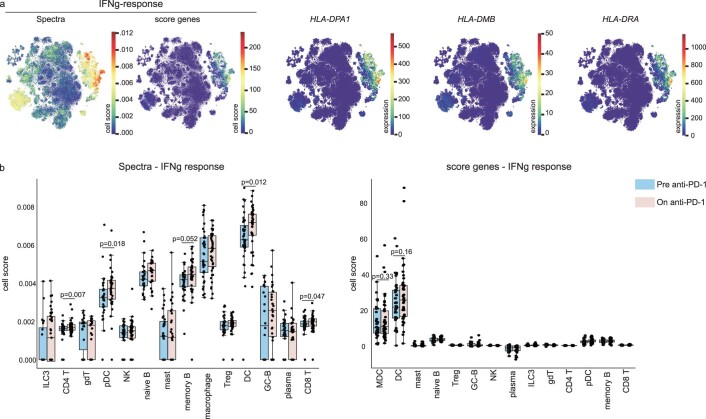
Spectra discerns the effects of immune checkpoint therapy on interferon signaling. Related to Fig. 2. **a**, t-SNE embeddings of tumor-infiltrating leukocytes (*n* = 97,863) from the Bassez data, colored by cell scores for Spectra or scanpy.score genes interferon gamma (IFNg) response, or by expression of selected human leukocyte antigen (HLA) class II genes. HLA expression is scran-normalized and not imputed. **b**, Mean cell score of the positive fraction (cell score > 0.01) per sample and cell type before (blue, patient sample number, n = 40) and after (red, patient sample number, *n* = 40) anti-PD-1 immune checkpoint blockade in breast tumor infiltrating leukocyte data from the Bassez data. Two-sided p values were calculated using Wilcoxon matched-pairs signed rank tests. Test statistics, left panel: 282 (Treg: regulatory T cell), 184 (memory B: memory B cell), 75 (mast: mast cell), 82 (pDC: plasmacytoid dendritic cell), 237 (CD4 T: CD4 T cell), 222 (DC: dendritic cell), 129 (naive B: naive B cell), 289 (NK: Natural Killer cell), 293 (CD8 T: CD8 T cell), 76 (gdT: gamma-delta T cell), 59 (GC-B: germinal center B cell), 339 (macrophage), 24 (ILC3: innate lymphoid cell type 3), 220 (plasma: plasma cell). Test statistics, right panel: 340 (Treg), 241 (memory B), 168 (mast), 131 (pDC), 362 (CD4 T), 305 (DC), 121 (naïve B), 349 (NK), 427 (CD8 T), 71 (gdT), 104 (GC-B), 356 (macrophage), 125 (ILC3), 262 (plasma). Cohen’s d, left panel: 0.049 (NK), 0.320 (CD4 T), 0.228 (GC B), 0.105 (CD8 T), 0.075 (naïve B), 0.064 (Treg), 0.285 (DC), 0.142 (mast), 0.093 (Mac), 0.131 (plasma), 0.171 (ILC3), 0.341 (pDC), 0.067 (gdT), 0.197 (memory B). Cohen’s d, right panel: 0.168 (NK), 0.142 (CD4 T), 0.039 (GC-B), 0.040 (CD8 T), 0.187 (naïve B), 0.008 (Treg), 0.166 (DC), 0.003 (mast), 0.064 (mac), 0.139 (plasma), 0.008 (ILC3), 0.202 (pDC), 0.170 (gdT), 0.146 (memory B). Boxes and line represent interquartile range (IQR) and median, respectively; whiskers represent 1.5x IQR. Source data

Pleiotropy similarly confounds score_genes^1,2^. For example, Spectra’s IFNγ response factor is well correlated with the gene encoding the IFNγ receptor upstream of this gene program and correctly captures it across all cell types, whereas the score_genes IFNγ response is detected almost exclusively in the myeloid population (Fig. 2d). This myeloid bias is due to differences in baseline expression across cell types, especially higher expression of genes encoding major histocompatibility complex class II (MHC class II) molecules by myeloid antigen-presenting cells (Extended Data Fig. 4). Spectra overcomes pleiotropy by implicitly downweighting the influence of genes whose expression could be explained by multiple factors. Specifically, Spectra decomposes gene expression using the factors best supported by total expression in a given cell. Spectra is able to identify IFNγ activity and its previously reported activation by ICT^11,12^ across expected immune cell types^13^ (Extended Data Fig. 4b) because it learns these factors in a cell-type-specific manner.

Thus, in addition to yielding more interpretable gene programs than other supervised methods, Spectra is better at inferring which cells these programs are active in.

### Spectra outperforms other methods on gene program benchmarks

**Extended Data Fig. 5:**
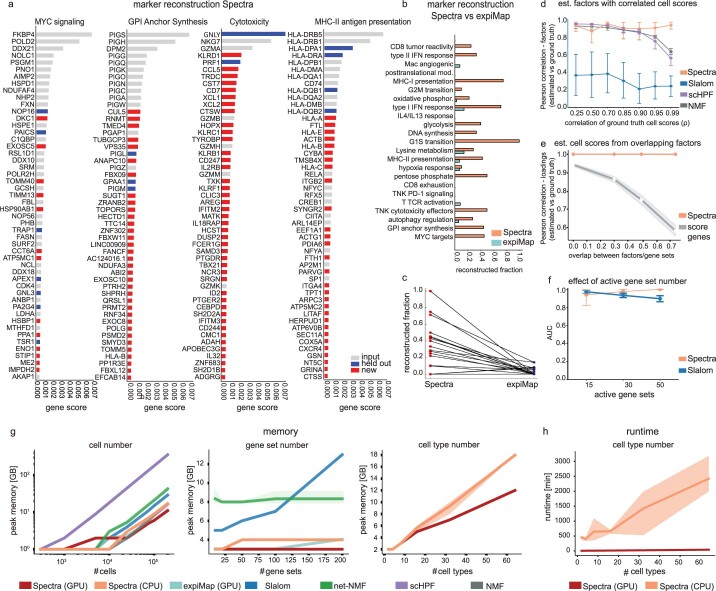
Spectra recovers genes involved in diverse cellular processes. Related to Fig. 2. **a**, Top 50 factor marker genes identified by Spectra in the Bassez data, after holding out a random 40% subset from each corresponding input gene set. Marker genes are ranked by Spectra factor score. New genes (red, absent from input gene set) and held-out genes (blue) recovered by Spectra; GPI, Glycosylphosphatidylinositol. **b**, Proportion of held-out genes recovered by Spectra or expiMap from the Bassez data, for individual gene set tested; phosphor., phosphorylation. **c**, Reconstruction performance on each tested gene set (*n* = 23). Lines connect identical gene sets. **d**, Synthetic data was generated by sampling random ground truth loadings and factors from log-normal and half-Cauchy distributions, respectively, and introducing correlations in two factors via off-diagonal entries in the log-normal covariance matrix. After multiplying loadings and factor matrices and introducing noise, models were fit to the data and output factors were correlated with ground truth factors; est., estimated. **e**, Correlation between ground truth and inferred loadings (cell scores) for score genes and Spectra versus gene set overlap in simulated data. Data consisted of overlapping synthetic gene sets and a random cell loading vector representing the expression of each gene set in a cell (Methods). The sum of gene sets weighted by the individual cell loadings for each cell was used to represent a mean for sampling Poisson gene expression counts. **f**, Gene expression data was simulated from a factor analysis model in which only a subset of gene sets are active in the data, similar to **d** and the original Slalom publication. AUC, area under the receiver operating characteristic (ROC) curve. Intervals and lines represent 95% confidence interval and mean, respectively, across *n* = 10 (**d**), *n* = 3 (**e**), and *n* = 5 (**f**) independent simulations. **g**, Memory dependence on cell (left panel, *n* = 35 gene sets, 1 cell type), gene set (middle panel, *n* = 25,000 cells, 1 cell type), and cell type number (right panel, *n* = 25,000 cells, one gene set per cell type and one global). **h**, Runtime dependence on cell type number. Each experiment (**g**,**h**) was repeated *n* = 3 times, shading indicates 95% confidence interval. Source data

We systematically benchmarked Spectra against other methods by measuring how well they identify coherent gene programs and assign activity to cells. A key feature of Spectra is that it can modify input gene sets in a data-driven manner. We held out 30% of genes from 20 input gene sets and tracked their identification in the resulting factors (Methods). Spectra factors recover many more genes than Slalom^6^ (Fig. 2e) and expiMap^9^ (Extended Data Fig. 5a–c). For example, among the 50 genes with the highest gene scores for the MYC factor, Spectra identified 7 of 33 held-out genes; moreover, it recovered additional MYC target genes *DKC1* (ref. ^14^) and *TOMM40* (ref. ^15^), which are absent from the training and hold-out sets, whereas MYC signaling was not captured by Slalom (Fig. 2e, Extended Data Fig. 5a and Methods).

To evaluate new gene detection, we reasoned that genes belonging to a program should exhibit coherence; that is, they should be coexpressed in the same cells. We applied factor analysis with held-out cells and evaluated the coherence of inferred factors in the test set (Methods). Spectra and other methods that take the sparsity of scRNA-seq data into account (Slalom and scHPF) perform well, whereas generic models (NMF) do not (Fig. 2f). The key advantage of supervised approaches is that by seeding inference with a known gene set, coherent genes are more likely to be biologically meaningful (Extended Data Fig. 5b,c).

Unlike other methods, Spectra’s use of prior knowledge enabled it to separate highly correlated factors in simulated data generated by a generic factor analysis model with both correlated and uncorrelated factors (Methods and Extended Data Fig. 5d). Estimating factor loadings in these data is particularly challenging because pleiotropy creates correlation between gene programs (Methods). As gene set overlap increases, score_genes^1,2^ surges in false-positive score estimates, whereas Spectra correctly assigns expressed factors to cells (Extended Data Fig. 5e). Due to their multivariate nature and encouragement of sparsity, factorization methods select the factors that best explain the data globally, such that each factor accounts for expression not already explained by other factors. Factor analysis is thus superior to score_genes even for the simple task of scoring gene sets.

In contrast to Spectra, Slalom’s accuracy drops substantially as the number of active gene sets increases (Extended Data Fig. 5f). Moreover, Slalom can only assess a few dozen gene sets before run time becomes prohibitive, whereas Spectra scales to hundreds of thousands of cells and hundreds of gene programs. When run on a graphics processing unit (GPU), Spectra outperforms all methods, including NMF and the GPU-based expiMap (Fig. 2g). Similarly, Spectra’s peak memory usage remains low with increasing gene set numbers (Extended Data Fig. 5g). Spectra run time and memory increase proportionally with the number of cell types and remain low for typical cell-type numbers (Extended Data Fig. 5g,h). Our benchmarking demonstrates that Spectra is faster and infers programs with superior interpretability and coherence while retrieving more ground truth factors.

### Spectra separates tumor reactivity and exhaustion features

To understand and ultimately improve therapeutic efficacy, we quantified therapy-induced gene program changes in non-dysfunctional tumor-reactive CD8^+^ T cells, a subset of T cells that recognize tumor-associated antigens^16^ and are also cytotoxic^17,18^. These cells express clonal TCRs and specific markers and accumulate after PD-1/PD-L1 checkpoint blockade (clonal expansion^19,20^). Conversely, T cells that expand clonally under ICT are likely to be tumor-reactive^18,19^. These cells may also gradually become exhausted (lose effector capacity) after prolonged antigen exposure in the tumor microenvironment^21,22^. Although exhaustion and tumor reactivity lead to different cellular behaviors with highly consequential phenotypes, their gene programs are correlated and challenging to discriminate computationally; clustering approaches typically group exhaustion, tumor reactivity and cytotoxicity features together^10,23^.

**Fig. 3:**
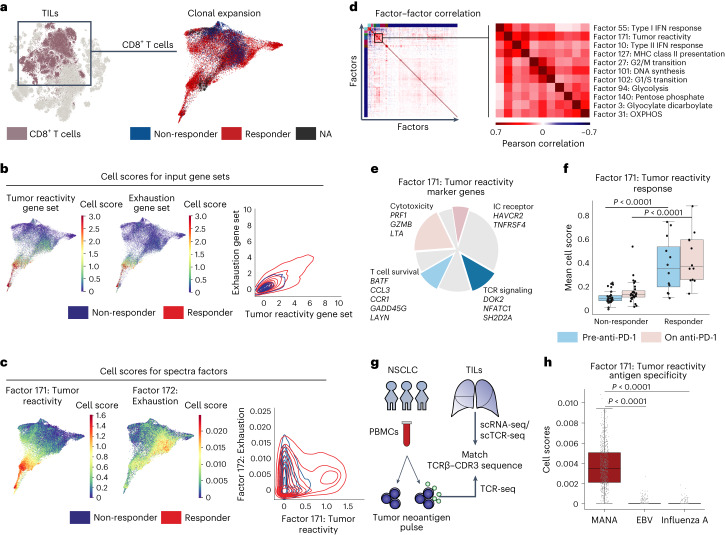
Spectra deconvolves the highly correlated features of tumor reactivity and exhaustion in CD8^+^ T cells. **a**–**h**, Analysis of tumor-infiltrating leukocytes (TILs) from the Bassez data^10^ (*n* = 42 participants). **a**, Left, t-SNE embeddings highlighting CD8^+^ T cells. Right, force-directed layout of CD8^+^ T cells (*n* = 31,925 cells) labeled by clonal T cell expansion (responder) under therapy; NA, not available. **b**,**c**, Force-directed layout of CD8^+^ T cells colored by tumor reactivity (left) or exhaustion (right) cell scores and contour plots depicting cell score density distribution. Cell scores were obtained using scanpy.score_genes^1,2^ (**b**) or Spectra (**c**). **d**, Pearson coefficients of factor cell scores (*n* = 31,925 cells). The inset displays factors that are highly correlated to tumor reactivity in CD8^+^ T cells; OXPHOS, oxidative phosphorylation. **e**, New genes identified by Spectra (*n* = 38) among the 50 highest scoring genes in the tumor reactivity factor, highlighting processes involved in tumor reactivity in different colors (also see Supplementary Table 3); IC, immune checkpoint. **f**, Per-sample mean cell scores for the Spectra tumor reactivity factor in positive cells (score > 0.001). Boxes and lines represent IQR and median, respectively; whiskers represent 1.5× IQR. Two-sided *P* values were calculated using Mann–Whitney *U*-tests; pre-anti-PD-1 (*n* = 40 participants): *P* = 3.84 × 10^–5^, statistic = 308, Cohen’s *d* = 1.51; on anti-PD-1 (*n* = 40 participants): *P* = 2 × 10^–5^, statistic = 313, Cohen’s *d* = 1.49. **g**, Design of the Caushi study^30^ (*n* = 251,777 CD8^+^ T cells), which profiled PBMCs and TILs from participants with non-small cell lung cancer (NSCLC). PBMCs were pulsed by peptide pools, and expanding TCR clones were identified by comparing PBMC and tumor-infiltrating leukocyte TCR sequences indicating their antigen specificity; scTCR-seq, single-cell TCR sequencing. **h**, Cell scores in tumor-infiltrating CD8^+^ T cells with different specificities (*n* = 1,151); EBV, Epstein–Barr virus. *P* values (two-sided) were calculated by using Mann–Whitney *U*-tests (MANA versus influenza A: *P* = 5.20 × 10^–101^, statistic = 148,361, Cohen’s *d* = 1.45; MANA versus Epstein–Barr virus: *P* = 6.04 × 10^–104^, statistic = 164,536, Cohen’s *d* = 1.44). Boxes and lines represent IQR and median, respectively; whiskers represent 1.5× IQR. Source data

**Extended Data Fig. 6:**
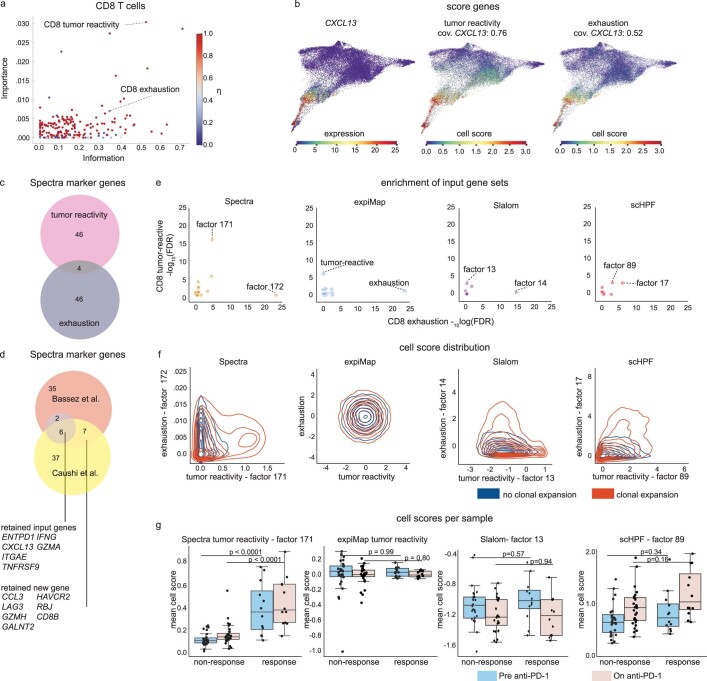
Existing methods fail to separate highly correlated features in CD8+ T cell data. Related to Fig. 3. Analysis of breast cancer infiltrating leukocytes from the Bassez data (*n* = 42 patients). **a**, Spectra information and importance scores (Methods) in CD8^+^ T cells, colored by *η* parameters. **b**, *CXCL13* expression is most correlated with tumor reactivity and exhaustion, and maps to similar cells as both gene set scores in force directed layouts (FDL) of CD8^+^ T cells (*n* = 31,925). Cov., covariance of each gene-set with *CXCL13*. **c**, Overlap between the top 50 marker genes of CD8^+^ T cell exhaustion and tumor reactivity factors. **d**, Overlap between the top 50 marker genes of the CD8^+^ T cell tumor reactivity factors in the Bassez and Caushi data. **e**, Significance (-log_10_(FDR)) of CD8^+^ T cell exhaustion or tumor reactivity factors by gene set enrichment analysis (Spectra, *n* = 159; Slalom, *n* = 20; scHPF, *n* = 100 factors); FDR, false discovery rate. **f**, Contour plots indicating density of Spectra, Slalom or scHPF loading scores for CD8^+^ T cell exhaustion and tumor reactivity factors grouped by clonal T cell expansion status (*n* = 31,925 cells). **g**, Per-sample mean cell scores for the tumor reactivity factor. Boxes and line represent IQR and median, respectively; whiskers represent 1.5x IQR. P values (two-sided) were calculated using Mann-Whitney *U* tests (*n* = 40 pre anti-PD-1, *n* = 40 on anti-PD-1 samples): Spectra: pre anti-PD-1: *p* = 3.84 x 10^-5^ statistic: 308 Cohen’s *d* (d): 1.510; on anti-PD-1: *p* = 2.00 x 10^-5^, statistic = 313 *d*: 1.491; expiMap, pre anti-PD-1 *p* = 0.99 statistic: 167 *d*: 0.194; on anti-PD-1 *p* = 0.80 statistic: 159 *d*: 0.150; Slalom pre anti-PD-1: *p* = 0.57 statistic: 198 *d*: 0.249 ; Slalom on anti-PD-1: *p* = 0.94 statistic: 155 *d*: 0.059; scHPF pre anti-PD-1: *p* = 0.34 statistic: 216 *d*: 0.337; scHPF on anti-PD-1: *p* = 0.16 statistic: 201 *d*: 0.592. Source data

We evaluated Spectra’s ability to deconvolve these programs, focusing on CD8^+^ T cells (Fig. 3a). The exhaustion and tumor reactivity factors scored high in Spectra’s information and importance scores (see Methods), suggesting that they explain relevant gene programs (Extended Data Fig. 6a). Genes from these two programs are correlated in these data (Extended Data Fig. 6b), explaining why they were not distinguished previously^10,23^. score_genes^1,2^ generates visually similar distributions of input gene sets in responders and non-responders (Fig. 3b), yet the absence of tumor-reactive, non-terminally exhausted states in responders is inconsistent with the treatment-induced clonal expansion of these states^19,20,24,25^, and it conflicts with the proven efficacy of ICT in this clinical setting^26^.

Whereas gene set scores fail to distinguish expanding from non-expanding clones (Fig. 3b), Spectra clearly disentangles them (Extended Data Fig. 6c), identifying a substantial tumor-reactive population that is almost exclusive to responders (Fig. 3c). Spectra extracts gene programs directly from the unlabeled data and does not need response status to successfully dissect these features. Spectra’s likelihood function discourages overlap between gene programs when a single program is sufficient to explain the observed count matrix, harnessing unique features of each gene set to associate cells with the best fit program. We identified *CXCL13* as the gene exhibiting the highest covariance with tumor reactivity as well as exhaustion factors (Extended Data Fig. 6b). Spectra assigns this tumor reactivity marker^27^ a high weight in tumor reactivity but not exhaustion and strongly weights genes related to TCR signaling, T cell activation and cytotoxicity in the tumor reactivity factor, whereas the exhaustion factor mostly includes genes encoding exhaustion-inducing transcription factors (*TOX*^21,22^ and *NR4A1* (ref. ^28^)) and *PDCD1* (PD-1) (refs. ^21,22^).

In CD8^+^ T cells, tumor reactivity correlates with proliferative programs, as expected for clonally expanding cells, oxidative phosphorylation and glycolysis, processes associated with enhanced CD8^+^ T cell effector function^29^ and IFNγ signaling, a key mediator of ICT efficacy^11^ (Fig. 3d). Of the top 50 marker genes in tumor-reactive CD8^+^ T cells, 42 are outside the input gene set, but recent studies support their roles in tumor reactivity (Fig. 3e and Supplementary Table 3)^30–35^.

Expression of this factor is higher in responders at baseline than in non-responders, and it increases further under therapy in responders (Fig. 3f), consistent with the reported association between tumor-reactive cell clusters and therapeutic response^36,37^. Spectra thus disentangles a CD8^+^ T cell tumor reactivity program that is associated with response to ICT at the cell and patient levels.

T cells kill cancer cells after binding to mutation-associated neoantigens (MANAs). To test whether our tumor reactivity program identifies T cells with MANA-specific TCRs, we leveraged a lung cancer atlas of tumor-infiltrating T cells with functionally validated TCR antigen specificity^30^ (‘Caushi dataset’; Fig. 3g). Spectra detected tumor reactivity and 172 additional factors in these data. Despite the different context and tumor type, 13 genes overlap among the top 50 marker genes in the Caushi and Bassez reactivity factors (Extended Data Fig. 6d). Moreover, the Caushi reactivity factor is almost exclusively expressed in T cells with a MANA-specific TCR rather than in T cells with TCRs for unrelated antigens (Fig. 3h). This independent, functionally validated dataset provides strong support for the Spectra tumor reactivity factors and suggests that transcriptional features of tumor-reactive T cells are shared across tumor types.

In contrast to Spectra, Slalom^6^, scHPF^4^ and expiMap^9^ failed to deconvolve the two factors (Extended Data Fig. 6e). Only Spectra was able to distinguish a clonally expanding tumor-reactive T cell population that is specific to responders (Extended Data Fig. 6f) and associates with patient-level response (Extended Data Fig. 6g).

Spectra is thus unique in its ability to disentangle tumor reactivity and exhaustion programs in CD8^+^ T cells, making it possible to identify tumor-reactive populations across cancer types and find novel mediators of tumor reactivity that can be associated with patient-level therapeutic responses and nominated as candidate targets for enhancing ICT efficacy.

### Spectra uncovers metabolic pathway use in leukocytes

**Fig. 4:**
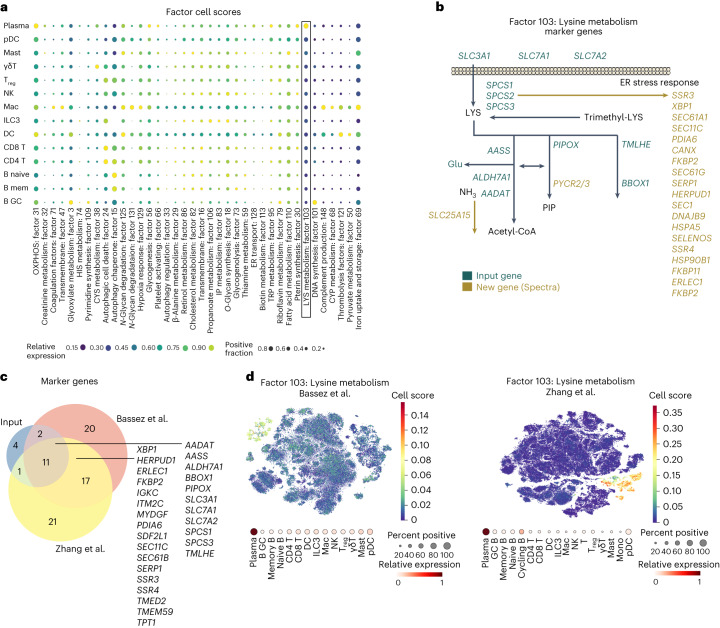
Spectra reveals cell-type-specific metabolic profiles in breast cancer data. **a**, Mean cell scores among positive (score > 0.01) cells normalized to maximum cell scores of each factor and positive fractions per cell type for each Spectra metabolic factor identified in the Bassez data^10^ (*n* = 97,863 leukocytes). The box highlights the plasma cell-enriched lysine (LYS) metabolism factor; CYP, cytochrome P450; CYS, cysteine; ER, endoplasmic reticulum; HIS, histidine; mem, memory; TRP, tryptophan. **b**, Input genes and genes newly inferred by Spectra in the lysine metabolism pathway; CoA, coenzyme A; Glu, glutamine; PIP, pipecolic acid. **c**, Overlap between the input lysine metabolism gene set and the top 50 marker genes from lysine metabolism factors identified in the Bassez^10^ and Zhang^23^ datasets. **d**, t-SNE embeddings of TILs colored by Spectra factor cell scores in the Bassez (*n* = 97,863 leukocytes) and Zhang (*n* = 150,985 leukocytes) datasets. Source data

Metabolic processes are fundamental to cancer therapeutic response, but metabolic genes participate in multiple pathways, making their analysis very challenging^38^. We tested Spectra’s metabolic inference on immune cells in the Bassez dataset^10^ and identified programs related to all 89 metabolic input gene sets (overlap coefficient of >0.25), recapitulating known macrophage characteristics, such as iron uptake, iron storage^39,40^ and cholesterol synthesis^41,42^ as well as DNA synthesis in cycling germinal center B cells (Fig. 4a).

**Extended Data Fig. 7:**
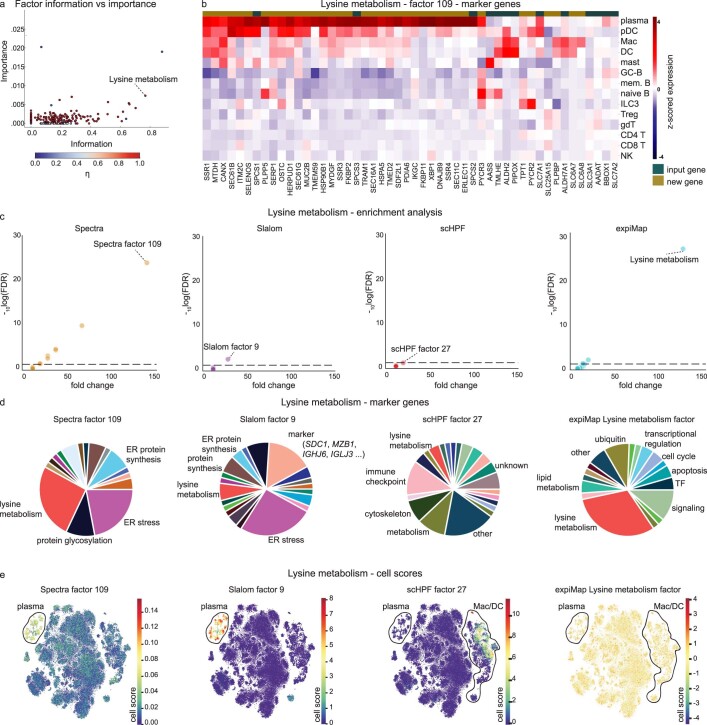
Spectra finds highly specific and biologically coherent lysine metabolism factors. Related to Fig. 4. **a**, Spectra importance and information scores for factors in plasma cells, colored by *η* parameter. **b**, z-scored average MAGIC imputed (*t* = 3) cellular expression (per cell type) of lysine factor genes (*n* = 97,863 leukocytes). **c**, Significance (-log_10_(FDR)) and fold enrichment (odds ratio) of the lysine metabolism input gene set, among the 50 genes with highest gene scores, as calculated by gene set enrichment analysis (Spectra: *n* = 152; Slalom: *n* = 20; scHPF: *n* = 100 factors). **d**, Functional categories of the top 50 marker genes of lysine metabolism factors identified by different factorization methods; TF, transcription factor. **e**, t-SNE embeddings colored by cell scores for top-performing lysine metabolism factors (labeled in **c**) from different factorization methods (*n* = 97,863 leukocytes). Plasma, macrophage (Mac) and dendritic cell (DC) populations are outlined. ILC3, innate lymphoid cell type 3; T, T cell; gdT, *γδ* T cell; pDC, plasmacytoid dendritic cell; mac, macrophage; mono, monocyte; NK, natural killer cell; B, B cell; mast, mast cell; Treg, regulatory T cell; DC, dendritic cell; GC B, germinal center B cell; plasma, plasma cell. Source data

Spectra also uncovered cell-type-specific expression of amino acid factors, such as lysine metabolism in plasma cells (Fig. 4a). Lysine is a scarce nutrient in malignant breast cancer tissue^43^. Lysine metabolism scored high in Spectra’s information and importance scores (Extended Data Fig. 7a). Its top 50 marker genes contain 72% of the input gene set, including all key metabolic enzymes (Fig. 4b), and Spectra added unfolded protein response genes, including the pivotal initiators *XBP1* and *ATF6* and their downstream targets (*ERLEC1* (ref. ^44^), *SDF2L1* (ref. ^45^), *HERPUD1* (ref. ^46^) and *PDIA6* (ref. ^47^)). These genes are expressed more coherently and at higher levels in plasma cells than in other cells, as expected for a gene program (Extended Data Fig. 7b). Endoplasmic reticulum stress regulates the capacity of plasma cells to produce immunoglobulins^48^, likely because large quantities of misfolded antibodies^48^ must be degraded, generating lysine^49^. Other methods identified factors that are either not enriched for lysine metabolism genes or are uniformly expressed across cells (Extended Data Fig. 7c–e).

To gauge stability and reproducibility, we fit an independent Spectra model onto data from individuals with metastatic breast cancer biopsied before and during paclitaxel chemotherapy with or without anti-PD-L1 treatment (Zhang dataset)^23^ using identical parameters (Extended Data Fig. 1b). Of the top 50 markers in the Bassez dataset^10^, 28 were also identified in the Zhang dataset (Fig. 4c), including 17 of the 37 new genes learned directly from both datasets and encompassing ER stress. Spectra lysine metabolism factors from both datasets are specifically expressed in plasma cells (Fig. 4d). Our results link lysine metabolism and ER stress as features of tumor-infiltrating plasma cells in breast cancer.

### Macrophage states change continuously under therapy

**Extended Data Fig. 8:**
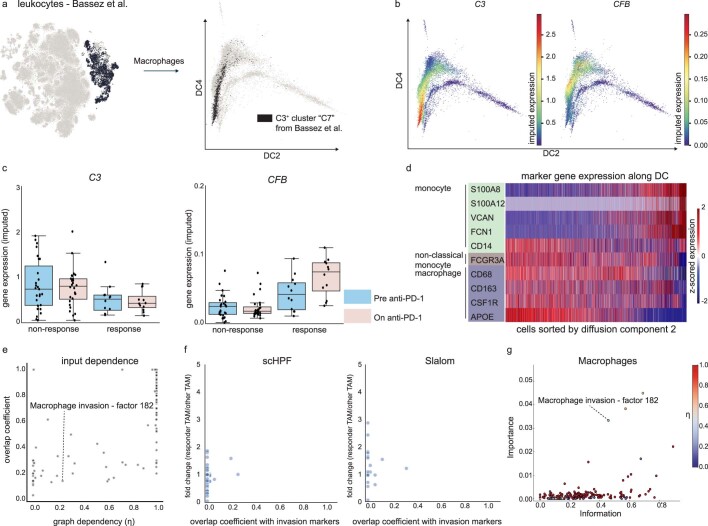
Tumor-infiltrating macrophage cell states exist along continua that change under therapy. Related to Fig. 5. **a**, t-SNE embedding of all leukocytes (left) and distribution of macrophages/monocytes (*n* = 12,132) along diffusion components 2 and 4 (DC2 and DC4, right), highlighting cells of *C3*-positive macrophage cluster C7 from the Bassez data. **b**, Macrophages along DC2 and DC4, colored by MAGIC-imputed (*t* = 3) complement gene expression. **c**, Mean MAGIC-imputed (*t* = 3) complement gene expression per sample in macrophages from responsive or non-responsive patients sampled before (pre, *n* = 40) or during (on, *n* = 40) anti-PD-1 therapy. Boxes and line represent IQR and median, respectively; whiskers represent 1.5x IQR. **d**, Scran-normalized expression of macrophage and monocyte marker genes in cells sorted along DC2, showing diverging expression along this gradient. **e**, Overlap coefficient and graph dependency for Spectra factors (*n* = 197). **f**, scHPF (*n* = 100) and Slalom (*n* = 20) factors do not resemble the Spectra invasion factor (factor 182). Each factor is plotted by fold change of its cell score in macrophage neighborhoods enriched in non-responders under therapy, compared to its cell score in all remaining macrophages, and by the coefficient of overlap between the top 50 marker genes of each factor and of the Spectra invasion factor (analogous to Fig. 5c,d). **g**, Spectra importance and information scores for factors in macrophages. Factors (points) colored by eta parameter indicated in color code. Source data

Macrophages mediate resistance to ICT by becoming immunosuppressive under therapy (adaptive resistance); however, the effect of ICT on macrophage gene programs and the association with response remains unclear^50,51^. Bassez et al.^10^ linked a macrophage cluster expressing the complement gene *C3* to therapy resistance (Extended Data Fig. 8a,b); yet, complement genes such as *CFB* (which activates *C3* (ref. ^52^)) exhibit opposite trends to *C3* and are more highly expressed in responders (Extended Data Fig. 8b,c).

**Fig. 5:**
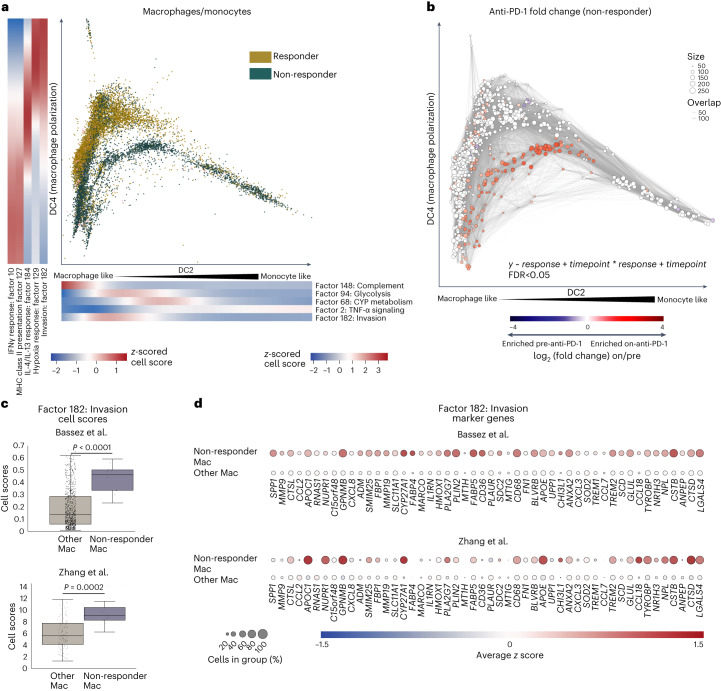
Spectra reveals therapy-induced macrophage gene expression programs. **a**, Macrophage cells plotted along DCs 2 and 4 colored by patient-level T cell expansion status (responder and non-responder) in the Bassez data^10^ (*n* = 12,132 cells). Heat maps indicate *z*-scored gene program cell scores along DCs smoothened by fitting a generalized additive model (Methods); IL, interleukin; TNF, tumor necrosis factor. **b**, Graph with nodes representing cellular neighborhoods (*n* = 858) plotted along DC2 and DC4 and edges representing overlap colored by log_2_ (fold change) under anti-PD-1 treatment, as estimated with Milo (Methods). The log_2_ (fold change) of non-significant (false discovery rate (FDR) ≥ 0.05) neighborhoods is set to 0. **c**, Average cell scores of macrophage neighborhoods (*n* = 858) enriched in non-responders under therapy and cell scores for all other macrophage neighborhoods in the independent Bassez and Zhang breast cancer datasets. Cell scores were calculated using the Spectra invasion factor (factor 182 from Bassez et al.^10^) or by using scanpy.score_genes^1,2^ on the top 50 marker genes of factor 182 in Zhang et al.^23^. *P* values (two-sided) were calculated using Mann–Whitney *U-*tests (Bassez: *P* = 4.96 × 10^–5^, statistic = 1,060, Cohen’s *d* = 1.49; Zhang: *P* = 3.74 × 10^–12^, statistic = 600,886, Cohen’s *d* = 1.03). Boxes and lines represent IQR and median, respectively; whiskers represent 1.5× IQR. **d**, Mean expression *z* scored across cells (*n* = 12,132 cells) and percentage of cells with at least one detected copy of the indicated factor genes in non-responder macrophage populations and other macrophage populations in the Bassez (*n* = 12,132 cells) and Zhang (*n* = 3,206 cells) datasets. Source data

To determine whether Spectra can identify more interpretable gene programs underlying adaptive resistance, we used diffusion components (DCs) to visualize continuous states^53^. DC2 captures maturation from monocyte-like to macrophage states, and DC4 separates responders from non-responders (Fig. 5a and Extended Data Fig. 8d). Cell scores for Spectra factors form gradients along DC2, with successive peaks of tumor necrosis factor-α (TNF-α) signaling and CYP enzyme activity, followed by glycolytic activity^54^, a novel factor containing invasive and angiogenic mediators (‘invasion program’) and finally complement production, a key feature of mature macrophages^55^. Along DC4, Spectra identified programs for type 2 IFN signaling and MHC class II antigen presentation at one extreme, followed by the interleukin-4 (IL-4)/IL-13 response, hypoxia signaling and the invasion program at the other (Fig. 5a; see Supplementary Table 4 for all DC-associated factors).

To find states that change in non-responders under ICT and could therefore confer adaptive resistance, we used Milo^56^, which revealed overlapping cellular neighborhoods (states) that only expand under anti-PD-1 therapy in non-responders (Fig. 5b) and are high in the novel invasion program (Fig. 5c). This invasion program does not correspond to input gene sets (*η* = 0.24) but has high importance and information scores; moreover, Slalom^6^ and scHPF^4^ do not identify a similar program (Extended Data Fig. 8e–g). Its constituent genes are coherently expressed in macrophages, only increase in non-responders and include genes encoding known invasion and metastasis mediators (*CTSL*^57^, *CTSD*^58^, *CTSB*^59^, *CHI3L1* (ref. ^60^), *SPP1* (ref. ^61^) and *PLIN2* (ref. ^62^)). Furthermore, the invasion program includes genes of inflammation modulators (*TREM1* (ref. ^63^), *TREM2* (ref. ^64^) and *GPNMB*^65^) and cholesterol metabolism genes (*APOE*^66,67^, *APOC1* (ref. ^68^) and *CYP27A1* (ref. ^69^)), some of which suppress inflammatory cytokine (IL-6 and TNF-α) release^65^. Our results suggest that in individuals who do not respond to ICT, macrophages may upregulate these genes coordinately (Fig. 5d). By focusing on residual expression that is not well explained by the gene knowledge graph, Spectra can thus find a gene program that is both interpretable and related to ICT response.

To test for replication, we ran Milo, identified macrophage populations in the Zhang dataset^23^ and scored expression of the top 50 invasion factor genes. Despite the different setting of metastatic tumors, the invasion and cholesterol metabolism genes identified in the Bassez data have high expression in the Zhang data, validating our invasion program (Fig. 5c,d). Spectra thus identifies a prometastatic gene program that is upregulated following anti-PD-1/PD-L1 treatment in individuals with therapy-resistant breast cancer, with implications for understanding adaptive resistance mechanisms and macrophage polarization.

### Spectra factors generalize to hundreds of individuals

Batch correction of technical differences between samples and cohorts tends to remove subtle, yet important, biological signals^70^, so we asked whether Spectra can find shared features without explicit batch correction.

**Fig. 6:**
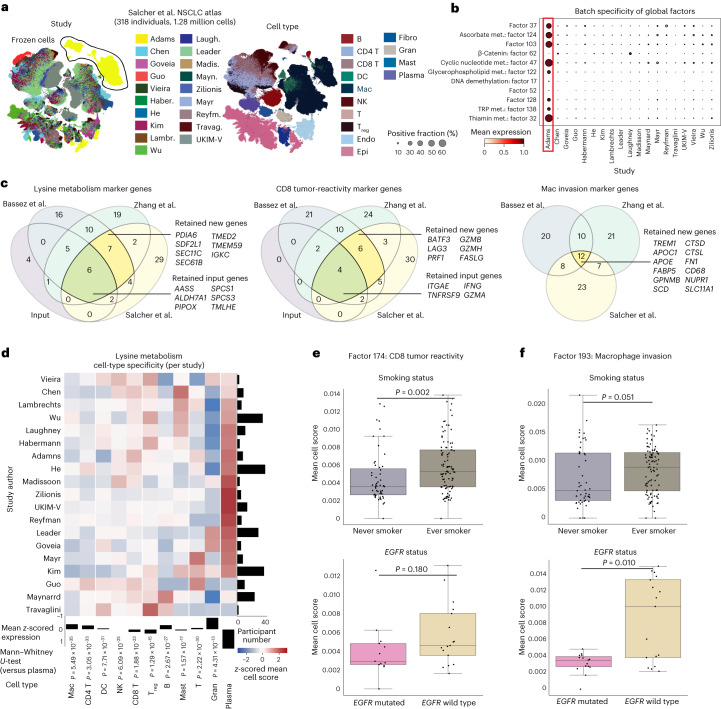
Spectra gene programs are reproducible across multiple studies. **a**, Uniform manifold approximation and projection (UMAP) embeddings of whole tumor single cell suspensions (*n* = 1.28 million cells) colored by study (left) or cell type (right) in the Salcher atlas^71^; Endo, endothelial; Epi, epithelial; Fibro, fibroblast; Gran, granulocyte; Haber., Habermann; Lambr., Lambrechts; Laugh., Laughney; Madis., Madissoon; Mayn., Maynard; Reyfm., Reyfman; Travag., Travaglini. **b**, Expression and positive cell fraction of global Spectra factors with the lowest entropy across studies. The Adams study with batch effect is highlighted in red; met., metabolism. **c**, Overlap between the input gene set and the top 50 marker genes for lysine metabolism (left), tumor reactivity (middle) and macrophage invasion (right; new factor, no input set) factors. **d**, Mean cell scores, z-scored across cell type, of the lysine metabolism factor per study and cell type. Bars indicate mean *z* score per column (bottom) and participant numbers per study (right). Two-sided *P* values between plasma cells and other cell types were calculated using Wilcoxon matched-pairs signed-rank tests. (B cells: statistic = 2,903, Cohen’s *d* = 0.77; CD4^+^ T cells: statistic = 2,385, Cohen’s *d* = 0.88; CD8^+^ T cells: statistic = 4,555, Cohen’s *d* = 0.70; dendritic cells: statistic = 3,152, Cohen’s *d* = 0.76; granulocytes: statistic = 516, Cohen’s *d* = 0.91; macrophages: statistic = 2,350, Cohen’s *d* = 0.86; mast cells: statistic = 5,348, Cohen’s *d* = 0.52; NK cells: statistic = 3,883, Cohen’s *d* = 0.70; regulatory T cells: statistic = 4,441, Cohen’s *d* = 0.61; T cells: statistic = 3,345, Cohen’s *d* = 0.56). The studies listed in **a**, **b** and **d** are from Salcher et al.^71^. **e**,**f**, Mean cell scores per patient in positive (>0.001) CD8^+^ T cells (**e**) or macrophages (**f**) for the tumor reactivity factor (**e**) and the macrophage invasion factor (**f**) based on smoking (top) or *EGFR* mutation (bottom) status. *P* values were calculated using Mann–Whitney *U*-tests (two-sided); tumor reactivity smoking: *n* = 153, *P* = 0.0022, statistic = 3,500, Cohen’s *d* = 0.45; tumor reactivity *EGFR*: *n* = 30, *P* = 0.18, statistic = 78, Cohen’s *d* = 0.52; invasion smoking: *n* = 147, *P* = 0.051, statistic = 2,928, Cohen’s *d* = 0.30; invasion *EGFR*: *n* = 32, *P* = 0.010, statistic = 59, Cohen’s *d* = 1.17). Boxes and lines represent IQR and median, respectively; whiskers represent 1.5× IQR. Source data

The scRNA-seq lung cancer atlas from Salcher et al.^71^ is composed of 1.28 million cells from 19 studies and 318 individuals, including a study that uses cryopreserved cells and exhibits a strong batch effect (Fig. 6a). We applied Spectra with default parameters and our immunology knowledge base and found 11 global factors with low cross-study entropy (Methods), 10 of which are specific to the cryopreserved cell study and account for its batch-driven variation (Fig. 6b).

Spectra identified lysine metabolism, CD8^+^ T cell-specific tumor reactivity and macrophage-specific invasion factors in the Salcher atlas without batch correction. Despite differences in tumor type and clinical cohort, multiple factor genes are shared across the Bassez, Zhang and Salcher datasets (Fig. 6c). Newly discovered shared genes include ER stress transcription factors *XBP1* and *ATF6* and targets (*SDF2L1* (ref. ^45^) and *PDIA6* (ref. ^47^; lysine metabolism factor)), the TCR signaling target *BATF*^31,35^ and the immune checkpoint gene *LAG3* (refs*.* ^32,72^; tumor reactivity factor), invasion mediators *CTSL* and *CTSD*^57,58^ and inflammatory mediators *TREM1* (ref. ^63^) and *GPNMB*^65^ (macrophage invasion factor). The identified factors are very stable across the Salcher atlas, and lysine metabolism is significantly enriched in plasma cells (13 of 19 studies, *P* < 10^−12^), as observed in breast cancer (Fig. 6d).

Next, we tested for associations between Spectra factors and two clinically important variables, *EGFR* mutation and smoking status. Although *EGFR*-mutated tumors are resistant to ICT^73^, smokers respond more frequently^74^. Tumor reactivity cell scores are higher in CD8^+^ T cells from tumors of smokers than from tumors of non-smokers (*P* = 0.002) and are higher in wild-type *EGFR* tumors than in mutated tumors (*P* = 0.180; Fig. 6e). The invasion factor similarly shows higher cell scores in macrophages from smokers (*P* = 0.051) and wild-type *EGFR* tumors (*P* = 0.010; Fig. 6f). In the breast cancer datasets, this factor is associated with ICT resistance (Fig. 5c), and studies of its marker genes suggest that they are involved in suppressing antitumor immunity (*FABP5* (ref. ^75^) and *TREM1* (ref. ^63^)).

Spectra thus finds subtle programs across batches and patients without requiring explicit batch correction. Although patient- or sample-level phenotypic association has been attempted with cell-type fractions, Spectra factors make it possible to associate clinical phenotypes with cell-type-specific gene programs, a promising strategy for cancer research and biomarker discovery.

## Discussion

Spectra anchors data-driven factorization with prior knowledge to infer factors that are coherently expressed, interpretable and not polluted by cell-type markers. The algorithm modifies each factor to the dataset’s biological context by upweighting novel genes that are tightly expressed with factor genes, and it can dissect highly correlated factors, such as T cell exhaustion and tumor reactivity. We demonstrate that tumor reactivity program expression separates individuals with breast cancer by their clonal expansion status after anti-PD-1 treatment (other methods fail) and is replicated in a lung cancer setting with functionally validated T cell specificity.

We found that differences related to cell type dominate the marginal gene–gene covariance matrix, obscuring higher-resolution cell-type-conditional covariance structure. Spectra uniquely addresses this multiscale expression variance by accepting cell-type labels as input and explicitly modeling cell-type-specific factors that can account for local correlation patterns. As a result, Spectra reliably identifies programs that are conserved across multiple cell types related to metabolism, response to cytokine signaling, differentiation and growth and separately estimates the cell-type-specific components of these programs.

Our knowledge base of high-confidence gene sets can improve immune scRNA-seq data analysis using any supervised method, but Spectra does not strictly need good relevant gene sets; it adaptively tunes its reliance on prior information based on concordance of the input graph with observed data, and it allocates novel factors when prior information does not fully explain expression. This property allowed us to discover a cancer invasion program describing an axis of variation in tumor-associated macrophages that is strongly related to anti-PD-1 therapy resistance and is replicated in two independent datasets.

The common simplifying assumption made by factor analysis methods is that factors combine linearly to drive expression, which is not always the case. Uncovering interpretable nonlinear relationships is a future goal of factorization methods development.

We designed Spectra to unravel heterogeneity in large-scale scRNA-seq studies. Spectra factors are stable across two breast cancer datasets and a lung cancer atlas totaling over 1.5 million cells from 375 individuals and 21 studies, demonstrating the ability to find robust biological signal and overcome batch effects at this scale. Spectra factors make it possible to associate clinical covariates with cell-type-specific gene programs. In addition, the ability to transfer factors learned from one dataset to another can advance our ability to iteratively transfer and refine knowledge across scRNA-seq studies without requiring data integration.

## Methods

### Overview of Spectra

Spectra (https://github.com/dpeerlab/spectra) grounds data-driven factors with prior biological knowledge (Supplementary Fig. 1). First, Spectra takes in prior biological information in the form of cell-type labels and explicitly models separate cell-type-specific factors that can account for local correlation patterns. This explicit separation of cell-type-specific and global factors enables the estimation of factors at multiple scales of resolution. Second, Spectra resolves indeterminacy of the reconstruction loss function via a penalty derived from a gene–gene knowledge graph that encourages solutions that assign similar latent representations to genes with edges between them. To account for prior information of variable relevance and quality, Spectra adaptively tunes its reliance on prior information based on concordance of the prior and observed expression data. Third, novel factors are adaptively allocated when prior information is insufficient to explain the observed expression data.

In the first step of Spectra, a set of gene–gene similarity graphs is built by aggregating information across gene sets and/or other sources. This graph representation is flexible and can accommodate various types of prior knowledge; gene sets can be incorporated into graphs by including edges between genes that are annotated to the same pathway, whereas existing datasets can be used to generate annotations by thresholding partial correlations or factor similarity scores. This representation lends computational convenience, as the graph dimensions are fixed regardless of the size of the input annotations. The annotations are either labeled as cell-type-specific or have global scope. A separate graph is thus built for each cell type alongside a global graph.

In the second step, Spectra learns a multidimensional parameter for each cell and each gene, representing each cell and each gene’s distribution over gene expression programs. Similarity of the parameters between genes indicates that these genes are likely to have an edge joining them, whereas similarity of the parameters between a cell and a gene indicates that the cell is likely to express that gene. Hence, the graph encodes the prior that genes with edges between them are likely to be expressed by the same set of cells. In practice, we take several additional steps to fulfill the desiderata: (1) factors not represented in the annotations can be discovered, (2) low-quality annotations can be removed, and (3) discrete cell types are assumed to be fixed and known and therefore not captured as factors by the model.

To avoid penalizing novel factors that have no relation to the annotations, we introduce a weighting matrix that scales the computation of gene–gene similarity scores by factor-specific weights that are learned from the data. Factors that have low weight are not used in computing edge probabilities, whereas factors with high weights influence the edge probabilities directly. Hence, Spectra can estimate similar parameters for two genes without forcing a high edge probability between them as long as the factors corresponding to these genes also have low weight. These weights allow the addition of new, unbiased factors that are not influenced by the input annotations. Importantly, weights are estimated from the data, allowing for an adaptive determination of the relative number of unbiased and biased factors. An estimated background rate of edges in the graph allows for the removal of annotations with little supporting evidence from gene expression data. Finally, Spectra explicitly separates global and cell-type-specific factors by enforcing a cell-type-determined block sparsity pattern in the cell loading matrix. Cell-type-specific factors capture within-cell-type variation, whereas global factors capture any variation that is shared across multiple cell types. To reduce the burden of modeling constitutively expressed cell-type marker genes, each factor’s contribution to gene expression is multiplied by a cell-type-specific gene weight. These cell-type-specific gene weights explain away the influence of cell-type marker genes and hence mitigate the tendency of these marker genes to influence the factors themselves.

### Components of the Spectra objective function

Broadly speaking, Spectra fits a set of factors and cell scores by minimizing an objective function with two components. The first component of the objective function, ${{{{\mathcal{L}}}}}_{{{{\rm{Reconstruction}}}}}$, measures how well the estimated model parameters can reconstruct (or predict) the observed expression data using the set of all model parameters Θ. We write ${{{{\mathcal{L}}}}}_{{{{\rm{Reconstruction}}}}}(\Theta )$ to emphasize that ${{{{\mathcal{L}}}}}_{{{{\rm{Reconstruction}}}}}$ is a function that maps a set of model parameters to a corresponding objective value. The second component of the objective function measures how well the set of model parameters Θ corresponds to our biological prior information. This second component is denoted ${{{{\mathcal{L}}}}}_{{{{\rm{Graph}}}}}(\Theta )$. We weight this term by a user-defined hyperparameter *λ*, which allows a user to control the level of confidence placed in the given biological prior information. The general form of the Spectra objective function is

$${{{\mathcal{L}}}}(\Theta )=\lambda {{{{\mathcal{L}}}}}_{{{{\rm{Reconstruction}}}}}(\Theta )+{{{{\mathcal{L}}}}}_{{{{\rm{Graph}}}}}(\Theta )$$

Below, we describe the precise functional forms of each of the objective function components.

### ${\boldsymbol{\mathcal{L}}}_{{{{\mathbf{Reconstruction}}}}}$ (Θ): modeling gene expression as a low-rank product

We assume that the expression variation observed in the count matrix is driven by variation in the activity of different biologically meaningful gene programs as well as technical variation that often involves highly expressed genes. Therefore, our model of gene expression needs to account for both components. In more detail, interpretation of factors estimated from scRNA-seq data is often hindered by highly expressed genes, which factor analysis methods based on reconstruction loss functions must account for. Housekeeping genes required for basal cellular function, such as *GAPDH*, *ACTB* and ribosomal genes, are expressed at high levels and hence unduly influence the reconstruction loss function despite the fact that their expression variance is explained in large part by overall levels of transcription. As a result, existing matrix decomposition methods tend to put high weight on such nonspecifically expressed genes, although post hoc corrections can be applied for the interpretation of individual factors. However, certain important cytokine genes (for example, *IL4*, *IL6*, *IL2* and *IL10*), chemokine receptor genes (*CXCR1* and *CXCR2*) and transcription factor genes (*RORC* and *BATF3*) are expressed in low mRNA copy numbers. Normalization strategies that rescale features empirically tend to amplify measurement uncertainty associated with lowly expressed genes, leading matrix factorization methods to overfit and return low-quality gene expression programs. To address this, we introduce gene scale factors *g*_*j*_ that are estimated from the data and allow the model to explain high expression and variability of certain genes without increasing the magnitude of the gene factor weights. Because lowly expressed genes are correspondingly noisier, we bound the minimum gene scale factors below by a tuning parameter *δ*.

By way of notation, **X** refers to the processed gene expression matrix, with entry **X**_*ij*_ containing the gene expression value for cell *i* and gene *j*. The matrix **X** has *n* rows (the number of cells) and *p* columns (the number of genes). *K* refers to the number of gene expression programs unless otherwise specified. Additionally, for a given cell indexed by *i*, the cell loading (a set of weights across the set of factors) is denoted by *α*_*i*_. The distribution across factors for gene *j* is denoted as *θ*_*j*_, which sums to 1 over *K* gene expression programs, $\mathop{\sum }\nolimits_{k = 1}^{K}{\theta }_{jk}=1$. Unsubscripted variables refer to the collection containing all possible subscripts; for example, *θ* refers to the collection of all *θ*_*j*_. The base expression model describing the gene expression measurement for cell *i* and gene *j* is

$${\mathbb{E}}[{{{{\bf{X}}}}}_{ij}]=({g}_{j}+\delta ){\alpha }_{i}^{\top }{\theta }_{j}$$

with *g*_*j*_ ∈ [0, 1] a gene scaling parameter, ${\alpha }_{i}\in {{\mathbb{R}}}_{+}^{K}$ and *θ*_*j*_ ∈ Δ^*K*−1^ (where Δ^*K*−1^ is the set of positive *K* − vectors that sum to 1). The low-rank decomposition of this expression model can be visualized in Supplementary Fig. 2.

#### Incorporating cell types into modeling expression variation

Because expression variation is dominated by cell types, existing methods generally fit factors that are polluted with cell-type markers or alternatively must be run on a subset of the data. For example, TCR activation programs (consisting of marker genes such as *NFATC1* and *NFATC2*) are confounded with T cell identity, and existing factor analysis methods tend to return identity marker genes, such as *CD3*, *CD4* and *CD8*. Similarly, programs representing metabolic pathways are often confounded with plasmacytoid dendritic cell (*IL3R* and *BDCA2*) or B cell (*CD19* and *CD79A*) identity marker genes. Although it is challenging to fit a biologically meaningful factor model, successful cell typing of scRNA-seq data using clustering approaches is a solved problem for discrete cell types but not for intermediate states. Therefore, to mitigate this issue, Spectra assumes that discrete cell types are known and therefore not captured as factors by the model; instead, Spectra explicitly fits cell-type-specific and global factors, allowing Spectra to effectively deal with expression variance at multiple scales. To perform this cell-type-integrative factor analysis, for cell type *c* and cell *i*, the model is extended to

$${\mathbb{E}}[{{{{\bf{X}}}}}_{cij}]=({g}_{j}+\delta ){\alpha }_{c,i,:K}^{\top }{\theta }_{j}+({g}_{cj}+\delta ){\alpha }_{c,i,K+1:}^{\top }{\theta }_{cj}$$

where *c* is the cell-type label for cell *i*, *g*_*cj*_ is cell-type-specific gene scaling, and ${\theta }_{cj}\in {\Delta }^{{K}_{c}-1}$ is a cell-type-specific gene representation with ${\alpha }_{c,i}\in {{\mathbb{R}}}^{K+{K}_{c}}$. Single-subscript variables, such as *g*_*j*_ and *θ*_*j*_, denote global parameters, whereas the notation *α*_:*K*_ indicates the first *K* elements of a vector (typically denoting global elements), and *α*_*K*+1:_ indicates the tail of the vector from the *K* + 1st element (typically denoting cell-type-specific elements). The threshold *δ* restricts the maximum ratio of gene scaling factors to $\frac{1+\delta }{\delta }$.

Spectra models the presence of gene programs with highly limited scope in that they can only be activated by a specific cell type, which can be represented by a hard-coded sparsity pattern in the cell loading matrix (Supplementary Fig. 3). The cell-type-specific gene scalings (*g*_*cj*_) associated with these programs are encouraged to capture cell-type identity markers and constitutively active genes, enabling factors themselves to capture variation across cell types and within cell types (Supplementary Fig. 4). Spectra tends to assign constitutive genes, such as *EEF1A1* and *ACTB*, and identity marker genes, such as *CD4* and *CD3*, high values of *g*_*j*_. Lowly expressed genes important for CD4^+^ T cell-specific gene programs, such as *IL21*, *IL13* and *IL6*, are often assigned small values of *g*_*j*_, which allows Spectra to attend to gene expression differences that occur on a smaller scale (Supplementary Fig. 4). By default, Spectra runs with at least one cell-type-specific factor per cell type so that global factors do not capture cell-type identities.

#### Determining cell-type granularity

Spectra can accommodate cell-type labels at any level of granularity, subject to a linear increase in computational burden with the number of cell types in the dataset. Additionally, as the granularity increases, the effective sample size for estimating cell-type-specific factors decreases, leading to potentially lower-quality cell-type-specific factors. The correct cell-type granularity depends on the dataset and the specific scientific questions at hand. First, the analyst should incorporate cell types that are known to be discrete and easily identifiable in the dataset via standard clustering analysis (for example, T cells, B cells, myeloid cells and epithelial cells). If cell subtypes exist that are not included as input to the model, Spectra devotes factors to describing variation across these subtypes. Moreover, if intermediate differentiation states between subtypes exist in the data, these subtypes should generally not be included as input to the model because (1) coarser cell-type-specific factors can describe these intermediate states, and (2) delineating between subtypes via clustering may be inaccurate.

### ${\boldsymbol{\mathcal{L}}}_{\mathbf{Graph}}$ (Θ): modeling gene–gene relationships in relation to expression data

In addition to faithful approximation of the input count matrix, we would also like interpretable factors that correspond known gene programs and biological processes (prior). Therefore, the second component of our likelihood function is a penalty term that guides the solution toward this prior. One aspect that makes Spectra unique is that it models this prior knowledge as a gene–gene community graph, which provides both computational efficiency and flexibility to adapt the graph structure to the data.

In this graph, nodes represent individual genes, and edges between genes occur when each gene has a similar distribution over factors. Communities within the graph, or densely connected subsets, then represent gene programs, whereas edges between communities contain information about genes that participate in multiple gene programs. Providing an imperfect, partially known graph structure as input, we can constrain our matrix factorization solution to respect the structure to yield interpretable gene programs. A main advantage of this approach is its flexibility. Gene sets are naturally incorporated into a graph by forming fully connected cliques among members of each set.

Further, more complex prior knowledge graph structures can be used as input, for example, arising from gene programs estimated from a separate dataset or cell atlas. Most importantly, the structure of this input gene–gene graph can be improved by fitting it to the data and learning gene programs that are more faithful to the data.

A second advantage of the graph prior is its scalability. Although gene sets may be highly overlapping, especially when curated from several separate databases, this redundancy is eliminated when storing information at the level of gene–gene relationships. Redundant gene sets will be merged into highly overlapping communities, and so two redundant gene sets can be approximately described by a single factor. A further computational advantage over gene set priors is that the dimensions of the graph are fixed as the size of gene set database increases, with only the number of edges increasing, and eliminates the need for iterating over the gene set dimension. Finally, operations involving the graph are implemented via efficient and parallelizable matrix multiplications with the graph adjacency matrix, thus allowing Spectra to efficiently scale to a large number of gene sets and cells (Fig. 2g).

To encourage factors to capture our prior knowledge of gene programs, we assume that binary gene–gene relationships are evidence of a pair of genes having similar latent profiles. This assumption could be incorporated by assuming a model for edge probabilities depending on the similarity scores 〈*θ*_*i*_, *θ*_*j*_〉 for genes *i* and *j*. However, the naive inner product does not explicitly account for the fact that prior information is invariably imperfect in systematic ways. First, at the level of entire gene programs, not all gene programs are active in all datasets, and, therefore, entire graph communities may be unnecessary for describing the observed expression data, while there are likely novel gene programs observed in the expression data that are not represented by communities in the graph. Also gene programs are imperfect due to inaccuracy of annotation, and, more frequently, gene programs differ across biological contexts, and our prior information is typically derived from a different biological context. Therefore, genes may be misclassified into gene sets to which they do not belong (corresponding to noisy edge observations), or gene sets may be incomplete (corresponding to missing edges). Spectra addresses these issues in the following two ways: (1) adaptively modeling background noise in the graph, allowing for the addition and removal of edges (Background edge rates), and (2) tuning the weight of the prior gene–gene matrix through the incorporation of a weight matrix, termed the factor interaction matrix, into the inner product between gene representations *θ*_*i*_ and *θ*_*j*_ (see below).

### The factor interaction matrix tunes the weight of the gene–gene prior

To understand the purpose of the factor interaction matrix, let us first consider the ordinary inner product measuring gene–gene similarity in terms of gene program representations:

$$\langle {\theta }_{i},{\theta }_{j}\rangle ={\theta }_{i1}{\theta }_{j1}+\cdots +{\theta }_{iK}{\theta }_{jK}$$

The maximum value of this product is 1 and is achieved only when gene *i* and gene *j* put all their weight into a single gene program. Consider what happens if genes *i* and *j* are important components of a gene program that exists only in the expression data and not in our prior information. Then, *i* and *j* are not connected in the graph, and so the inner product model encourages 〈*θ*_*i*_, *θ*_*j*_〉 ≈ 0. When 〈*θ*_*i*_, *θ*_*j*_〉 ≈ 0, genes *i* and *j* must be components of entirely separate programs. In this way, we see that the naive inner product discourages new factors from being estimated from the expression data. Such an inner product model estimates novel factors that are heavily biased by the graph.

Now, instead of the naive inner product, consider a weighted product weighted by scalar values (*b*_1_, *b*_2_,…, *b*_*K*_) that are between 0 and 1:

$${\langle {\theta }_{i},{\theta }_{j}\rangle }_{b}={b}_{1}{\theta }_{i1}{\theta }_{j1}+\cdots +{b}_{K}{\theta }_{iK}{\theta }_{jK}$$

To model the data, we can adjust the values of (*b*_1_,…, *b*_*K*_) to achieve the best fit. Consider the same situation as above, where *i* and *j* are not connected in the graph, but they are components of a gene program supported by expression data alone. The product model again encourages ${\langle {\theta }_{i},{\theta }_{j}\rangle }_{b}\approx 0$; however, now this constraint does not necessarily encourage *θ*_*i*_ and *θ*_*j*_ to be dissimilar. To see this, suppose that *θ*_*i*_ = [1, 0, 0] and *θ*_*j*_ = [1, 0, 0]. If *b*_1_ = 0, then

$$\begin{array}{rcl}{\langle {\theta }_{i},{\theta }_{j}\rangle }_{b}&=&{b}_{1}1* 1+{b}_{2}0* 0+{b}_{3}0* 0\\ &=&0\end{array}$$

Hence, novel gene programs can be estimated as long as the value of *b*_*k*_ corresponding to that program is pushed toward 0. We can interpret gene programs corresponding to low values of *b*_*k*_ as novel and gene programs corresponding to high values of *b*_*k*_ as supported by prior information. We could equivalently write each weight *b*_*k*_ as one of the non-zero elements of a diagonal matrix

$$B=\left[\begin{array}{ccc}{b}_{1}&&\\ &\ddots &\\ &&{b}_{K}\end{array}\right]$$

so that

$$\begin{array}{rcl}\langle {\theta }_{i},B{\theta }_{j}\rangle &=&{\langle {\theta }_{i},{\theta }_{j}\rangle }_{b}\\ &=&{b}_{1}{\theta }_{i1}{\theta }_{j1}+\cdots +{b}_{K}{\theta }_{iK}{\theta }_{jK}\end{array}$$

In practice, we allow the off diagonals of this matrix *B* to be estimated as non-zero (Supplementary Fig. 5). The resulting matrix is termed the factor interaction matrix.

Allowing off diagonals of the factor interaction matrix to be non-zero serves two purposes. First, it allows the model to explain overlapping gene sets without forcing shared genes to have partial membership. For example, if two gene sets overlap but in reality represent two distinct biological processes that can be separated in the gene expression data, the model is not forced to assign partial membership to overlapping genes but can fully assign genes to one of two programs. To account for this, the off-diagonal element corresponding to this pair of gene programs (*B*_*k*,*l*_ for programs *k* and *l*) can be estimated as greater than 0. On real data, we see this occur for β-alanine metabolism and fatty acid metabolism (Supplementary Fig. 6). Second, non-zero off-diagonal elements of the factor interaction matrix serve to mitigate the effect of low-quality edges in the prior graph by allowing edges between genes that are in separate gene expression programs to arise with non-zero probability.

### Full Spectra model

As a notation, we refer to the adjacency matrix of an input graph as $A\in {{\mathbb{R}}}^{p\times p}$ with element *A*_*ij*_ = 1 if an edge exists between *i* and *j* and *A*_*ij*_ = 0 otherwise. Following the discussion above, the Spectra generative model states (Supplementary Fig. 5)

$${\mathbb{P}}\left[{A}_{ij}=1\right]=\langle {\theta }_{i},B{\theta }_{j}\rangle$$

In the full Spectra model, each gene has a separate representation per cell type (in addition to its global representation), *θ*_*ci*_, where *c* indexes into the possible cell types. To supervise these representations in a cell-type-specific manner, the user (optionally) provides one graph for each cell type and a graph representing global gene–gene relationships (Supplementary Figs. 6 and 7). These graphs are modeled separately, where each graph’s edges can only be predicted using factor representations specific to that cell type. The cell-type-specific graphs are denoted *A*_*c*_ for cell type *c*, with *A*_*c*,*ij*_ = 1 if there is a cell-type-specific annotation between genes *i* and *j* for cell type *c*. The cell-type-specific graphs can only influence cell-type-specific factors and vice versa:

$${\mathbb{P}}\left[{A}_{c,ij}=1\right]=\langle {\theta }_{ci},{B}_{c}{\theta }_{cj}\rangle$$

diagrammed in Supplementary Fig. 7. Importantly, a separate factor interaction matrix, *B*_*c*_, is learned for each cell type with a prior graph provided.

The computational cost of including granular cell-type-specific prior information can be large, as each cell type requires its own graph.

### Background edge rates

Realistic annotation graphs have several edges that are not supported by expression data, and the model should be allowed the flexibility to attribute edges (or the lack thereof) in annotations to a background rate of noise. To allow flexibility in modifying the original graph, we incorporate background edge and non-edge rates *κ* and *ρ* that reflect noise rates in the observed graph. These parameters serve two separate purposes. First, these parameters deal with numerical stability issues by moving probabilities away from 0 and 1. Second, the parameters control the rate that edges are added and removed from the original graph. Intuitively, our inference procedure examines whether a relationship (or lack of a relationship) in the prior knowledge graph is consistent with expression data and if not can ascribe this relationship to random noise.

The generative process of our model is that with some probability *ρ*, edges between gene *i* and *j* are blocked out and cannot occur irrespective of the corresponding factor values *θ*_*i*_ and *θ*_*j*_. If this does not occur, an edge will be generated by random chance with probability *κ*. Finally, if neither of these events occur, an edge is generated according to the factor similarity score 〈*θ*_*i*_, *Bθ*_*j*_〉. This yields the following distribution for the adjacency matrix:

$$\begin{array}{l}{\mathbb{P}}\left[{A}_{ij}=1\right]=(1-\kappa )(1-\rho ){\theta }_{i}^{\top }B{\theta }_{j}+\kappa (1-\rho )\\ {\mathbb{P}}\left[{A}_{ij}=0\right]=(1-\kappa )(1-\rho )\left(1-{\theta }_{i}^{\top }B{\theta }_{j}\right)+\rho \end{array}$$

where *κ* and *ρ* are (cell-type-specific) background rates of 1 and 0 in the adjacency matrix, respectively. *κ* and *ρ* can be estimated from the data or fixed to constants and treated as tunable hyperparameters.

### Constructing the gene–gene prior graph

In most applications, Spectra receives a set of gene sets rather than a gene–gene graph as input, and the gene–gene graph is constructed from these gene sets. Large gene sets generally provide lower evidence that any given gene is crucial to the process that the gene set represents. For example, hallmark gene sets often contain hundreds of genes^76^, some of which are upregulated as distant downstream targets. Additionally, larger cliques represent a larger component of the likelihood function, potentially biasing Spectra solutions toward attending to the largest gene communities. Therefore, by default, when Spectra takes in gene sets as input, the edge weights used to downweight the contribution of any individual graph edge are proportional to the size of the gene set that it is derived from. The default weighting scheme is to weight edges by the total number of edges in the clique. For a given gene set *G*_*k*_, this involves downweighting by $\frac{1}{{{| {G}_{k}|} \choose {2}}}$:

$${w}_{ij}\propto \frac{1}{\left(\begin{array}{c}| {G}_{k}| \\ 2\end{array}\right)}$$

where ∣*G*_*k*_∣ is the size of a gene set *G*_*k*_ containing genes *i* and *j*. The weights are rescaled so that the median weight across gene sets is 1. When a pair of genes exists in multiple gene sets, the weights accumulate additively. Another reasonable choice is ${w}_{ij}=\frac{1}{\max [d(i),d(\,j)]}$, where *d*(*i*) is the degree of node *i*.

As an alternative weighting scheme, Spectra accommodates weighted graphs by scaling edges by edge-specific weights. This feature allows users to annotate the prior information graphs with additional quantitative information representing relative confidence in each individual annotation.

### Pseudolikelihood function

The heretofore described model components describe the expected values of the expression data matrix **X** and the prior knowledge graph *A* under the Spectra generative process. Together with specific observation distributions, this would specify a likelihood function that serves as the maximization objective of Spectra, fit via either first-order methods or expectation maximization (EM). The loss function described below is the negative value of a proper likelihood function in the case where weights *w*_*ij*_ are equal to 1, *λ* = 1, and expression data **X** follow a Poisson distribution. In practice, these conditions are not satisfied, so we adopt the terminology pseudolikelihood function to describe the negative loss function. For ease of exposition, we first describe the pseudolikelihood function assuming a single cell type. Recall the general form of the Spectra objective, consisting of a term that measures the ability of Spectra factors to recapitulate expression data and a term that measures the concordance of Spectra factors with the prior knowledge database:

$${{{\mathcal{L}}}}(\Theta )=\lambda {{{{\mathcal{L}}}}}_{{{{\rm{Reconstruction}}}}}(\Theta )+{{{{\mathcal{L}}}}}_{{{{\rm{Graph}}}}}(\Theta )$$

As edges are binary, combined with the assumption of independence, the log likelihood of *A*_*ij*_ given a probability of 1, ${p}_{ij}:= {\mathbb{P}}[{A}_{ij}=1]$, is

$$\log {\mathbb{P}}({A}_{ij})={A}_{ij}\log {p}_{ij}+(1-{A}_{ij})\log (1-{p}_{ij})$$

With *p*_*ij*_ as described in Modeling gene–gene relationships in relation to expression data and The factor interaction matrix tunes the weight of the gene–gene prior,

$$\begin{array}{l}\log {\mathbb{P}}({A}_{ij})={A}_{ij}\log \left[(1-\kappa )(1-\rho ){\theta }_{i}^{\top }B{\theta }_{j}+\kappa (1-\rho )\right]\\ \qquad\qquad\quad\;+(1-{A}_{ij})\log \left[(1-\kappa )(1-\rho )(1-{\theta }_{i}^{\top }B{\theta }_{j})+\rho \right]\end{array}$$

To incorporate weights (following Constructing the gene–gene prior graph), we weight likelihood terms corresponding to each edge in the graph by an edge-specific weight *w*_*ij*_:

$$\begin{array}{rcl}\log {\mathbb{P}}({A}_{ij})&=&{w}_{ij}{A}_{ij}\log \left[(1-\kappa )(1-\rho ){\theta }_{i}^{\top }B{\theta }_{j}+\kappa (1-\rho )\right]\\ &+&(1-{A}_{ij})\log \left[(1-\kappa )(1-\rho )(1-{\theta }_{i}^{\top }B{\theta }_{j})+\rho \right]\end{array}$$

Combining across all observations (*i*, *j*), this leads to the expression for ${{{{\mathcal{L}}}}}_{{{{\rm{Graph}}}}}(\Theta )$:

$$\begin{array}{rcl}{{{{\mathcal{L}}}}}_{{{{\rm{Graph}}}}}(\Theta )&=&\mathop{\sum }\limits_{i=1}^{p}\,\mathop{\sum }\limits_{j=1,j\ne i}^{p}\left[{w}_{ij}{A}_{ij}\log \left((1-\kappa )(1-\rho ){\theta }_{i}^{\top }B{\theta }_{j}+\kappa (1-\rho )\right)\right.\\ &+&\left.(1-{A}_{ij})\log \left((1-\kappa )(1-\rho )(1-{\theta }_{i}^{\top }B{\theta }_{j})+\rho \right)\right]\end{array}$$

The loss function derived from the Poisson distribution has been widely used for modeling scRNA-seq counts^4,56,77^. Although processed data may not necessarily be well described by the Poisson observation model (that is, scran-processed data are on a log scale), the resulting log likelihood strikes a practical balance in scaling with gene expression magnitude. The resulting loss function has been used in contexts other than modeling count data as the KL divergence loss^78^. Additionally, under idealized settings, estimates obtained by minimizing this loss function inherit properties of M estimators analogous to those of maximum likelihood estimators, with few assumptions on the data distribution^79^. Here, we are primarily concerned with how the loss function behaves under changes in scale. For example, suppose we have an estimated expression value ${\hat{{{{\bf{X}}}}}}_{ij}$. We can write ${\hat{{{{\bf{X}}}}}}_{ij}(\Theta )$ as our predicted gene expression as a function of the model parameters. The least squares loss ${{{{\mathcal{L}}}}}_{2}(\Theta ):= {[{{{{\bf{X}}}}}_{ij}-{\hat{{{{\bf{X}}}}}}_{ij}(\Theta )]}^{2}$ is quadratically dependent on the scale of **X**_*ij*_, because replacing both ground truth and estimate by scaled versions *φ***X**_*ij*_ and $\varphi {\hat{{{{\bf{X}}}}}}_{ij}$ leads to a loss of ${\varphi }^{2}{{{{\mathcal{L}}}}}_{2}(\Theta )$. Similar to the issues addressed in ‘L_Reconstruction_ (Θ): modeling gene expression as a low-rank product’, the squared loss function encourages factors to attend to highly expressed genes because scale differences amplify the loss quadratically. At the other extreme, consider the Itakura–Saito loss (IS loss) given by (we briefly assume that both ground truth and estimate are not 0)

$${{{{\mathcal{L}}}}}_{IS}(\Theta ):= \frac{{{{{\bf{X}}}}}_{ij}}{{\hat{{{{\bf{X}}}}}}_{ij}(\Theta )}-\log \frac{{{{{\bf{X}}}}}_{ij}}{{\hat{{{{\bf{X}}}}}}_{ij}(\Theta )}-1$$

If we scale observed counts and prediction *φ***X**_*ij*_ and $\varphi {\hat{{{{\bf{X}}}}}}_{ij}$, then the IS loss does not change. So, matrix factorization with the IS loss does not suffer from a bias toward highly expressed genes. However, forcing the model to predict all lowly expressed genes is not desirable, often leading to low-quality factors. The Poisson log likelihood exhibits a practically convenient balance between these two extremes:

$${{{{\mathcal{L}}}}}_{{{{\rm{Pois}}}}}(\Theta ):= -{{{{\bf{X}}}}}_{ij}\log {\hat{{{{\bf{X}}}}}}_{ij}(\Theta )+{\hat{{{{\bf{X}}}}}}_{ij}(\Theta )$$

When **X**_*ij*_ and ${\hat{{{{\bf{X}}}}}}_{ij}$ are scaled by *φ*, ${{{{\mathcal{L}}}}}_{{{{\rm{Pois}}}}}(\Theta )$ is scaled by *φ*. This linear dependence on expression scale achieves a good balance in the relative weighting between highly expressed and lowly expressed genes.

An additional advantage of this loss function is that the second term behaves as a lasso penalty^80^, inducing sparsity in the resulting estimates of ${\hat{{{{\bf{X}}}}}}_{ij}$ for sparse data **X**, noted by Gopalan et al.^81^. This sparsity allows for a parsimonious explanation of a cell’s gene expression using as few factors as possible. In Spectra, we have ${\hat{{{{\bf{X}}}}}}_{ij}:= {\alpha }_{i}^{\top }{\theta }_{j}({g}_{j}+\delta )$, yielding the expression

$${{{{\mathcal{L}}}}}_{{{{\rm{Reconstruction}}}}}(\Theta )=\mathop{\sum }\limits_{i=1}^{n}\mathop{\sum }\limits_{j=1}^{p}{{{{\bf{X}}}}}_{ij}\log \left[{\alpha }_{i}^{\top }{\theta }_{j}({g}_{j}+\delta )\right]-{\alpha }_{i}^{\top }{\theta }_{j}({g}_{j}+\delta )$$

Combining the components, the pseudo-log likelihood function is

$$\begin{array}{rcl} {\mathcal{L}}(\alpha, \theta, g, B) &=& \lambda \underbrace{\mathop{\sum}\limits_{i=1}^n\mathop{\sum}\limits_{j=1}^p {\mathbf{X}}_{ij}\log\left(\alpha_{i}^\top \theta_j (g_j + \delta)\right) - \alpha_{i}^\top\theta_j(g_j + \delta)}_{{\mathcal{L}}_{{\rm{Reconstruction}}}} \\ & +& \mathop{\sum}\limits_{i=1}^p\,\mathop{\sum}\limits_{j=1,\, j\neq i}^p\left[w_{ij}A_{ij}\log\left((1-\kappa)(1-\rho)\theta_{i}^\top B \theta_j + \kappa(1-\rho) \right)\right. \\ & +& \underbrace{\left.(1-A_{ij})\log\left((1-\kappa)(1-\rho)(1-\theta_{i}^\top B \theta_j) + \rho\right)\right]}_{{{\mathcal{L}}_{{\rm{Graph}}}}} \end{array}$$

Again, **X** is the data matrix after processing, whereas *α*, *θ*, *g* and *B* are the four model parameters that need to be estimated. The first term in the pseudo-log likelihood function comes from the log likelihood of the Poisson distribution (also referred to as the KL divergence loss function when multiplied by −1), while the second term is the log likelihood of a Bernoulli distribution with positive observations scaled by *w*_*ij*_. The pseudolikelihood function optimized by Spectra includes an optimization over cell-type-specific and global parameters, and so an additional sum over cell types is included in the pseudo-log likelihood (Supplementary Fig. 7).

$$\begin{array}{rcl} {\mathcal{L}}(\alpha, \theta, g, B) &=&\mathop{\sum}\limits_{c=1}^C\mathop{\sum}\limits_{i=1}^{n_c} \lambda_c\mathop{\sum}\limits_{j=1}^p {\mathbf{X}}_{cij}\log\left((g_j + \delta)\alpha_{c,i,:K}^\top \theta_j + (g_{cj} + \delta) \alpha_{c,i, K+1:}^\top \theta_{cj}\right)\\ && \underbrace{- \mathop{\sum}\limits_{c=1}^C\mathop{\sum}\limits_{i=1}^{n_c}\mathop{\sum}\limits_{j=1}^p\left((g_j + \delta)\alpha_{c,i,:K}^\top \theta_j + (g_{cj} + \delta) \alpha_{c,i, K+1:}^\top \theta_{cj}\right)}_{{\mathcal{L}}_{{\rm{Reconstruction}}}}\\ && + \mathop{\sum}\limits_{c=1}^{C+1} \mathop{\sum}\limits_{i=1}^p\,\mathop{\sum}\limits_{j=1,\, j\neq i}^p\left[w_{c,ij}A_{c,ij}\log\left((1-\kappa_c)(1-\rho_c)\theta_{ci}^\top B_c \theta_{cj} + \kappa_c(1-\rho_c)\right)\right.\\ && \underbrace{+\left.(1-A_{c,ij})\log\left((1-\kappa_c)(1-\rho_c)(1-\theta_{ci}^\top B_c \theta_{cj}) + \rho_c\right)\right]}_{{{\mathcal{L}}_{{\rm{Graph}}}}} \end{array}$$

As all discrete parameters have been integrated out, this pseudo-log likelihood can be directly maximized via first-order methods, such as gradient descent. Approximate second-order methods are not ideal due to the high dimension of the parameter space for practical problem sizes. However, for smaller-sized problems (in terms of number of genes and factors), we develop an EM approach that yields intuitive coordinate ascent updates of model parameters.

### Spectra’s output

To describe the activity level of factor *k* in cell *i*, we compute cell scores as cell_score_*ik*_ = *q*_*k*_*α*_*ik*_, where ${q}_{k}=\frac{1}{p}\mathop{\sum }\nolimits_{j = 1}^{p}{\theta }_{jk}$. In other words, the cell scores are the loadings weighted by the total factor usage across all genes. This allows us to circumvent the non-identifiability of scale associated with factor analysis approaches. Regarding terminology, we will always refer to the unnormalized loadings *α*_*ik*_ as ‘loadings’ and the normalized loadings as cell scores. Additionally the cell-specific parameters of other matrix factorization methods are described as ‘loadings’. The ground truth parameters in our simulations are also described as ‘loadings’.

To describe the relevance of gene *j* for factor *k*, we compute gene scores for gene *j* and factor *k* as $\left(\frac{{g}_{j}+\delta }{{g}_{j}+\delta +{\mathtt{offset}}}\right){\theta }_{jk}$. The first term is near 0 when *g*_*j*_ is very small and near 1 when *g*_*j*_ is large. This allows us to remove very lowly expressed genes from the factors while maintaining coherence. By default, the offset term is set to 1, can be tuned and in some cases set to 0, which yields the factors *θ*_*jk*_ themselves. Each *θ*_*jk*_ is more directly influenced by the prior than *g*_*j*_*θ*_*jk*_, and so setting offset to 0 tends to yield marker lists closely resembling input gene sets.

Users can access additional parameters that facilitate interpretation of the gene scores and cell scores. The factor interaction matrix per cell type (*B*; Supplementary Fig. 6) contains entries in the range [0, 1], where diagonal entries can be interpreted as the relevance of a given factor to the prior graph. Off-diagonal entries can be interpreted as a background rate of edges between genes that are expressed in separate factors. For each cell type, users can access a posterior graph that is denoised using information from the expression data. The posterior graph is computed by the inner product 〈*θ*_*i*_, *Bθ*_*j*_〉 for each pair of genes *θ*_*i*_ and *θ*_*j*_ after estimating *θ*_*i*_, *θ*_*j*_ and *B* from the data.

Of importance are the diagonal elements of the interaction matrices *B*, which contain information about the dependence of the factor on the input graph. We term these diagonal elements *η*, specifically *η*_*c*_ ≔ diag(*B*_*c*_).

### Factor importance and information scores

We adopt the following two metrics to prioritize factors in the output of Spectra: factor importance and factor information scores, each measuring a different property of the factor. Both metrics are computed per cell type for all of the factors that are potentially relevant to that cell type. In other words, to prioritize the relevant factors for a cell type, the metrics are computed for each cell-type-specific factor and each global factor, resulting in 2(*K* + *K*_*c*_) scores for cell type *c*. The factor importance score measures the overall contribution of a factor to explain the observed expression data (as measured by the reconstruction component of the loss function), regardless of whether this factor explains within-cell-type variation. The factor information score, complementary to the factor importance score, measures whether the gene set associated with a factor captures meaningful within-cell-type variation. Factors with high scores in either of these categories are potentially of interest for post hoc analysis. The factor importance score is a relative change in reconstruction error for a specific cell type when a certain factor is masked out. Let ${\bar{L}}_{c}(\theta ,{\theta }_{j})$ be the reconstruction error for cell type *c*:

1 $${\bar{L}}_{c}(\theta ,{\theta }_{j}):= \mathop{\sum }\limits_{i=1}^{{n}_{c}}{\lambda }_{c}\mathop{\sum }\limits_{j=1}^{p}{{{{\bf{X}}}}}_{cij}\log \left[({g}_{j}+\delta ){\alpha }_{c,i,:K}^{\top }{\theta }_{j}+({g}_{cj}+\delta ){\alpha }_{c,i,K+1:}^{\top }{\theta }_{cj}\right]$$

2 $$-\mathop{\sum }\limits_{i=1}^{{n}_{c}}\mathop{\sum }\limits_{j=1}^{p}\left[({g}_{j}+\delta ){\alpha }_{c,i,:K}^{\top }{\theta }_{j}+({g}_{cj}+\delta ){\alpha }_{c,i,K+1:}^{\top }{\theta }_{cj}\right]$$

Here, it is understood that all parameters except *θ* and *θ*_*j*_ are fixed to their fitted values. Further, let *ϵ*_*k*_ denote a vector of all 1 values of a dimension equal to the number of factors, except at *k* where it is 0: *ϵ*_*k*_ = 1 − *e*_*k*_. The importance score for cell-type-specific factor *k* is then ${{{{\mathcal{F}}}}}_{c,k}=\frac{{\bar{L}}_{c}(\theta ,{\epsilon }_{k}\circ {\theta }_{j})-{\bar{L}}_{c}(\theta ,{\theta }_{j})}{{\bar{L}}_{c}(\theta ,{\theta }_{j})}$, where ° represents elementwise product. Similarly the importance score for a global factor *k* is given by ${{{{\mathcal{F}}}}}_{c,k}^{(g)}=\frac{{\bar{L}}_{c}({\epsilon }_{k}\circ \theta ,{\theta }_{j})-{\bar{L}}_{c}(\theta ,{\theta }_{j})}{{\bar{L}}_{c}(\theta ,{\theta }_{j})}$

Information scores are given by Definition (1) in Mimno et al.^82^ but computed per cell type to represent cell-type-specific information content. Specifically, given a marker list associated with a factor and with *M* set to 30 we have

3 $${C}_{c,k}=\mathop{\sum }\limits_{m=2}^{M}\mathop{\sum }\limits_{l=1}^{m-1}\log \frac{{D}_{c}({g}_{m}^{(k)},{g}_{l}^{(k)})+1}{{D}_{c}({g}_{l}^{(k)})}$$

where ${g}_{m}^{(k)}$ is now the *m*th top gene for factor *k*, and *D*_*c*_(⋅, ⋅) and *D*_*c*_(⋅) are the co-occurrence frequency and frequency, respectively, within cell type *c*. We plot $\exp ({C}_{c,k})$ as the information scores.

### Optimization

We develop the following two optimization schemes: an auxiliary latent variable EM approach and gradient descent-based optimization via Adam^83^. EM converges quickly in many situations; however, the memory requirements are substantially larger than the gradient descent-based optimization. Specifically the memory requirement of EM parameter storage is *O*(*npK* + *p*^2^*K*^2^) due to auxiliary parameter storage, while the memory requirement of gradient descent is substantially lower, *O*(*nK* + *pK* + *K*^2^).

Although memory intensive, the EM solution is valuable for two reasons: (1) for problems with a small number of factors (<20) and genes (<2,500), EM is fast and less sensitive to initialization than gradient descent^84^, and (2) the EM updates are intuitive and give us an understanding of how our algorithm balances evidence from the graph and expression data.

However, optimization with Adam can handle a large number of factors (>200) and genes (>10,000) and can exhibit stability with the appropriate initialization. By default, Spectra uses Adam for optimization.

### EM

For ease of exposition, we describe the EM routine for the non-integrative model; the updates are easily extendable to incorporate cell-type labels. Additionally, we write the pseudolikelihood function equivalently (up to a scale factor) in terms of $\tilde{\lambda }:= \frac{1}{\lambda }$. To make EM possible, we exploit two facts about the distribution of (**X**, **A**)^85^.

The first is that if *z*_*ijk*_ ~ Pois[(*g*_*j*_ + *δ*)*α*_*ik*_*θ*_*jk*_] and we define ${{{{\bf{X}}}}}_{ij}=\mathop{\sum }\nolimits_{k = 1}^{K}{z}_{ijk}$, then **X**_*ij*_ still has the correct marginal distribution due to standard properties of the Poisson distribution^81^. Second, if we define ${\tilde{z}}_{ij} \sim {{{\rm{Categorical}}}}({\theta }_{i})$ and ${\tilde{z}}_{ji} \sim {{{\rm{Categorical}}}}({\theta }_{j})$ and define a conditional distribution for *A*_*ij*_ as

$${\mathbb{P}}\left({{{{\bf{A}}}}}_{ij}=1| {\tilde{z}}_{ijk}=1,{\tilde{z}}_{jil}=1\right)={B}_{kl}$$

then **A**_*ij*_ still has the correct marginal distribution^86^. As a result, we can optimize the marginal log likelihood via optimization of the expected complete data log likelihood ${{\mathbb{E}}}_{z,\tilde{z}}[\log p({{{\bf{X}}}},{{{\bf{A}}}},z,\tilde{z})]$, where the expectation is taken over the posterior $p(z,\tilde{z}| {{{\bf{A}}}},{{{\bf{X}}}})$. The expected complete data log likelihood is given by

$$\begin{array}{rcl}\tilde{{{{\mathcal{L}}}}}(\alpha ,B,\theta ,g)&=&\mathop{\sum }\limits_{i=1}^{n}\,\mathop{\sum }\limits_{j=1}^{p}\mathop{\sum }\limits_{k=1}^{K}{\phi }_{ijk}\log (({g}_{j}+\delta ){\alpha }_{ik}{\theta }_{jk})-({g}_{j}+\delta ){\alpha }_{ik}{\theta }_{jk}\\ &&+\;\tilde{\lambda }\mathop{\sum }\limits_{i=1}^{p}\,\mathop{\sum }\limits_{j=1,\,j\ne i}^{p}\mathop{\sum }\limits_{k=1}^{K}\mathop{\sum }\limits_{l=1}^{K}{\tilde{\phi }}_{ijkl}\left({w}_{ij}{A}_{ij}\log \left((1-\kappa ){B}_{kl}+\kappa \right)\right.\\ &&+\left.(1-{A}_{ij})\log \left((1-\kappa )(1-\rho )(1-{B}_{kl})+\rho \right)+\log {\theta }_{ik}+\log {\theta }_{jl}\right)\end{array}$$

where $${\phi }_{ijk}:= {\mathbb{E}}({z}_{ijk}| {{{\bf{X}}}})={{{{\bf{X}}}}}_{ij}\frac{{\alpha }_{ik}{\theta }_{jk}}{\mathop{\sum }\nolimits_{k = 1}^{K}{\alpha }_{ik}{\theta }_{jk}}$$ and

$$\begin{array}{rcl}{\tilde{\phi }}_{ijkl}&=&{\mathbb{P}}\left({\tilde{z}}_{ijk}=1,{\tilde{z}}_{jil}=1| {{{\bf{A}}}}\right)\\ &\propto &{\theta }_{ik}{\theta }_{jl}{\left((1-\kappa ){B}_{kl}+\kappa \right)}^{{w}_{ij}{A}_{ij}}{\left((1-\kappa )(1-\rho )(1-{B}_{kl})+\rho \right)}^{1-{A}_{ij}}\end{array}$$

Importantly, this manipulation moves summations outside of the logs, which permits analytic EM updates for *B*, *α* and *g* given by

$$\begin{array}{l}{\alpha }_{ik}\leftarrow \frac{\mathop{\sum }\nolimits_{j = 1}^{p}{\phi }_{ijk}}{\mathop{\sum }\nolimits_{j = 1}^{p}{\theta }_{jk}({g}_{j}+\delta )}\\ {g}_{j}\leftarrow {{{{\rm{proj}}}}}_{[0,1]}\left(\frac{\mathop{\sum }\nolimits_{i = 1}^{n}\mathop{\sum }\nolimits_{k = 1}^{K}{\phi }_{ijk}}{\mathop{\sum }\nolimits_{i = 1}^{n}\mathop{\sum }\nolimits_{k = 1}^{K}{\alpha }_{ik}{\theta }_{jk}}-\delta \right)\\ {B}_{kl}\leftarrow {{{{\rm{proj}}}}}_{[0,1]}\left(\frac{\left(\frac{\rho }{1-\rho }+(1-\kappa )\right){\Xi }_{kl}-\kappa }{(1-\kappa )(1+{\Xi }_{kl})}\right)\end{array}$$

where ${\Xi }_{kl}:= \frac{\mathop{\sum }\nolimits_{i = 1}^{p}\mathop{\sum }\nolimits_{j = 1}^{p}{\tilde{\phi }}_{ijkl}{w}_{ij}{A}_{ij}}{\mathop{\sum }\nolimits_{i = 1}^{p}\mathop{\sum }\nolimits_{j = 1}^{p}{\tilde{\phi }}_{ijkl}(1-{A}_{ij})}$, representing an odds ratio between Bernoulli outcomes. Further, the complete data log likelihood has diagonal Hessian when viewed as a function of *θ* only, $\tilde{L}(\theta )$, permitting linear time Newton Raphson updates

$$\begin{array}{l}{\gamma }_{jl}\leftarrow \frac{1}{{\theta }_{jl}}\left[\mathop{\sum }\limits_{i=1}^{n}\mathop{\sum }\limits_{j=1}^{p}{\phi }_{ijl}+\tilde{\lambda }\mathop{\sum }\limits_{i=1}^{p}\mathop{\sum }\limits_{k=1}^{K}{\tilde{\phi }}_{ijkl}+\tilde{\lambda }\mathop{\sum }\limits_{i=1}^{p}\mathop{\sum }\limits_{k=1}^{K}{\tilde{\phi }}_{jilk}\right]-({g}_{j}+\delta )\mathop{\sum }\limits_{i=1}^{n}{\alpha }_{il}\\ {H}_{jl}^{-1}\leftarrow \frac{-{\theta }_{jl}^{2}}{\mathop{\sum }\nolimits_{i = 1}^{n}\mathop{\sum }\nolimits_{j = 1}^{p}{\phi }_{ijl}+\tilde{\lambda }\mathop{\sum }\nolimits_{i = 1}^{p}\mathop{\sum }\nolimits_{k = 1}^{K}{\tilde{\phi }}_{ijkl}+\tilde{\lambda }\mathop{\sum }\nolimits_{i = 1}^{p}\mathop{\sum }\nolimits_{k = 1}^{K}{\tilde{\phi }}_{jilk}}\\ {\Delta }_{j}\leftarrow -\frac{\mathop{\sum }\nolimits_{k = 1}^{K}{\gamma }_{jk}{H}_{jk}^{-1}}{\mathop{\sum }\nolimits_{k = 1}^{K}{H}_{jk}^{-1}}\\ {\theta }_{jk}\leftarrow {\theta }_{jk}-{H}_{jk}^{-1}({\Delta }_{j}+{\gamma }_{jk})\end{array}$$

**Algorithm 1.** EM Spectra routine

**Require:** $${{{\bf{X}}}}\ge 0,{{{\bf{A}}}}\in {\{0,1\}}^{p\times p},T\in {{\mathbb{Z}}}_{+},\kappa \in {{\mathbb{R}}}_{+},\rho \in {{\mathbb{R}}}_{+},\tilde{\lambda }\in {{\mathbb{R}}}_{+}$$

 initialize *B*, *α*, *θ*, *g*

 **while** $${{{{\mathcal{L}}}}}_{n}-{{{{\mathcal{L}}}}}_{n-1} > \epsilon$$, **do**

  $${\phi }_{ijk}\leftarrow {{{{\bf{X}}}}}_{ij}\frac{{\alpha }_{ik}{\theta }_{jk}}{\mathop{\sum }\nolimits_{k = 1}^{K}{\alpha }_{ik}{\theta }_{jk}}$$

  $${\tilde{\phi }}_{ijkl}\leftarrow {\theta }_{ik}{\theta }_{jl}{((1-\kappa ){B}_{kl}+\kappa )}^{{w}_{ij}{A}_{ij}}{((1-\kappa )(1-\rho )(1-{B}_{kl})+\rho )}^{1-{A}_{ij}}$$

  $${\tilde{\phi }}_{ijkl}\leftarrow {\tilde{\phi }}_{ijkl}/{\sum }_{kl}{\tilde{\phi }}_{ijkl}$$

  **while** *t* < *T*, **do**

   $${\gamma }_{jl}\leftarrow \frac{1}{{\theta }_{jl}}\left[\mathop{\sum }\nolimits_{i = 1}^{n}\mathop{\sum }\nolimits_{j = 1}^{p}{\phi }_{ijl}+\tilde{\lambda }\mathop{\sum }\nolimits_{i = 1}^{p}\mathop{\sum }\nolimits_{k = 1}^{K}{\tilde{\phi }}_{ijkl}+\tilde{\lambda }\mathop{\sum }\nolimits_{i = 1}^{p}\mathop{\sum }\nolimits_{k = 1}^{K}{\tilde{\phi }}_{jilk}\right]$$$$-({g}_{j}+\delta )\mathop{\sum }\nolimits_{i = 1}^{n}{\alpha }_{il}$$

   $${H}_{jl}^{-1}\leftarrow \frac{-{\theta }_{jl}^{2}}{\mathop{\sum }\nolimits_{i = 1}^{n}\mathop{\sum }\nolimits_{j = 1}^{p}{\phi }_{ijl}+\tilde{\lambda }\mathop{\sum }\nolimits_{i = 1}^{p}\mathop{\sum }\nolimits_{k = 1}^{K}{\tilde{\phi }}_{ijkl}+\tilde{\lambda }\mathop{\sum }\nolimits_{i = 1}^{p}\mathop{\sum }\nolimits_{k = 1}^{K}{\tilde{\phi }}_{jilk}}$$

   $${\Delta }_{j}\leftarrow -\frac{\mathop{\sum }\nolimits_{k = 1}^{K}{\gamma }_{jk}{H}_{jk}^{-1}}{\mathop{\sum }\nolimits_{k = 1}^{K}{H}_{jk}^{-1}}$$

   $${\theta }_{jk}\leftarrow {\theta }_{jk}-{H}_{jk}^{-1}({\Delta }_{j}+{\gamma }_{jk})$$

  $${\alpha }_{ik}\leftarrow \frac{\mathop{\sum }\nolimits_{j = 1}^{p}{\phi }_{ijk}}{\mathop{\sum }\nolimits_{j = 1}^{p}{\theta }_{jk}({g}_{j}+\delta )}$$

  $${g}_{j}\leftarrow {{{{\rm{proj}}}}}_{[0,1]}\left(\frac{\mathop{\sum }\nolimits_{i = 1}^{n}\mathop{\sum }\nolimits_{k = 1}^{K}{\phi }_{ijk}}{\mathop{\sum }\nolimits_{i = 1}^{n}\mathop{\sum }\nolimits_{k = 1}^{K}{\alpha }_{ik}{\theta }_{jk}}-\delta \right)$$

  $${B}_{kl}\leftarrow {{{{\rm{proj}}}}}_{[0,1]}\left(\frac{(\frac{\rho }{1-\rho }+(1-\kappa )){\Xi }_{kl}-\kappa }{(1-\kappa )(1+{\Xi }_{kl})}\right)$$

  $${{{{\mathcal{L}}}}}_{n}\leftarrow {{{\mathcal{L}}}}(\alpha ,\theta ,B,g)$$

The integrative version of Spectra uses analogous updates, with the bounds of summations appropriately modified; specifically, the E step updates are ${\phi }_{ijk}={{{{\bf{X}}}}}_{ij}\frac{{g}_{c,\,j}{\alpha }_{ik}{\theta }_{jk}}{{g}_{j}\mathop{\sum }\nolimits_{k = 1}^{K}{\alpha }_{ik}{\theta }_{jk}+{g}_{c,\,j}\mathop{\sum }\nolimits_{k = K+1}^{K+{K}_{c}}{\alpha }_{ik}{\theta }_{jk}}$ for cell-type-specific factors and ${\phi }_{ijk}={{{{\bf{X}}}}}_{ij}\frac{{g}_{j}{\alpha }_{ik}{\theta }_{jk}}{{g}_{j}\mathop{\sum }\nolimits_{k = 1}^{K}{\alpha }_{ik}{\theta }_{jk}+{g}_{c,\,j}\mathop{\sum }\nolimits_{k = K+1}^{K+{K}_{c}}{\alpha }_{ik}{\theta }_{jk}}$ for global factors.

### Adam

For large scRNA-seq datasets, the memory requirement of EM for fitting a large number of factors is prohibitive. We optimize the marginal log likelihood with Adam^83^, a momentum-based gradient descent optimizer implemented in pytorch, directly. In detail, the Adam hyperparameters *β*_1_ and *β*_2_ are set to default values 0.9 and 0.999, respectively. We use a learning rate schedule of [1.0, 0.5, 0.1, 0.01, 0.001, 0.0001], where training at subsequent learning rates occurs after convergence at higher learning rates. A maximum number of iterations is fixed to 10,000. This default training scheme can be modified by the user. In particular, for faster convergence, either the maximum number of iterations can be made smaller or the smallest learning rates can be removed, allowing for solutions that are not as fine tuned.

### Initialization

Because the Spectra objective function is non-convex and susceptible to suboptimal local maxima, initialization plays an important role in the quality of the eventual solutions. When Spectra is provided with gene sets as input, our strategy is to initialize factors as close to the gene sets as possible. Whenever the number of factors is greater than the number of gene sets, we resort to a gene set-based initialization procedure.

First, a hyperparameter *t* controls the strength of the initialization. By default, *t* is set to 25. For a given cell type, whenever the number of factors is at least as large as the number of gene sets, we initialize $\log {\theta }_{ij}\leftarrow t$ when gene *i* belongs to gene set *j* for each gene set *j* = 1,…, *N*_gs_, and *N*_gs_ is the number of gene sets. Further, the factor interaction matrix is initialized with logit*B*_*jj*_ ← *t* to encode the knowledge that this factor corresponds to a gene set. To encourage the last factor to capture genes that have no edges in the prior graph, we initialize the last row and column to small values, logit *B*_*K*,*j*_ ← −*t* and logit *B*_*j*,*K*_ ← −*t* for all *j* = 1,…, *K*. Corollary 1 (Supplementary Note) explains why this leads to extremely fast convergence when *λ* is small.

For a given cell type, when the number of factors is not greater than the number of gene sets, we resort to initialization with NMF^87^.

### GPU acceleration

For all results involving GPU acceleration, a wrapper around the original model implementation is provided, loading models onto the GPU via the pytorch syntax device = torch.device (‘cuda:0’) and model.to (device) when CUDA is available. Data (including adjacency matrices and expression data) are similarly loaded onto GPU. All GPU methods were run on an NVIDIA A100 Tensor Core GPU.

### Determining the number of factors

We adopt two approaches to determine the number of factors. The first approach is to set the number of factors for each cell type equal to the number of gene sets available for that cell type + 1 (similar to the approach taken by Slalom), and the second approach is to estimate the number of factors from the data via bulk eigenvalue matching analysis^88^. Fitting a large number of factors is possible; in our experiments, we fit a set of 197 factors.

The second approach involves three steps. In the first step, we estimate a null distribution of eigenvalues based on sampling variances ${\sigma }_{j}^{2} \sim$ Gamma(*θ*, 1/*θ*) and then subsequently an *n* × *p* Gaussian matrix ${Z}_{ij} \sim N(0,{\sigma }_{j}^{2})$. We sample *B* of these matrices and take the average of the sorted eigenvalues of $\frac{1}{n}Z{Z}^{\,t}$ over *B* samples. Typically *B* is set to 100. Given the average sorted eigenvalues, we compute a regression coefficient without intercept between the ‘bulk’ of these eigenvalues and the bulk of the observed eigenvalues of the data covariance matrix. The bulk of the distribution are the values between some lower and upper quantiles, which are hyperparameters of the method. We perform a line search on *θ* to find a value of *θ* that minimizes the sum of squared residuals of this regression. Denoting this regression coefficient as *β*, in the second step, we simulate a background distribution based on sampling variance terms ${\sigma }_{j}^{2} \sim {{{\rm{Gamma}}}}(\theta ,\beta /\theta )$, data from ${Z}_{ij} \sim N(0,{\sigma }_{j}^{2})$ and eigenvalues from $\frac{1}{n}Z{Z}^{\,t}$. Finally, *K* is estimated as the (1 − *α*) quantile of the simulated distribution of leading eigenvalues. We apply this process for every cell type separately.

### Determining Spectra’s input parameters

We summarize all user-defined inputs to the Spectra algorithm.

**X**: Expression matrix with *n* cells and *p* genes (required).

*λ*: Regularization strength of prior graph (required).

Gene set dictionary: dictionary with cell types as keys and gene sets as values (optional).

Cell-type labels: list of cell types corresponding to expression matrix (optional).

*δ*: parameter that bounds minimum gene scale factor (optional).

**w**: graph edge weights (optional).

*κ*: background rate of edges (optional).

*ρ*: background rate of edge deletion.

The data matrix and regularization strength *λ* must be provided by the user, while prior information can be provided in the form of a dictionary of cell-type-specific and global gene sets (note that Spectra can also be run by providing graph adjacency matrices directly). Optionally, cell-type labels that align with keys of the gene set dictionary can be provided. The lower bound for gene scale factors, *δ*, controls the extent that gene expression is normalized and is set to a default value of 0.001. This translates to a maximum ratio of gene scale factors of ≈1,000. By default, the graph edge weights are set to be inversely proportional to the total number of edges induced by the gene set leading to a given edge and accumulate additively for genes in multiple sets. The background rate of noise edges, *κ*, and the rate at which edges are randomly removed from the graph, *ρ*, can be provided as fixed parameters that provide users with an extra degree of control over the extent that the graph is modified. If they are set to ‘None’ (default), they are estimated during the training process in the same manner that other model parameters are estimated.

For typically sized scRNA-seq datasets, as a rule of thumb, we recommend *λ* = 0.01 for studies in which factors should closely resemble the input gene sets and *λ* = 0.1 for studies where the factors should be allowed to deviate substantially from the gene sets. Values of *δ* ranging from 0.0001 to 0.01 yield similar results, with *δ* > 0.01 providing solutions with typical highly expressed genes observed from NMF.

### Validation metrics

#### Marker list coherence metrics

To evaluate the quality of factors computed from data, we follow previous work^89,90^ and use coherence, co-occurrence of factor genes in held-out data, to evaluate the quality of the inferred factors. For a given factor, we consider the 50 top marker genes with the highest gene scores for that factor. Between every pair of genes in the top 50 markers, we compute the pointwise mutual information as

$${{{\rm{PMI}}}}({g}_{i},{g}_{j})=\log \frac{p({g}_{i},{g}_{j})}{p({g}_{i})p({g}_{j})}$$

where probabilities denote the empirical occurrences in the held-out data. Coherence is defined as the average of this quantity across the marker gene list. This metric is used in Fig. 2f. To assess the coherence of Spectra and other methods, we allocated 9,787 cells as a hold-out set to compute the coherence scores at evaluation time. The remaining 88,076 cells were used to fit the model. For each experiment, we subsampled the 88,076 cells in the training set to a size of 10,000 without replacement (repeating this process five times to recapitulate the underlying data distribution). This number was chosen to be sufficiently large subject to the constraint that each of the methods under evaluation could run in a reasonable amount of time (<2 d). For each subsampled dataset, we computed the coherence score described above with the top 50 markers, where marker lists are determined via the method suggested by the individual papers. For scHPF, we used the gene_scores function from the scHPF package to get the top 50 markers^4^. For Slalom, we multiplied the estimated parameter matrices, that is, the continuous posterior mean ${\mathbb{E}}W$ and Bernoulli posterior mean ${\mathbb{E}}Z$, as in Buettner et al.^6^. To evaluate NMF, we derive marker lists based on the absolute values of the estimated factor matrix as is standard practice^91^.

#### Reconstruction of held-out genes

To quantify the ability of methods to impute missing genes from gene sets, we ran Spectra and Slalom (scRNA-seq data preprocessing and analysis) on the full Bassez dataset but with randomly truncated gene sets. Due to Slalom’s computational demands and size of the dataset, we choose a small set of 24 gene sets to evaluate for both methods, which are chosen a priori and held fixed throughout the experiment. We hold out 40% of genes (selected randomly) from the original set and measure the fraction of these genes recovered in the top 200 genes according to Slalom and Spectra’s gene scores. To match factors to gene sets, for both methods, we find the gene set (in our full database) with the highest Szymkiewicz–Simpson overlap coefficient (overlap coefficient) to the given factor and label the factor as corresponding to that gene set. The overlap coefficient for the sets *X* and *Y* is defined as the size of the intersection divided by the size of the smaller set:

$${{{\rm{overlap}}}}(X,Y)=\frac{| X\cap Y| }{\min (| X| ,| Y| )}$$

If two factors both have the highest overlap coefficient to the same gene set, we take the one with the higher overlap coefficient. The accuracy reported is the fraction of held-out markers recovered in the top 200 highest gene scores (Fig. 2e and Extended Data Fig. 5a–c).

### Simulation experiments

#### Robustness to correlated factors

Matrix factorization methods rely on reconstruction-based objective functions that implicitly encourage the estimation of a diverse set of gene programs. As a result, when gene programs are expressed in similar contexts (for example, CD8^+^ T cell activation, exhaustion and tumor reactivity or TNF and type 2 IFN responses), matrix factorization methods often return a single program representing the combined set of correlated programs. Further, as the correlation between gene programs increases, the effective sample size of the estimation problem decreases, as most cells do not provide information to separate the gene programs. To illustrate that Spectra can incorporate prior information to maintain robust estimation in the presence of highly correlated gene programs, we simulated gene expression data from a generic factor analysis model where the cell loadings corresponding to factors 1 and 2 are simulated from a joint log normal distribution with non-zero correlation terms ranging from 0.25 to 0.99 (Extended Data Fig. 5d). Factors themselves were simulated from a half-Cauchy distribution to achieve realistic levels of sparsity and extreme values. Conditional on simulated factors and loadings, gene expression was simulated from a Poisson distribution with the mean given by the matrix product of loadings and factors. A noisy prior knowledge graph was simulated by sampling the adjacency matrix from a Bernoulli distribution with parameters given by inner products between factors (as in the Spectra model) and used as input to Spectra. For each value of the correlation, we simulated ten datasets and ran Spectra (*λ* = 0.1), NMF, scHPF and Slalom (20 top genes per factor as input). We quantified estimation accuracy by the mean Pearson correlation of ground truth factors with estimated factors across genes, both for the two correlated factors and for a third factor uncorrelated with the first two. While the unbiased methods, NMF and scHPF, correctly recover the factors when factors are weakly correlated, estimation accuracy deteriorates as the correlation increases (although the inaccurate estimation of the correlated factors does not hurt performance on the uncorrelated factors). Spectra’s use of prior knowledge allows it to separate highly correlated factors.

In more detail, in our comparative simulation, study factors are correlated in the sense that they tend to be expressed by the same cells (Extended Data Fig. 5d). We simulate ground truth factor matrices with *p* features and *K* factors with each entry independently distributed according to a half-Cauchy distribution (chosen to obtain realistically sparse factor matrices). To obtain correlated factors, the factor loadings, *α*, are independently drawn from a correlated log normal distribution:

$$\left[\begin{array}{c}{\alpha }_{1}\\ {\alpha }_{2}\\ \vdots \\ {\alpha }_{K}\end{array}\right] \sim {{{\rm{LogNormal}}}}\left(\left[\begin{array}{c}0\\ 0\\ \vdots \\ 0\end{array}\right],\left[\begin{array}{ccccc}1&\rho &0&\ldots &0\\ \rho &1&0&\ldots &0\\ 0&0&1&\ldots &0\\ \vdots &\vdots &\vdots &\ddots &\vdots \\ 0&0&0&\ldots &1\end{array}\right]\right)$$

If we denote the *N* × *K* loading matrix by *α* and the *K* × *p* factor matrix by *θ*, the count data are simulated by *X* ~ Pois(*αθ* + *ϵ*), where *ϵ* is a random noise term with variance *σ*^2^. An adjacency matrix is sampled coordinate-wise $A \sim {{{\rm{Bern}}}}({\tilde{\theta }}^{\top }\tilde{\theta })$, where ${\tilde{\theta }}_{j}=\frac{{\theta }_{j}}{\mathop{\sum }\nolimits_{k = 1}^{K}{\theta }_{jk}}$. We run 10 independent trials for 7 different levels of correlation *ρ* = {0.25, 0.5, 0.7, 0.85, 0.9, 0.95, 0.99}, totaling 70 simulated datasets. Because NMF, scHPF, Slalom and Spectra each estimate a factor matrix, we compared the estimated (normalized) factor matrices to $\tilde{\theta }$ via Pearson correlation (*y* axis of Extended Data Fig. 5d) after resolving the permutation of estimated factors that is closest to ground truth. Resolving the correct permutation for each estimate is done via finding the permutation that maximizes the average Spearman correlation between ground truth factors and estimates. Because we are interested in performance on correlated factors, we report the average correlation between estimation and ground truth for the first two correlated factors across the ten independent trials.

In our experiment, *N* = 20, *p* = 500, *K* = 3 and *σ*^2^ = 4 (a setting with low signal-to-noise ratio). Spectra is provided with the simulated matrix *A*, whereas Slalom is provided with feature sets containing the correct top 20 features of each factor. Spectra uses a *λ* value of 0.1 and *δ* value of 0. All methods are run with the correctly specified number of factors and with default parameters.

#### Biasedness of gene set averaging for overlapping gene sets

When gene sets corresponding to gene programs overlap, simple gene set averaging approaches produce false-positive program activity calls. To illustrate this phenomenon, we simulated gene expression data driven by sets of overlapping gene programs with varying degrees of gene set overlap and showed that gene set averages are increasingly biased proxies for program activity as the degree of gene set overlap increases (Extended Data Fig. 5e). Specifically, we simulated factor matrices with known sparsity patterns determined by a set of gene sets (each non-zero entry independently Exponential(16)). Each gene set is designed to have an overlap coefficient *ρ* with at least one other gene set, with *ρ* ranging from 0 to 0.75. Loadings are generated by first sampling each coordinate LogNormal(0, 1) independently and then zeroing out components that are <1 to induce sparsity. Simulated expression data are from a Poisson distribution, with the mean given by the matrix product of simulated loadings and factors.

For each possible value of the overlap coefficient *ρ* in {0.0, 0.3, 0.5, 0.75}, we create three simulated datasets and ran both Spectra and score_genes with the ground truth gene sets. Accuracy is measured by the Pearson correlation of estimated cell scores (or score_genes estimates) and the ground truth factor loadings from the data generation process (*y* axis of Extended Data Fig. 5e). In this experiment, the gene set size is fixed to 20, the number of gene sets is 10, the number of features (genes) is 500, and the number of observations is 1,000.

#### Recovery of active gene sets

We compared Spectra to Slalom, another factor analysis method that uses prior information in the form of gene sets, in a simulation experiment where we measured the ability of each method to recover the gene sets involved in the true data-generating process. Here, we followed the simulation settings of the Buettner et al. manuscript^6^ closely. First, background factors are generated from an exponential distribution, as before. To simulate sparsity, entries smaller than 2 are zeroed out. Next, loadings are generated LogNormal independently, and entries <1 are zeroed out. We then generate both active and control gene set-based factors as in Buettner et al.^6^ and described in Biasedness of gene set averaging for overlapping gene sets, where gene sets overlap with an overlap coefficient of 0.3. Loadings corresponding to active gene set-based factors are also drawn from a standard LogNormal and zeroed out if less than 1. Next genes are randomly added to gene sets and removed from gene sets to achieve a false-positive rate of 0.2 and false-negative rate of 0.2. As a measure of success, we use the area under the receiver operating characteristic curve (AUC) based on Slalom’s relevance score and Spectra’s average cell score for a given factor. Spectra was robust to increasing the number of gene sets, whereas Slalom suffered a drop in AUC as the number of active gene sets increased (Extended Data Fig. 5f).

In our experiments, the numbers of active pathways vary on the *x* axis of Extended Data Fig. 5f, the number of control pathways is 5, the gene set size is 20, the number of genes is 300, the number of cells is 300, the number of unbiased factors is 5, and the gene set overlap coefficient is 0.3.

#### Run time and memory benchmarks

All memory and run time benchmarks were performed on simulated data to allow for precise control over the number of cells, genes, factors and cell types. Data were simulated as described in Simulation experiments, closely following the settings described in Buettner et al.^6^. To benchmark run time and memory with respect to the number of cells, we scaled the number of cells in our simulated data from {300, 1,000, 5,000, 10,000, 25,000, 75,000, 100,000, 200,000} cells. The number of genes was held constant at 2,000 genes. To benchmark the methods on the number of gene sets, we scaled the n_active_pathways parameter in our simulation from {10, 20, 50, 100, 200} gene sets. Next, we note that to keep our gene set size of 20 constant with an overlap coefficient *ρ* = 0.3 between gene sets, we increase the number of genes to 3,000 genes. We used 25,000 cells for all experiments. To benchmark Spectra GPU and central processing unit (CPU) on the number of cell types, we scaled the number of cell types from {2, 4, 8, 16, 32, 64} cell types. All experiments were run using 25,000 cells and 3,000 genes. We note that due to variation in the number of epochs until convergence, we forced both Spectra CPU and GPU to run to the default 10,000 epochs to study a pessimistic but low-variance run time quantity, although convergence was generally achieved between 2,000 and 7,000 epochs. All CPU methods were run on five CPU cores (Intel Xeon Gold 6230 at 2.10 GHz), while all GPU methods were run on an NVIDIA A100 Tensor Core GPU.

### A human immunology knowledge base

Databases, such as the Gene Ontology Resource^92^, the Molecular Signatures Database^93^, the Kyoto Encyclopedia of Genes and Genomes^94^ and the Reactome database^95^, contain thousands of gene sets and their relationships, but they are noisy and often do not distinguish whether or not genes are transcriptionally regulated. For example, many genes with signaling pathway annotations are regulated at the post-translational level by phosphorylation or subcellular localization. Expression signatures in these databases are often derived from bulk sequencing data, which may not represent responses in individual cells. Moreover, the databases do not have a framework for distinguishing which gene sets are cell-type specific. To address these issues, we created an immunology knowledge base with the following criteria:

1. Genes within a gene set define a cellular process at the transcript level.
2. Gene sets represent cellular processes at the single-cell level.
3. Gene sets can be specific to a defined cell type.

Our knowledge base includes 231 gene sets representing ‘cellular processes’ (*n* = 181) to be queried by Spectra and ‘cellular identities’ (*n* = 50) to obtain replicable high-quality cell-type annotations. To generate the resource, we developed 97 gene sets, including 14 from perturbation experiments, and added these to 134 gene sets from publications and external databases, some of which we modified. Of the cellular processes, 150 apply to most cell types in the data (for example, leukocytes) and are designated as global, and 31 apply to individual cell types.

Like Spectra, the knowledge base models gene sets as a graph wherein every gene set is a node connected to all individual gene nodes within the set as well as to a cell-type node. Cell-type nodes (currently 50) are connected to ‘cellular identity’ gene sets, one for each cell type, which contain marker genes for their connected cell type. Metadata, such as the scientific publication the gene set was derived from, gene set version and original gene set authors, are stored as node properties. Cell-type nodes are organized in a hierarchy, reflecting that cell types are frequently subsets of other cell types. This hierarchy starts with a cell-type node labeled ‘all-cells’ to which gene sets for ‘cellular processes’ occurring in all cell types are connected. Thus, the knowledge base can be queried for cellular processes that can be found in all cell types (for example, glycolysis) or a cellular process specific to a cell type, such as TCR signaling, which is only present in T cells. It also allows retrieving ‘cellular identity’ marker gene sets, which define the queried cell type.

Within this resource, 150 cellular processes apply to all leukocytes, and 31 apply to individual cell types. Of all 231 gene sets, 97 were gene sets newly curated from the literature, 14 used data from perturbation experiments, 11 were adopted from the literature with modifications, and 123 were taken from the literature and external databases without changes. Gene sets correspond to diverse cellular identities (*n* = 50) and cellular processes, such as homeostasis (*n* = 9), stress response (*n* = 3), cell death and autophagy (*n* = 18), proliferation (*n* = 6), signaling (*n* = 12), metabolism (*n* = 90), immune function (*n* = 22), immune cell responses to external stimuli (*n* = 18) and hemostasis/coagulation (*n* = 3; Fig. 1b). We designed the gene sets for cellular processes to have comparable size (median *n* = 20 genes per gene sets) and relatively little overlap (median pairwise overlap coefficient of 40%) to enable dissection of a large number of cellular processes and to avoid gene set size-driven effects.

To specify Spectra input, the user first defines cell types at a granularity of interest in their single-cell expression data and retrieves the cell-type-specific cellular process gene sets and gene sets applying to all cell types from the knowledge base. Next, the user can select cellular process gene sets pertaining to all cell types in the dataset, which should be set as ‘global’ in the Spectra model.

The user indicates which cellular processes can be considered global based on which cell types are present in the dataset under study. For example, if a dataset only contains T cells, all cellular processes pertaining to leukocytes and T cells should be considered global. If cellular processes apply to more than one but not all cell types in the data, there are two options:

1. The gene set can be multiplied, and one copy can be assigned to each cell type. This will ensure that the cell scores of the resulting factors will be specific to those cell types but may result in separate factors for the same cellular process in each cell type.
2. The gene set can be set as global, which will generally result in one factor. However, cell scores for this factor may be detected in other cell types also.

Users can take advantage of the hierarchical organization of cell types in the knowledge base by adding the children or parent classes of selected cell types. For example, cellular processes for both ‘CD4 T cells’ and its parent ‘T cells’ can be retrieved and assigned to ‘CD4 T cells’, making it possible to find broader processes (for example, TCR signaling) that are specific to CD4^+^ T cells. Alternatively, cellular processes for CD4^+^ T cells and for CD4^+^ subtypes ‘T_H_1’, ‘T_H_2’ and ‘T_H_17’ can be retrieved and assigned to ‘CD4 T cells’, thereby pooling rare cell types that may not contain enough training data for the Spectra model to converge to a generalizable solution. Moreover, hierarchical classification is advantageous when cellular processes are ambiguously or incorrectly assigned. For example, CD4^+^ T cell subtypes are often presented as distinct lineages with distinct cellular processes. However, mixed CD4^+^ T cell subtypes have been reported, such as cells possessing both T_H_1 and T_H_2 polarization cellular processes^96^, suggesting that CD4^+^ T cells can be described using combinations of purportedly subtype-specific processes.

The newest version of our human immunology knowledge base is available on GitHub as the Cytopus Python package^97^. This includes 20 additional cellular processes mostly for non-immune cells, such as fibroblasts (*n* = 7), which we curated from a list of perturbation experiments on bulk-sorted immune cells^98^. Users can load our default or a custom knowledge base using the KnowledgeBase class build on a NetworkX object. Cytopus includes methods to retrieve gene sets and corresponding cell types and can visualize them as a graph and convert them into a Spectra-compatible dictionary. The ‘celltypes’ method retrieves a list of available cell types, ‘processes’ generates a dictionary of all ‘cellular processes’ gene sets, and ‘identities’ generates a dictionary of all ‘cellular identity’ gene sets. Gene set metadata (for example, author, topic, date of generation and version) can be accessed as node properties of the gene sets. The get_celltype_processes method retrieves cell-type-specific ‘cellular processes’ based on a user-provided list of cell types at the desired granularity (generally all cell types contained in the data).

Full Cytopus documentation can be found at https://github.com/wallet-maker/cytopus. The tumor-infiltrating leukocyte gene sets used in the paper are included in the Spectra package at https://github.com/dpeerlab/spectra and are in Supplementary Table 1.

### scRNA-seq data preprocessing and analysis

An overview of all datasets used in this study and relevant patient metadata can be found in Supplementary Table 5.

### PBMC dataset

The original dataset^8^ consists of scRNA-seq data from PBMCs from four healthy donors after incubation (1–6 h) in IFNγ, LPS or the protein kinase C and TCR stimulation mimetic PMA. For three donors and at the 6-h time point, they added Golgi inhibitors (GIs), which prevent exocytosis of cytokines from PBMCs after perturbation and secondary paracrine signaling events, and compared them to control cells treated with GIs alone. Thereby, the gene expression changes in the GI-treated perturbations compared to the GI-treated control cells can be attributed to direct signaling of the applied perturbations alone. We used the GI-treated conditions as a ground truth to benchmark factorization methods. Information on ethics oversight was not available in the original manuscript^8^.

We obtained preprocessed count matrices (23,754 cells, four individuals) from the Gene Expression Omnibus (GEO; accession number GSE178431) from the Kartha et al.^8^ PBMC perturbation scRNA-seq dataset. We normalized gene expression counts to median library size and log 1*p* transformed the data. We selected the 10,000 most highly variable genes using scanpy’s pp.highly_variable_genes function with the seurat_v3 method on raw counts. To avoid discarding genes relevant to cell typing, we added a manually curated list of cell typing markers to highly variable genes (Supplementary Table 6). We then calculated the neighborhood graph on the first 50 principal components using these highly variable genes, which explained 27.87% of the total variance, and calculated a UMAP embedding on this neighborhood graph.

To get coarse immune cell types, we clustered the data in principal component space using the scanpy implementation of phenograph. We chose the *k* = 40 parameter for PhenoGraph because of its ability to delineate immune cell from non-immune cell populations while showing stable clustering in a window of adjacent *k* parameters (pairwise rand indices > 0.7). We then annotated the clusters into coarse immune cell types (monocytes, T cells/innate lymphoid cells and B cells/plasma blasts) by assessing the mean marker gene expression per cluster (Supplementary Table 2).

#### Running Spectra

To run Spectra, we retrieved 188 input gene sets pertaining to PBMC data from the newest version of our Cytopus knowledge base (10.5281/zenodo.7306238). This included gene sets for signaling/response programs to the ground truth perturbations (IFNγ response, LPS signaling in monocytes/macrophages and TCR activation in T cells). We fitted Spectra on the union of the 10,000 most highly variable genes and the input gene sets for a total of 11,840 genes using the following parameters: *λ* of 0.01, *δ* of 0.001 and *ρ* of 0.001. We obtained a total of 196 factors and found 1 factor for each of the input gene sets related to each perturbation according to our criteria below (overlap coefficient of the top 50 marker genes with input gene set > 0.2). We calculated the average cell score per cell type and sample and compared the perturbed and unperturbed conditions. Spectra was run on a compute cluster with 64 CPU cores (Intel Xeon Gold 6230 CPU at 2.10 GHz) with 512 GB RAM.

#### Running Slalom

For Slalom, expression data were preprocessed the same as Spectra. Because Slalom’s run time scales linearly with the number of gene sets (Fig. 2g), we had to subset the number of gene sets used to run Slalom on our dataset of interest. We provided Slalom only with the three gene sets corresponding to the investigated perturbations (LPS, IFNγ and PMA) plus ten additional factors. These gene sets were used to determine the I parameter of the Slalom initFA() function. The following additional input parameters were used: nHidden = 0, nHiddenSparse = 0, do_preTrain = False, minGenes = 1 and pruneGenes = False, with all other options set to default values.

#### Running expiMap

For expiMap, expression data were preprocessed the same as Spectra. We used expiMap’s default parameters as shown in the tutorials of the scArches GitHub repository (https://github.com/theislab/scarches; version as of 20 March 2023). We provided expiMap with the same gene sets as Spectra. When using the default parameters, expiMap cannot learn new genes involved in gene programs related to the input gene sets nor can it learn new factors in the reference data, but only in the mapped query.

### Immuno-oncology datasets

To study Spectra in an immuno-oncology context, we used two published scRNA-seq datasets of tumor-infiltrating leukocytes from female individuals with breast cancer treated with immunotherapy. We chose this immuno-oncology context for the following reasons:

1. The abundant prior knowledge of cellular processes and well-characterized cell types in tumor-infiltrating leukocytes enabled us to leverage the full power of gene set and cell-type priors.
2. The availability of before- and on-treatment samples to test the sensitivity of factor cell scores to environmental perturbation with anti-PD-1/PD-L1 therapy.
3. The clinical need for detecting cellular processes affected by anti-PD-1 in humans to improve current immunotherapy strategies.
4. The availability of two studies in similar biological settings to enable validating findings in an independent dataset.

#### Bassez dataset

Bassez et al.^10^ was a prospective window-of-opportunity study reporting scRNA-seq as an exploratory endpoint. The authors analyzed scRNA-seq data from whole-tumor single-cell suspensions from 42 individuals with operable breast cancer before and after anti-PD-1 immunotherapy (pembrolizumab, NCT03197389). Individuals received neoadjuvant chemotherapy as per standard of care (chemotherapy *n* = 11, no chemotherapy *n* = 31), followed by a single dose of anti-PD-1. Breast resections were performed 7–14 d after anti-PD-1 treatment. Tissue from pre-anti-PD-1 biopsies (7–15 d before surgery) and from surgical resections was processed for scRNA-seq. The study was approved by the institutional review board of University Hospital Leuven (S60100). As a surrogate for response to therapy, the authors of the original study quantified the clonal expansion of T cells under therapy on the patient level using paired single-cell TCR sequencing; we used these annotations to find response-associated cellular processes in the data. The authors categorized participants as either exhibiting (we refer to as responders) or lacking (we refer to as non-responders) T cell clonal expansion under therapy. To classify participants, the authors quantified the number of expanding T cell clones (T cells with identical TCR sequences) per participant, labeling participants with >30 expanding clones as responders and those with ≤30 expanding clones as non-responders. A T cell clone had to fulfill the following two criteria to be labeled as expanded:

1. Detected at least twice in the participant’s on-treatment sample.
2. More frequent in the participant’s on-treatment samples than in the pretreatment sample either by the absolute cell number in that clone or by the cell number in that clone relative to the number of cells with a TCR detected.

#### Zhang dataset

Zhang et al.^23^ was a retrospective clinical study analyzing tumor-infiltrating leukocyte scRNA-seq data of pre-, on- and post-therapy samples from 15 female individuals with advanced breast cancer receiving either anti-PD-L1 (atezolizumab) combined with chemotherapy (paclitaxel, *n* = 8) or chemotherapy alone (paclitaxel, *n* = 7). The study was approved by the Chinese Academy of Medical Sciences ethics review board (18-216/1794). Notably, participants received corticosteroid premedication for paclitaxel. The authors assessed participant response to immunotherapy using radiological response according to RECIST v1.1 criteria^99^. RECIST v1.1 criteria are the standard criteria used for drug approval-relevant clinical trials and standard clinical management of individuals with metastatic solid tumors. The RECIST criteria classify individuals into responders (combining partial and complete response labels) and non-responders (combining progressive and stable disease labels) based on the change in the sum of tumor lesion diameters under therapy. We used this classification to identify response-associated cellular processes in the Zhang dataset. Relevant clinical variables distinguishing the Bassez and Zhang data are indicated below.

Bassez:

Operable disease: yes

*n* = 31 treated with anti-PD-1

*n* = 11 treated with chemotherapy and anti-PD-1

Received corticosteroids: no

Zhang:

Operable disease: no

*n* = 8 treated with chemotherapy and anti-PD-L1

*n* = 7 treated with chemotherapy

Received corticosteroids: yes

#### Processing strategy

To minimize systematic differences in cell-type annotations and normalization of gene expression counts, we performed the same preprocessing for the Bassez^10^ and Zhang^23^ datasets. After basic filtering, removing residual low-quality cells and doublets and subsetting to leukocytes with scanpy^2^, we normalized the data using scran^77^. We hierarchically annotated cell types in the data by first labeling major immune subsets (T cells/innate lymphoid cells, B cells/plasma cells and myeloid cells) by clustering on the most dominant principal components only. We then partitioned the data into these major immune subsets, renormalized the data within every subset using scran and clustered on more principal components to annotate granular cell types. We then combined the annotated data from major immune subsets for joint analysis using Spectra. We have outlined the details of the analysis strategy below.

#### Retrieving single-cell gene expression data

Count matrices of the Bassez data^10^ were kindly provided by the authors and are also available here (226,635 cells). Raw read counts are available in the European Genome–Phenome Archive (EGA; accession numbers EGAS00001004809 and EGAD00001006608). The count matrices for the Zhang data^23^ were downloaded from GEO using the accession number GSE169246 (489,490 cells).

#### Removing low-quality cells

To prepare the data for clustering, we removed cells with less than 200 genes per cell and genes observed in less than 20 cells as well as mitochondrial and ribosomal genes. This filtering procedure removed 2,971 and 203 genes, resulting in a total of 22,639 and 20,898 genes in the Bassez^10^ and Zhang^23^ datasets, respectively. We defined doublets in the data by running DoubletDetection^100^ for each sample individually using standard parameters (clustering algorithm: PhenoGraph; *P* value threshold: 1 × 10^–16^; voter threshold: 0.5). DoubletDetection detected 3,270 (1.4%) and 12,760 (2.6%) doublets as well as 27 (0.01%) and 8 (0.001%) ambiguous doublets in the Bassez^10^ and Zhang^23^ datasets, respectively, which we removed from the data.

#### Retrieving tumor-infiltrating leukocytes for downstream annotation

While the Zhang^23^ data contained sorted tumor-infiltrating leukocytes, the Bassez^10^ data contained unsorted whole-tumor single-cell suspensions. To retrieve immune cells from the Bassez^10^ data for downstream annotation, we first performed standard median library size normalization and log 1p transformed the data so that the normalized expression of every gene *j* in cell *i* is ${x}_{ij}^{{\prime} }$, and the median of the sum of gene expression counts across all cells is $med\left(\mathop{\sum }\nolimits_{j = 1}^{n}{x}_{j}\right)$:

$${x}_{ij}^{{\prime} }=\ln \left[med\left(\mathop{\sum }\limits_{\,j=1}^{n}{x}_{j}\right)* \frac{{x}_{ij}}{\mathop{\sum }\nolimits_{j = 1}^{n}{x}_{ij}}+1\right]$$

We then clustered the data using PhenoGraph^101^ on the most dominant principal components, which we selected using the knee point of the principal component versus explained variance curve (calculated using the kneed package v.0.7.0 (ref. ^102^)) or the lowest number of principal components explaining ≥20% of the total variance, whichever was higher. Using this procedure, we clustered the data with PhenoGraph on the first 26 principal components, explaining 20.1% of the total variance. We chose the *k* = 80 parameter for PhenoGraph because of its ability to delineate immune cell from non-immune cell populations while showing stable clustering in a window of adjacent *k* parameters (pairwise rand indices > 0.7). We then subsetted leukocytes for further analysis by their marker gene expression (myeloid cells, T cells, innate lymphoid cells, B cells and plasma cells) per PhenoGraph cluster (Supplementary Table 2).

#### Annotating tumor-infiltrating leukocytes

We renormalized leukocytes in the Bassez^10^ and Zhang^23^ data using scran because median library size normalization can generate artificial differential gene expression between cells of different library size, such as leukocytes^77^. After testing all genes and a range between 5,000 and 15,000 highly variable genes, we selected the top 15,000 highly variable genes for the Bassez^10^ data and all genes for the Zhang^23^ data, which led to the best separation of major immune cell subtypes using scanpy’s pp.highly_variable_genes function with the seurate_v3 method on raw counts. To avoid discarding genes relevant to cell typing, we added a manually curated list of 458 cell-typing markers to highly variable genes (Supplementary Table 6). We then repeated the clustering procedure outlined above (Bassez^10^: 24 principal components explaining 20.1% of total variance; Zhang^23^: 52 principal components explaining 20% of total variance; *k* = 50) and annotated major immune cell subsets (T cells/innate lymphoid cells, B cells/plasma cells and myeloid cells) by assessing their mean marker gene expression per cluster (Supplementary Table 2). To obtain more granular annotations, we partitioned the data into major immune subtypes (T cells, innate lymphoid cells, B cells/plasma cells and myeloid cells), renormalized each subtype using scran, recalculated highly variable genes and principal components and clustered as described above. The following processing parameters for each subtype were used: Bassez data, numbers of highly variable genes and marker genes: 7,500, 7,500 and 15,000 for T cells/innate lymphoid cells, B cells/plasma cells and myeloid cells, respectively; numbers of principal components: 24, 10 and 16 for T cells/innate lymphoid cells, B cells/plasma cells and myeloid cells, respectively; variance explained by principal components: 20.3%, 20.1% and 20.1% for T cells/innate lymphoid cells, B cells/plasma cells and myeloid cells, respectively; *k* parameter for clustering: 30, 40 and 20 for T cells/innate lymphoid cells, B cells/plasma cells and myeloid cells, respectively; Zhang data, numbers of highly variable genes and marker genes: 19,379, 19,379, 10,000 and 18,888 for T cells/innate lymphoid cells, innate lymphoid cells, B cells/plasma cells and myeloid cells, respectively; numbers of principal components: 100, 100, 17 and 23 for T cells/innate lymphoid cells, innate lymphoid cells, B cells/plasma cells and myeloid cells, respectively; variance explained by principal components: 17.9%, 17.7%, 20.4% and 20.1% for T cells/innate lymphoid cells, innate lymphoid cells, B cells/plasma cells and myeloid cells, respectively; *k* parameter for clustering: 40, 40, 60 and 20 for T cells/innate lymphoid cells, innate lymphoid cells, B cells/plasma cells and myeloid cells, respectively.

We then annotated granular immune cell types by assessing the mean marker gene expression per cluster (Supplementary Table 2). In the Bassez^10^ data, we detected clusters with low library size and lower complexity of gene–gene correlation patterns at this step (5,509 cells), which we removed from the data. Finally, we combined the annotated major immune subtypes for downstream joint analysis.

#### Running Spectra

After the filtering and preprocessing steps above, the Bassez data had 97,863 cells^10^, and the Zhang data had 150,985 cells^23^. To run Spectra, we restrict the number of genes using scanpy’s highly_variable_genes function with the cell_ranger method, selecting the 3,000 most highly variable genes. We removed several genes that are highly abundant and may originate from ambient RNA in many cell types, thus adding noise to the analysis. This included mitochondrial, ribosomal, immunoglobulin (genes starting with *IGHM*, *IGLC*, *IGHG*, *IGHA*, *IGHV*, *IGLV* and *IGKV*), TCR variable domains (genes starting with *TRBV*, *TRAV*, *TRGV* and *TRDV*) and hemoglobin genes (genes starting with *HB*). The total numbers of genes used (the union of genes included in a gene set and highly variable genes) were 6,397 for the Bassez dataset^10^ and 6,398 for the Zhang dataset^23^.

One hundred and eighty-one gene sets from our knowledge base were then converted into weighted adjacency matrices. One of Spectra’s strongest features is its ability to meaningfully modify the input gene–gene knowledge graph (gene sets) in a data-driven matter. With the influence parameter *λ* set to 0.01, the median overlap coefficient across all factors in the Bassez^10^ data was 88%, with 25% of factors relevantly deviating from the gene sets (overlap < 70%) and 7% of factors bearing little resemblance to the input gene sets (overlap < 20%). With the influence parameter set to 0.1, the median overlap coefficient across all factors was 82%, with 42% of factors relevantly deviating from the gene sets (overlap < 70%) and 12% of factors with an overlap of less than 20%. In terms of graph edit distance to the input graph (defined as the mean absolute difference between input and output graphs), at *λ* set to 0.1, we had 0.011, and at *λ* set to 0.01, we had 0.0095 with diminishing returns in graph edit distance for lower *λ* (0.0095 again for *λ* = 1 × 10^–4^ and 0.0094 for *λ* = 1 × 10^–5^). For the analyses described below, we used an influence parameter *λ* between 0.1 and 0.01 depending on whether we wanted more (0.01) or less (0.1) adherence to the input gene sets. Because we obtained very similar results with these parameters in two independent datasets, it is likely that this also constitutes a good default for other datasets. Spectra was run on a compute cluster with 64 CPU cores (Intel Xeon Gold 6230 CPU at 2.10 GHz) with 512 GB RAM.

#### Running Slalom

Because Slalom’s run time scales linearly with the number of gene sets (Fig. 2g), we had to subset the number of gene sets used to run Slalom on our datasets of interest (*n* = 20 gene sets, run time 63.49 CPU hours, 40 GB of memory on the Bassez^10^ dataset). Expression data were preprocessed identically to Spectra. To compare results of Spectra and Slalom, we chose a subset of 20 gene sets of scientific relevance to the immune microenvironment under immune checkpoint blockade for the Bassez^10^ and Zhang^23^ datasets: CD8^+^ T cell tumor reactivity, type II IFN response, myeloid angiogenic effectors, post-translational modification, MHC class I presentation, G2/M transition, oxidative phosphorylation, type I IFN response, macrophage IL-4/IL-13 response, glycolysis, DNA synthesis, G1/S transition, lysine metabolism, MHC class II presentation, hypoxia response, pentose phosphate pathway, CD8 terminal exhaustion, PD-1 signaling, TCR activation and cytotoxicity effectors. These gene sets were used to determine the I parameter of the Slalom initFA() function. The following additional input parameters were used: nHidden = 0, nHiddenSparse = 0, do_preTrain = False, minGenes = 1, pruneGenes = False, with all other options set to default values.

#### Running scHPF

scHPF was run with the following commands using the defaults in the class constructor of the scHPF package. from scHPF import *: model = scHPF(nfactors = K); model.fit(X).

#### Running expiMap

When using the default parameters, expiMap^9^ cannot learn new genes involved in gene programs related to the input gene sets nor can it learn new factors in the reference data but only in the mapped query. We note that most demonstrations in the expiMap manuscript^9^ are based on these default parameters and do not involve adaptation to the data.

For the immunology datasets, where the specific task evaluated involved learning new genes and new factors, we modified the default parameters according to Lotfollahi et al.^9^. We refer to this mode as soft mode. These parameters include setting soft_mask = True in the expiMap model scarches.models.EXPIMAP and setting an L1 penalty using the alpha_l1 parameter of the model.train method, which enables the latent nodes to use genes absent from the input gene sets with an L1 regularization. The alpha_l1 parameter was increased in steps of 0.1 starting from 0.5 until the share of inactive genes exceeded 0.95 (this information can be visualized by the print_stats parameter in the .train method). Using this strategy, we selected an alpha_l1 of 0.8. For the Extended Data Fig. 5b,c reconstruction experiment where the task involved recovering held-out genes, we used an alpha_l1 parameter of 0.4 because expiMap showed the strongest performance with this parameter in a similar experiment performed by the expiMap authors (Extended Data Fig. 7 in Lotfollahi et al.^9^). However, for most of our gene sets and in contrast to Spectra, expiMap did not recover a meaningful proportion of our input gene sets (Extended Data Fig. 5b,c). This is despite the fact that the Spectra model was complicated by including 16 new factors, while we did not add any new factors for expiMap^9^.

To learn new factors, one has to provide additional parameters, which is what we did for analyzing the Bassez^10^ dataset except for the reconstruction experiment in Extended Data Fig. 5b,c. We added 16 new factors, the same number as for Spectra (n_ext, set to 16). We set the L1 regularization coefficient for these nodes gamma_ext to 0.6, enabled the Hilbert–Schmidt independence criterion regularization (use_hsic = True, hsic_one_vs_all = True) and provided the Hilbert–Schmidt independence criterion regularization coefficient beta = 3. Because expiMap removed relevant input gene sets, we had to perform two additional modifications of the steps provided in the tutorial for the soft mode. We increased the number of highly variable genes (4,000 instead of 2,000) to retain the input lysine metabolism gene set and decreased the minimum gene set size from 13 to 8 to retain our tumor reactivity gene set. We provided expiMap with the identical 181 gene sets used for Spectra. Because expiMap removes smaller gene sets in preprocessing steps, the final number of gene sets used for the model fit was 142, which resulted in 158 factors, including 16 new factors and 3 inactive factors.

#### Running NMF

NMF was run using the scikit learn package (sklearn.decomposition.NMF) with default parameters, specifically nmf = NMF(n_components = *k*) and nmf.fit(*X*.astype(float)), where *k* is the number of factors and *X* is the processed expression matrix.

#### Running netNMFsc

netNMFsc^7^ was run with default parameters; however, max_iters was set to 100,000 as convergence was never achieved at the default tolerance level (1 × 10^–2^) at the default 20,000 iterations. Specifically the following operations were used: operator = netNMFsc.netNMFGD(*d* = *k*, max_iter = max_iters); operator.N = adj_matrix; operator.*X* = *X*.T and W = operator.fit_transform(), where adj_matrix is the global graph provided to Spectra, *X* is the processed expression matrix, and *k* is the number of factors.

#### Assigning factor labels

Factor labels were assigned using the overlap coefficient of the top 50 marker genes (genes with the highest gene scores) with each gene set. We observed a bimodal distribution of overlap coefficients, with one group of factors centered close to 0 and one group of factors centered close to 1 (Extended Data Fig. 2). We therefore chose a threshold of 0.2 to separate high-overlap from low-overlap factors. For every factor, if the maximum overlap coefficient was >0.2, we assigned the gene set label with the maximal overlap coefficient to that factor, and if the maximum overlap coefficient was ≤0.2, we did not assign a label to that factor.

#### Aggregating cell scores at the sample level

To aggregate cell scores at the sample level, we calculated either the mean or the mean of the positive cells. The latter was chosen for Spectra and scHPF, which show bimodal cell score distributions with one mode centered around 0. The mean will be skewed toward the more frequent zero mode and may therefore be inappropriate to estimate the central tendency of the distribution. Positive cells were defined as cells with a cell score of >0.001. This threshold was defined empirically to separate the positive and zero mode by inspecting the distributions of multiple factors. If all cells showed a cell score of ≤0.001 for a given gene program, the mean of the positive fraction was set to 0 for that gene program. Because expiMap and Slalom can take negative values, we used the mean value for these methods.

### Lung cancer datasets

#### Caushi dataset

The Caushi et al.^30^ study performed paired scRNA-seq and single-cell TCR sequencing of 16 individuals with primary non-small cell lung cancer (560,916 cells). Moreover, PBMCs were pulsed with different peptides (specific for viral or tumor neoantigens), and reactive, expanding T cell clones and their TCR sequences were identified using the MANA functional expansion of specific T cells assay^103^. The authors thereby identified TCR sequences of MANA-, Epstein–Barr virus- and influenza-reactive T cells. They used these TCR sequences to identify tumor (MANA) and virus-reactive T cells in the lung cancer tissue. The study was approved by the institutional review boards at Johns Hopkins University and Memorial Sloan Kettering Cancer Center.

Preprocessed data were obtained from the original study’s authors^30^. The processed data can also be obtained from GEO under accession number GSE173351. Because cell-type annotations were not available, we obtained the original study authors’ cluster labels (details on preprocessing and clustering are in Caushi et al.^30^). We reannotated the original authors’ 15 clusters using marker genes (Supplementary Table 2).

To preprocess the data for Spectra, we normalized raw counts to median library size and applied log 1p transformation. We restricted the number of genes using scanpy’s highly_variable_genes function with the cell_ranger method to the 3,000 most highly variable genes. We retrieved a total of 168 input global gene sets (*n* = 152) and T cell subtype-specific gene sets (CD4^+^ T cells, CD8^+^ T cells and regulatory T cells, *n* = 12) from the newest version of our Cytopus knowledge base (https://zenodo.org/record/7306238). We took the union of the highly variable genes and the genes included in these input gene sets for a total of 6,838 genes^30^ used for fitting the Spectra model using the following parameters: *λ* = 0.1, *δ* = 0.001 and *ρ* = 0.001. We ran Spectra on these data and obtained 173 factors, 1 of which matched the CD8^+^ T cell tumor reactivity gene set according to the criteria described above in Assigning factor labels. Spectra was run on a computer cluster with 64 CPU cores (Intel Xeon Gold 6230 CPU at 2.10 GHz) with 512 GB RAM. We plotted and compared the tumor reactivity factor cell scores in 1,151 CD8^+^ T cells with available TCR specificity information grouped by target antigen (Epstein–Barr virus, influenza virus and MANA). We found that this tumor reactivity factor was almost exclusively expressed in MANA-specific T cells.

#### Salcher atlas

The Salcher non-small cell lung cancer atlas combined scRNA-seq data of whole-tumor single-cell suspensions or tumor-infiltrating leukocytes from 19 independent studies (1,283,972 cells from 318 individuals). They also homogenized cell-type annotations and metadata between datasets. The study was approved by the institutional review board at Medical University Innsbruck (AN214-0293 342/4.5).

Preprocessed data, including unnormalized gene expression counts, were obtained from Zenodo (https://zenodo.org/record/7227571) for the Salcher et al. lung cancer scRNA-seq atlas^71^. The authors’ cell-type annotations were summarized after vetting them for relevant marker expression profiles (Supplementary Table 2).

To run Spectra, we restricted the number of genes using scanpy’s highly_variable_genes function with the cell_ranger method, selecting the 3,000 most highly variable genes with the batch_key option, which calculates highly variable genes in each dataset in the atlas separately and then merges them based on in how many datasets they are captured. We removed several genes that are highly abundant and may originate from ambient RNA in many cell types, thus adding noise to the analysis. This included mitochondrial, ribosomal, immunoglobulin (genes starting with *IGHM*, *IGLC*, *IGHG*, *IGHA*, *IGHV*, *IGLV* and *IGKV*), TCR variable domains (genes starting with *TRBV*, *TRAV*, *TRGV* and *TRDV*) and hemoglobin genes (genes starting with *HB*). We retrieved a total of 198 input gene sets from the newest version of our Cytopus knowledge base (https://zenodo.org/record/7306238). The total number of genes used (the union of genes included in a gene set and highly variable genes) was 7,322. We normalized and log 1p transformed the gene expression counts and ran Spectra with the parameters *λ* = 0.01, *δ* = 0.001 and *ρ* = 0.001 and obtained one factor each for CD8^+^ T cell tumor reactivity and lysine metabolism. We also obtained one factor that shared 20 of the top 50 marker genes with the macrophage invasion factor from the Bassez^10^ dataset (overlap coefficient = 0.4). We then calculated the overlap of these factors with the factors obtained from the Bassez^10^ and Zhang^23^ datasets (Fig. 6c). Spectra was run on a computer cluster with 128 CPU cores (Intel Xeon Gold 6230 CPU at 2.10 GHz) with 1,024 GB of RAM.

To calculate embeddings for plotting UMAPs, we calculated the neighborhood graph (*k* = 10) on the first 50 principal components using the top 3,000 highly variable genes, which explained 45.92% of the total variance, and calculated a UMAP embedding on this neighborhood graph using the scanpy implementation (Fig. 6a).

We obtained study-specific factors by their cross-study entropy. We first removed spuriously expressed factors with ≤100 positive (cell score > 0.001) cells. For each remaining factor, we then calculated the entropy of study label proportions in factor-positive cells. We selected the factors that showed a cross-study entropy higher than 2.0794, which is the entropy for a hypothetical factor where positive cells are absent in 11 of the 19 studies analyzed and where they are equally distributed among the remaining 8 studies. This resulted in 11 global (Fig. 6b) and 3 cell-type-specific factors.

To assess the stability of our lysine factor across studies, we plot its *z*-scored (across cell types) mean expression per cell type (Fig. 6d). We also calculated its mean expression per sample and cell type and compared the expression in plasma cells to the expression in other cell types using Wilcoxon matched-pairs signed-rank tests (Fig. 6d). To compare cell scores of the CD8^+^ T cell tumor reactivity and macrophage invasion factors in clinically relevant patient subgroups, we calculated their mean cell scores in positive (cell score > 0.001) CD8^+^ T cells and macrophages, respectively. We then compared these aggregated cell scores in ever smoker versus never smoker and wild-type *EGFR* versus mutated *EGFR* tumors. We excluded individuals with other driver mutations from wild-type *EGFR* tumors because these tumors have different clinical and biological behaviors. Correction for covariates was not performed because many covariates (sex and age) were only available for a small fraction of individuals with available smoking and *EGFR* status (only for 13 individuals with *EGFR-*mutated tumors were both age and sex information were available).

### Classifying new and modified factors

We classified all factors as new, modified or unspecified based on their input gene–gene knowledge graph dependency parameter *η*. The dependence parameter is a scalar value between 0 and 1 that quantifies its reliance on the input gene set graph. We observed a bimodal distribution of *η*, with one group of factors centered close to 0 and another group of factors centered close to 1 (Extended Data Fig. 2a). We therefore chose a threshold of 0.25 to separate high-dependence from low-dependence parameters. We defined new factors as factors with a graph dependency parameter of *η* < 0.25 and modified factors as factors with a graph dependency parameter of *η* ≥ 0.25.

### Analyzing breast cancer-infiltrating leukocytes

We compared the ability of Spectra, Slalom and scHPF to retrieve features of tumor-infiltrating immune cells. We ran the three algorithms on all leukocytes in the Bassez dataset, as described above, using a *λ* parameter of 0.01 for Spectra. We also ran Spectra on the Zhang dataset using a *λ* parameter of 0.01. For cells with high library size, such as macrophages (Bassez dataset median library size = 8,038), we calculated gene scores for Spectra factors using an offset of 1, which retrieved more stably expressed genes (for example, mean scran-normalized expression of the top 50 marker genes of the macrophage factor 182: 1.15 with offset versus 0.41 with no offset). For remaining analyses with lower library size, such as T cells (Bassez dataset median library size = 3,127) or B cells (Bassez dataset median library size = 3,954), we calculated gene scores for Spectra using an offset of 0, which allowed for more sensitive retrieval of lowly expressed genes, such as transcription factors involved in tumor reactivity and exhaustion (for example, *EOMES* and *TOX*) as well as metabolic processes (for example, *PIPOX* and *BBOX1*).

#### Visualizing scRNA-seq data

To visualize individual genes in embeddings and account for sparsity in scRNA-seq data, we imputed gene expression using scanpy’s implementation of MAGIC^104^ with a *t* parameter of 3 and the exact solver (Fig. 2d and Extended Data Figs. 3c, 6b, 7b and 8b). For visualizing all leukocytes, we calculated t-SNE embeddings on 57 principal components explaining 25.0% (Bassez dataset) or 55 principal components explaining 20% of variance (Zhang dataset) with standard parameters including a learning rate of 1,000 using the scanpy implementation (Figs. 2b and 4d and Extended Data Figs. 3a, 4a, 7e and 8a).

#### Gene set enrichment analysis

To find the most representative factors for a gene set in Spectra, Slalom and scHPF, we performed gene set enrichment analysis for the exhaustion and tumor reactivity input gene sets in the top 50 marker genes (genes with highest gene scores) of every factor using gseapy’s enrichr function^105^ (Extended Data Figs. 6e and 7c). The enrichr function calculates enrichment using a hypergeometric test to calculate the probability of drawing the observed number of genes belonging to a gene set of interest when sampling from a pool of all genes without replacement (here, the union of the 3,000 most highly variable genes plus the genes contained in the gene sets; see Running Spectra). We calculated the enrichment of gene sets in the top 50 markers genes (genes with highest gene scores) for each factor

$$p(k;N,K,n)=\frac{\left(\begin{array}{c}K\\ k\end{array}\right)\left(\begin{array}{c}N-K\\ n-k\end{array}\right)}{\left(\begin{array}{c}N\\ n\end{array}\right)}$$

where *n* is the number of factor marker genes (here, 50), *k* is the number of genes from the gene set in the top 50 marker genes in the factor, *N* is the total number of genes contained in the data, and *K* is the number of genes from the gene set contained in the data. From this, we calculated an FDR using the Benjamini–Hochberg correction. We assumed that the factors with the lowest FDR for enrichment were representative of the respective gene sets if the FDR was <0.05.

#### CD8+ T cell analysis

We took the subset of CD8^+^ T cells (11 clusters) from the Bassez dataset to explore CD8^+^ T cell tumor reactivity (tumor reactivity) and CD8^+^ T cell exhaustion (exhaustion). The most representative factors for the tumor reactivity and exhaustion gene sets were retrieved using the gene set enrichment procedure described above (Gene set enrichment analysis) for each factor analysis method (Extended Data Fig. 6e). Spectra factors were also compared to expression scores for the tumor reactivity and exhaustion gene sets using scanpy’s score_genes function (Fig. 3b and Extended Data Fig. 6b). To find genes driving score_genes expression, we calculated the covariance of all genes within the tumor reactivity or exhaustion gene sets with the tumor reactivity and exhaustion gene scores (Extended Data Fig. 6b). To visualize force-directed layouts, we used scanpy’s tl.draw_graph function and the ForceAtlas2 method on a nearest neighbors graph computed on CD8^+^ T cells using scanpy’s tl.neighbors function with *n* = 10 nearest neighbors (Fig. 3a–c and Extended Data Figs. 3b,c and 6b). Contour plots were created using seaborn’s jointplot kernel density estimation with standard parameters (Fig. 3b,c and Extended Data Fig. 6f). We compared Spectra’s ability to deconvolve processes of tumor reactivity and exhaustion (Fig. 3) with the factorization methods scHPF, Slalom and expiMap. In contrast to Spectra, Slalom only found a factor highly enriched for exhaustion genes in the Bassez dataset, which overlaps with the highest scoring factor for tumor reactivity by 35 genes (Extended Data Fig. 6e). scHPF factors are not enriched for either reactivity or exhaustion gene sets, whereas expiMap identified and successfully deconvolved the two factors (Extended Data Fig. 6e). However, only Spectra was able to distinguish a clonally expanding tumor-reactive T cell population that is specific to responders (Extended Data Fig. 6f). Moreover, Slalom, scHPF and expiMap tumor reactivity and Spectra exhaustion factors failed to associate with patient-level response, defined as a significant difference between expression in responders and non-responders before or during ICT (Extended Data Fig. 6g).

#### Metabolism analysis

We assessed the expression pattern of metabolic factors across cell types and found highly specific expression of the lysine metabolism program in plasma cells. In the Bassez dataset, we noticed a small (*n* = 114 cells, 0.1% of all cells, 3% of all plasma/B cells) group of heterotypic doublets expressing plasma cell and T cell markers (*CD3E*, *CD3D*, *CD3G*, *IGHG4* and *IGHG1*), which was not apparent in previous analyses and not detected by DoubletDetection. We removed these cells from further analyses involving plasma cells (Fig. 4). We also inspected the mean expression per cell type of the MAGIC^104^-imputed (scanpy implementation, *t* = 3, exact solver) top 50 individual marker genes of the lysine factor genes with highest gene scores (Extended Data Fig. 7b). Slalom^6^ identified a factor in plasma cells with worse resemblance to lysine metabolism (Extended Data Fig. 7c), which is contaminated with cell-type markers (Extended Data Fig. 7d), possibly because Slalom does not use cell-type-specific gene scalings. expiMap^9^ and scHPF^4^ factors were homogeneously expressed across cells or not enriched in lysine metabolism genes, respectively (Extended Data Fig. 7c–e).

#### Macrophage analysis

To analyze differentiation gradients in macrophages and to capture all possible maturation stages, we retained the subset of 18 clusters (12,132 cells) annotated as mature macrophages (12 clusters) or more immature myeloid-derived (suppressor) cells/monocytic cells (6 clusters) in the Bassez dataset for further analysis (Supplementary Table 2). We embedded the data using DCs, which preserve differentiation trajectories better than many common linear and nonlinear dimensionality reduction techniques^53^. Using a classification strategy, we selected the DCs that best captured differentiation from more monocytic states to macrophages while separating individuals with (responders) and without (non-responders) clonal T cell expansion (Fig. 5a,b and Extended Data Fig. 8a,b,d). For every pair of the first 20 DCs, we performed (1) a linear regression with the DCs as the independent variable and the scran-normalized expression of the monocyte marker *S100A8* as the dependent variable and (2) a logistic regression with the DCs as the independent variable and response status as the dependent variable. We chose the DC pair with highest sum of the coefficient of determination *R*^2^ (linear regression) and highest mean accuracy (logistic regression). We calculated Spectra cell score trends over the DCs by fitting a generalized additive model as implemented in Palantir’s _gam_fit_predict and calculate_gene_trends functions^106^ using cell scores instead of gene expression and DCs instead of pseudotime (Fig. 5a):

$${y}_{i,\,j,k}={\beta }_{0}+f(D{C}_{i,k})$$

where *y*_*i*,*j*,*k*_ is the cell score of cell *i*, factor *j* and the *k*th DC, and *DC*_*i*,*k*_ is the *k*th DC for cell *i*. We then visualized cell score trends using the plot_gene_trend_heatmap function from Palantir.

To calculate compositional changes during ICT, we used the Milo package^56^. Milo is analogous to differential gene expression analysis, but instead of identifying genes that differ between two groups of cells, it tests for differential cell density in (possibly overlapping) neighborhoods of a *k*-nearest neighbors (KNN) cell–cell similarity graph across different conditions. We chose the default fraction of 0.1 to be sampled as index cells from the KNN graph, such that representative cellular neighborhoods were only constructed for those index cells. Milo counts the number of cells per sample in each neighborhood and uses a generalized linear model with a negative binomial distribution to test for differences in abundance. Milo also accounts for multiple comparison testing by computing a spatial FDR.

For the Bassez dataset, we constructed a KNN graph on macrophages and monocytic cells. The Milo paper gives the following heuristic to estimate an optimal *k* parameter^56^:

$$k\ge S* a$$

where *S* is the number of samples (here, 79), and *a* is an arbitrary scaling parameter. Following the authors’ suggestion of 3 ≤ *a* ≥ 5 resulted in an overly large *k* parameter 237 ≤ *k* ≥ 395. We therefore chose *k* to be smaller than the smallest population of cells identified by clustering (58 cells) but close to the *k* parameter obtained by the heuristic above, resulting in *k* = 50 to construct the KNN graph and to identify the nearest 50 neighbors of the index cells.

We then assessed the fold change of cell states under PD-1 blockade using the following regression formula:

$${y}_{ns} \sim response+timepoint+timepoint* response$$

where *y* is the number of cells from sample *s* in neighborhood *n*, and *response* status is defined as 0 for non-responders and 1 for responders. We defined the *timepoint* as 0 for before pretherapy and 1 for on-therapy. The notation *timepoint* * *response* indicates the interaction between the *timepoint* and *response* variables. We then identified the neighborhoods specifically enriched for non-responders under therapy by taking the subset of neighborhoods based on the estimated regression coefficients. First, we identified the neighborhoods specifically enriched under therapy by retaining a subset of all neighborhoods with an FDR of <0.05 and coefficient (log (fold change)) of >0 for the *timepoint* parameter for further analysis. From these, we took a subset of the neighborhoods enriched for non-responders compared to responders under therapy by selecting neighborhoods with an interaction FDR of <0.05 and an interaction coefficient (log (fold change)) of <0 for further analysis. We then compared the mean factor cell scores for these neighborhoods to all remaining neighborhoods.

The Zhang dataset contained fewer individuals treated with immunotherapy than the Bassez dataset (*n* = 8 versus *n* = 42) and therefore did not allow for testing as many covariates. We thus chose a slightly different strategy to find macrophage neighborhoods enriched for non-responders under therapy. For the Bassez dataset, we took a subset of 16 clusters (11,466 cells) annotated as mature macrophages (12 clusters and 9,385 cells) or more immature myeloid-derived (suppressor) cells/monocytic cells (4 clusters and 2,081 cells; Supplementary Table 2) and selected samples from individuals classified as non-responders treated with anti-PD-L1 (see Zhang dataset) for a total of 4,318 cells and five samples. Analogous to the *k* parameter selection strategy above, we constructed a KNN graph using a *k* parameter of 20, which was smaller than the smallest cell population detected by clustering (22 cells). We then defined Milo neighborhoods as the 30 nearest neighbors of the index cells. We fitted the Milo model using the following regression formula:

$${y}_{ns} \sim timepoint$$

where *y* is the number of cells from sample *s* in neighborhood *n*, and *timepoint* is defined as either 0 for pretherapy or 1 for on-therapy. We took a subset of the neighborhoods with an FDR of <0.2 and a coefficient (log (fold change)) of >0 for the *timepoint* parameter for further analysis. As for the Bassez dataset, we then compared the factor cell scores for this group with all remaining neighborhoods.

### Statistical analysis and visualization

*P* values were calculated as indicated above using the Milo, scipy and statsmodels Python packages. No normality assumption was made. We used a Mann–Whitney *U*-test for independent samples and a Wilcoxon matched-pairs signed-rank test for paired samples. If not indicated differently, all *P* values are two-sided and corrected for multiple comparisons (Bejamini–Hochberg method). A *P* value of 0.05 was considered statistically significant. Cohen’s *d* was calculated according to the following formula:

$$d=\left\vert \frac{{\mathrm{mean}}(a)-{\mathrm{mean}}(b)}{s}\right\vert$$

where *s* is the pooled standard deviation, and mean(*a*) and mean(*b*) are the means of groups *a* and *b*, respectively.

Data were visualized using the matplotlib and seaborn Python packages and GraphPad Prism v9 for Microsoft Windows and were edited in Adobe Illustrator Creative Cloud (v27.0). Panels from Figs. 3c,f and 4d and Extended Data Fig. 3c are duplicated in Extended Data figures to enable side-by-side visual comparisons. The following software packages were used: absl-py (1.0.0), anndata (0.8.0), arpack (3.7.0), cython (0.29.28), fsclvm (1.0.0.dev10), h5py (3.6.0), igraph (0.10.1), intervaltree (2.1.0), jsonpickle (2.1.0), jsonschema (3.2.0), jupyter (1.0.0), leidenalg (0.8.8), matplotlib (3.5.0), networkx (2.6.3), numba (0.54.1), numpy (1.20.3), opt_einsum (3.3.0), pandas (1.3.5), pip (22.1.2), Python (3.7.6), python-igraph (0.10.1), pytorch (1.10.1), pyvis (0.1.9), scanpy (1.8.2), schpf (0.5.0), scikit-learn (1.0.2), scipy (1.7.3), seaborn (0.11.2), Slalom (1.0.0.dev11), Spectra (0.1.0), statsmodels (0.12.2), tqdm (4.62.3), umap-learn (0.5.2), zifa (0.1), anndata (0.8.0), cellrank (1.5.1), DoubletDetection (2.5.2), graphviz (2.50.0), joypy (0.2.6), jupyter (1.0.0), kneed (0.7.0), leidenalg (0.8.10), notebook (6.4.12), numpy (1.21.6), numpy_groupies (0.9.17), palantir (1.0.1), pandas (1.4.2), phenograph (1.5.7), pickleshare (0.7.5), plotly (5.10.0), pygam (0.8.0), pygments (2.12.0), pygraphviz (1.9), python-utils (3.3.3), pyvis (0.3.0), r-base (4.1.3), rpy2 (3.5.1), scanpy (1.9.1), scikit-learn (1.1.1), scipy (1.8.1), seaborn (0.11.2), miloR (3.16), pytorch (1.7.0), matplotlib (3.5.1), cudatoolkit (10.2.89), h5py (3.3.0), hdf5 (3.3.0), igraph (0.9.4), networkx (2.5.1) and numba (0.51.1).

### Reporting summary

Further information on research design is available in the Nature Portfolio Reporting Summary linked to this article.

## Online content

Any methods, additional references, Nature Portfolio reporting summaries, source data, extended data, supplementary information, acknowledgements, peer review information; details of author contributions and competing interests; and statements of data and code availability are available at 10.1038/s41587-023-01940-3.

### Supplementary information

Supplementary Information Supplementary Figs. 1–7 and Note.

Reporting Summary

Supplementary Tables Supplementary Table 1 Gene sets comprising the human immunology knowledge base. Supplementary Table 2 Cell-type annotations. Supplementary Table 3 Tumor reactivity factor marker genes (related to Fig. 3). Supplementary Table 4 Spectra factors are correlated with DCs (related to Fig. 5). Supplementary Table 5 Relevant metadata for individuals in each study. Supplementary Table 6 Marker genes for immune lineages.

### Source data

Source Data Fig. 1 Statistical source data.

Source Data Fig. 2 Statistical source data.

Source Data Fig. 3 Statistical source data.

Source Data Fig. 4 Statistical source data.

Source Data Fig. 5 Statistical source data.

Source Data Fig. 6 Statistical source data.

Source Data Extended Data Fig. 1 Statistical source data.

Source Data Extended Data Fig. 2 Statistical source data.

Source Data Extended Data Fig. 3 Statistical source data.

Source Data Extended Data Fig. 4 Statistical source data.

Source Data Extended Data Fig. 5 Statistical source data.

Source Data Extended Data Fig. 6 Statistical source data.

Source Data Extended Data Fig. 7 Statistical source data.

Source Data Extended Data Fig. 8 Statistical source data.

## Data Availability

Count matrices for the PBMC^8^ and Zhang datasets^23^ were obtained from GEO (https://www.ncbi.nlm.nih.gov/geo/) using accession numbers GSE178431 and GSE169246, respectively. Count matrices of the Bassez^10^ and Caushi^30^ datasets were kindly provided by the authors and are also available at http://biokey.lambrechtslab.org and GEO (GSE173351), respectively. Raw read counts for the Bassez^10^ dataset are available in the EGA (EGAS00001004809 and EGAD00001006608). Count matrices for the Salcher atlas^71^ were obtained from Zenodo (10.5281/zenodo.7227571). Gene sets from the Cytopus knowledge base are available on GitHub and Zenodo (https://github.com/wallet-maker/cytopus and 10.5281/zenodo.7306238). Source data are provided with this paper.

## Code availability

Spectra is available as an open-source Python package at https://github.com/dpeerlab/spectra/, and the immunology knowledge base is available at https://github.com/wallet-maker/cytopus/. Notebooks to reproduce figures are available at https://github.com/dpeerlab/SpectraReproducibility.
