# Supplementary material for: Supervised discovery of interpretable gene programs from single-cell data
Source: Nat Biotechnol. 2023 Sep 21;42(7):1084–95. doi: 10.1038/s41587-023-01940-3 (PMC10958532; doi:10.1038/s41587-023-01940-3)
Supplement: Supplementary file 1 — Supplementary Figs. 1–7 and Note. [file 41587_2023_1940_MOESM1_ESM.pdf]

nature biotechnology

Article

https://doi.org/10.1038/s41587-023-01940-3

Supervised discovery of interpretable gene programs from single-cell data

In the format provided by the authors and unedited

Supervised discovery of interpretable gene programs from single-cell data - Supplementary Information

Russell Z. Kunes*[1,2], Thomas Walle*[1,3,4,5], Max Land[1], Tal Nawy[1], Dana Pe'er[1,6]

[1] Computational and Systems Biology Program, Sloan Kettering Institute, Memorial Sloan Kettering Cancer Center; New York, NY, USA

[2] Department of Statistics, Columbia University, New York, NY, USA

[3] Clinical Cooperation Unit Virotherapy, German Cancer Research Center (DKFZ), Heidelberg, Germany

[4] Department of Medical Oncology, National Center for Tumor Diseases, Heidelberg University Hospital, Heidelberg, Germany

[5] German Cancer Consortium (DKTK), Heidelberg, Germany

[6] Howard Hughes Medical Institute; Chevy Chase, MD, USA

*equal contribution

#correspondence: peerd@mskcc.org

Supplementary Figures

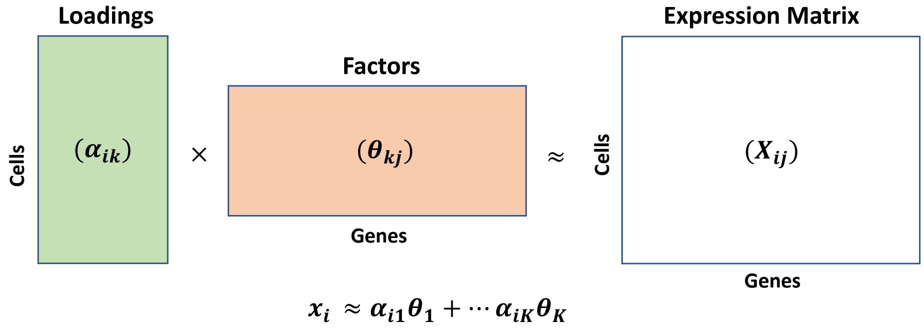

Supplementary Fig. 1 | Matrix decomposition methods applied to single cell RNA sequencing data decompose the expression matrix as a product of cell level loadings (α_i) and latent factors (θ_k)

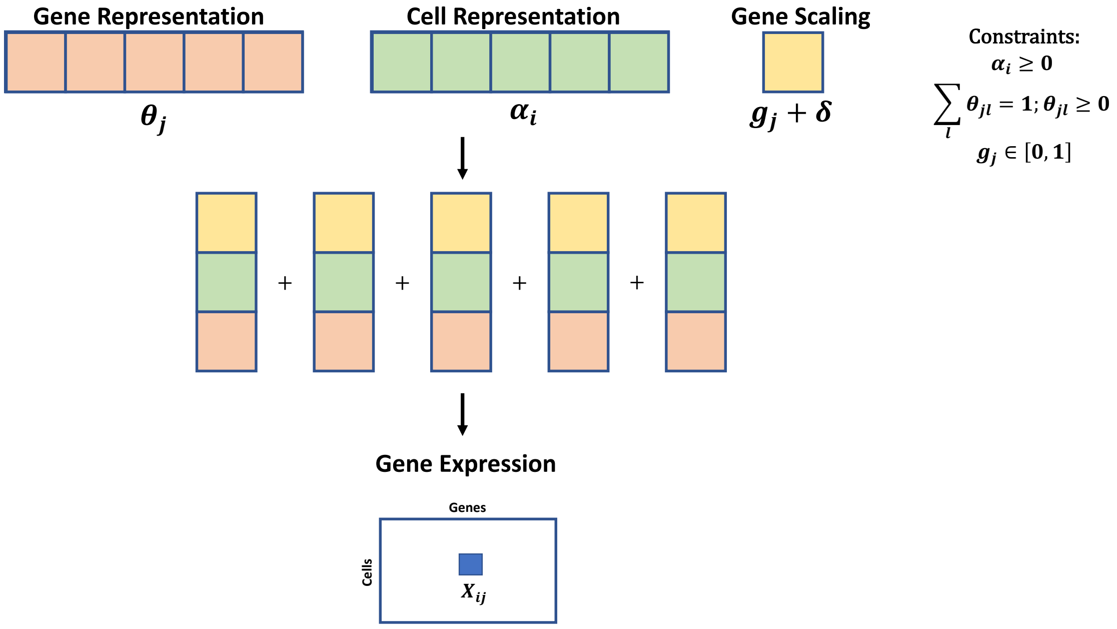

Supplementary Fig. 2 | Backbone of the Spectra expression model. Each element of the expression matrix $\mathbf{X}_{i,j}$ is modeled as an inner product of gene representations (θ_j) and cell representations (α_i), weighted by gene scalings $g_j + \delta$

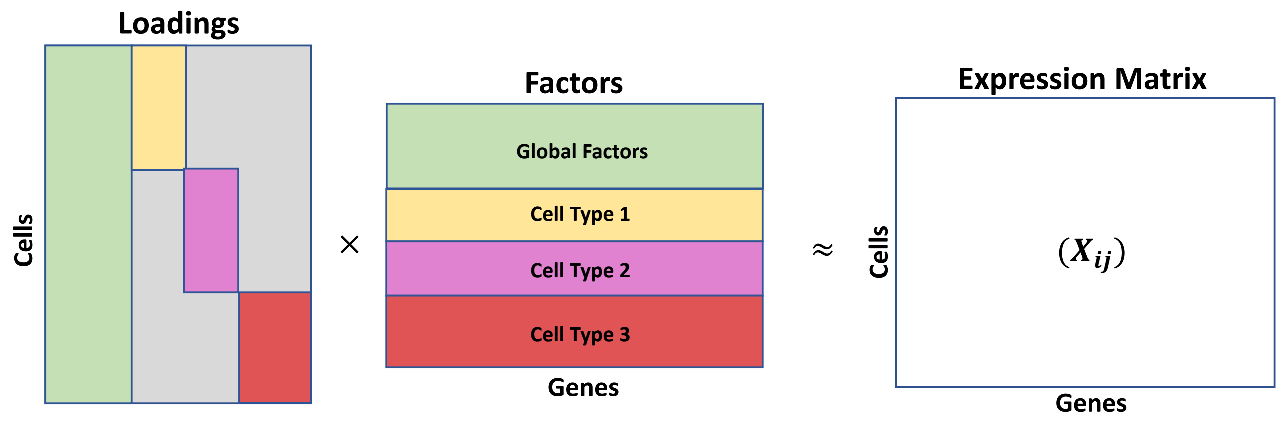

Supplementary Fig. 3 | A matrix decomposition method that fits cell type specific and global factors. Green colored global factors correspond to green colored loadings which are potentially non-zero for all cells, while the three cell type specific factors have sparsity pattern in their loadings corresponding to cell type identity. The model still decomposes the expression count matrix as a product of cell level loadings (α_i) and latent factors (θ_k)

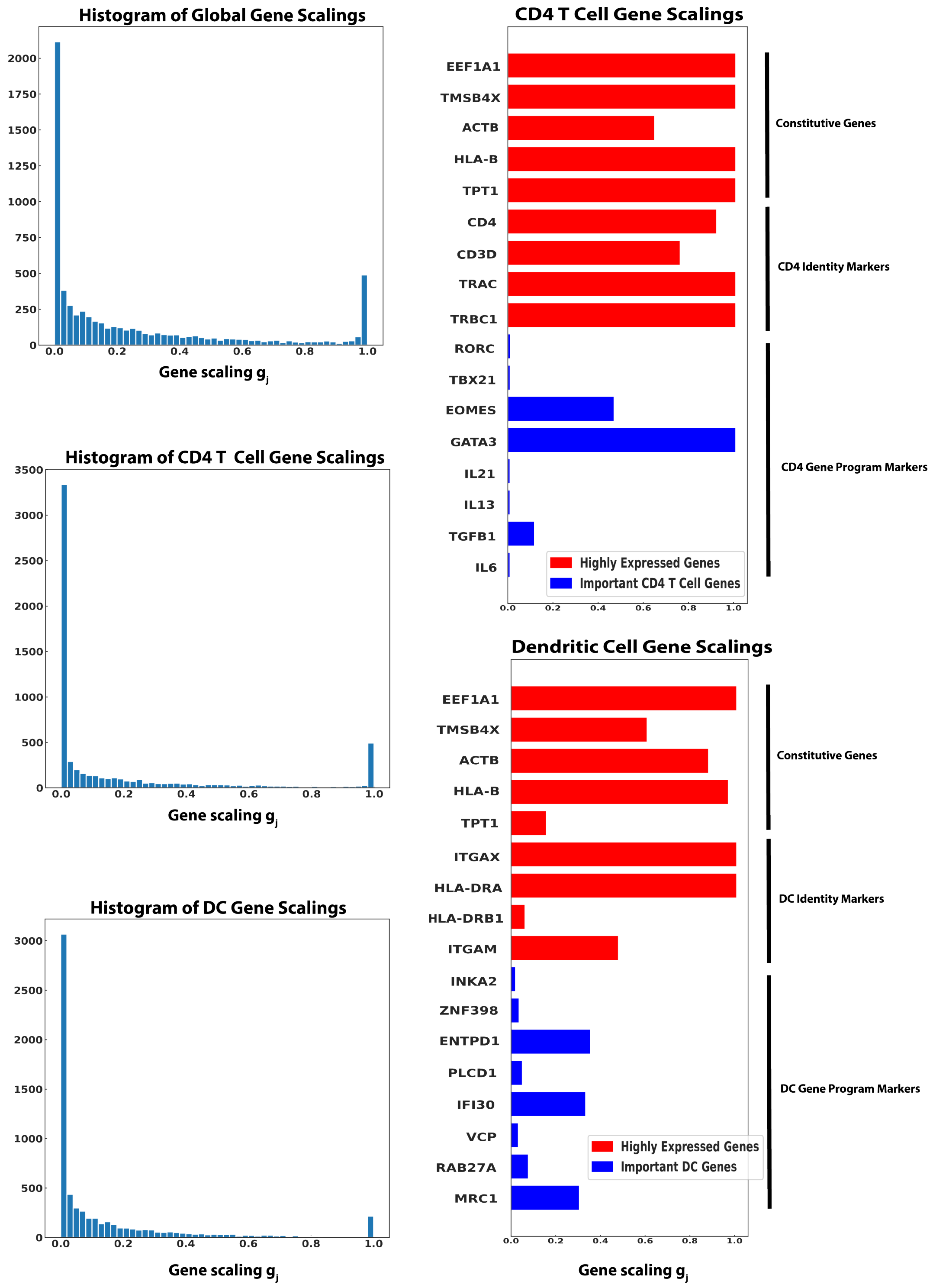

Supplementary Fig. 4 | *Left*: Distribution of gene scale factors for global (top), CD4 T cells (middle), and dendritic cells (bottom) on Bassez data ($\lambda = 0.01$, $\delta = 0.001$). Mode at $1 + \delta$ is due to constraint on maximum ratio of scale factors. *Right*: Gene scale values for specific genes for CD4 T cells (top) and dendritic cells (bottom) including constitutive genes, identity markers, and genes involved in cell type specific gene programs

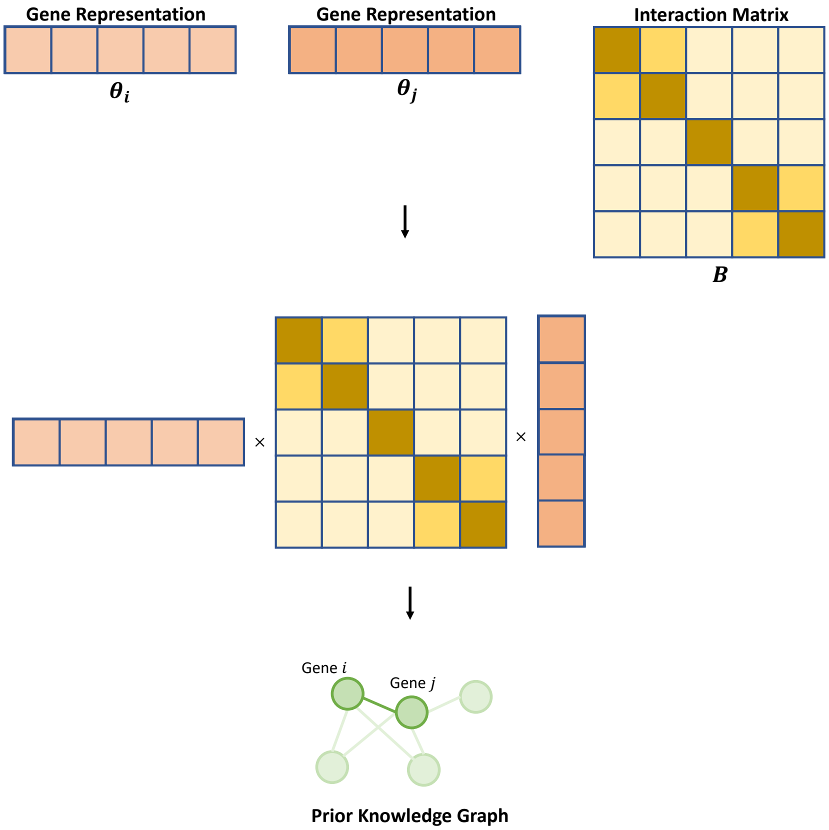

Supplementary Fig. 5 | Backbone of the Spectra graph model. Spectra incorporates prior information by encouraging gene representations to be able to predict a set of annotated gene pairs, which can be represented by a graph. Given gene representations, θ_i and θ_j, an edge is predicted by a weighted inner product, weighted by interaction matrix B, $\langle \theta_i, B\theta_j \rangle$

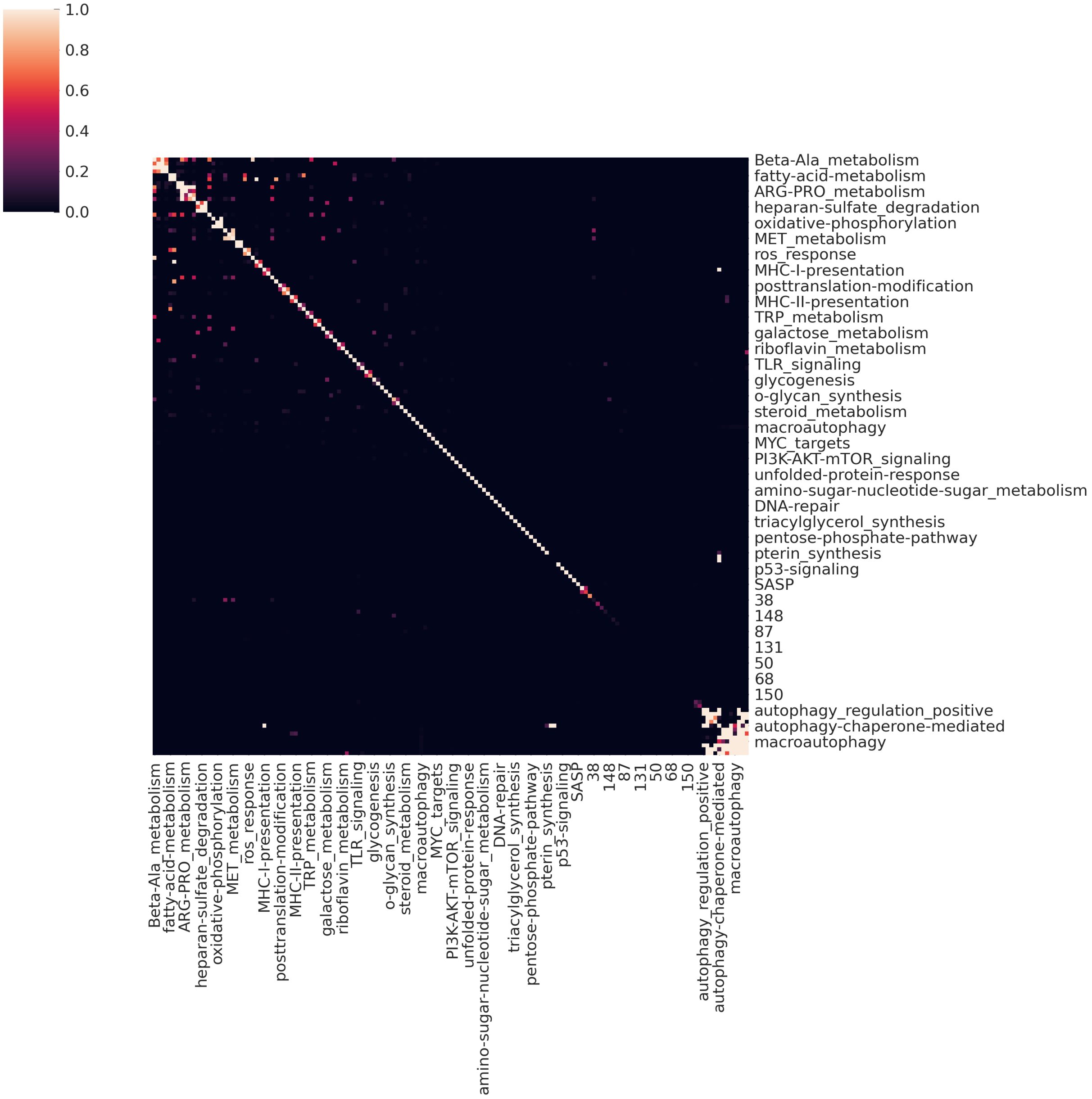

Supplementary Fig. 6 | Global factor interaction matrix (B) estimated from the Bassez dataset. Gene programs with 0 values on the diagonal of the matrix are novel.

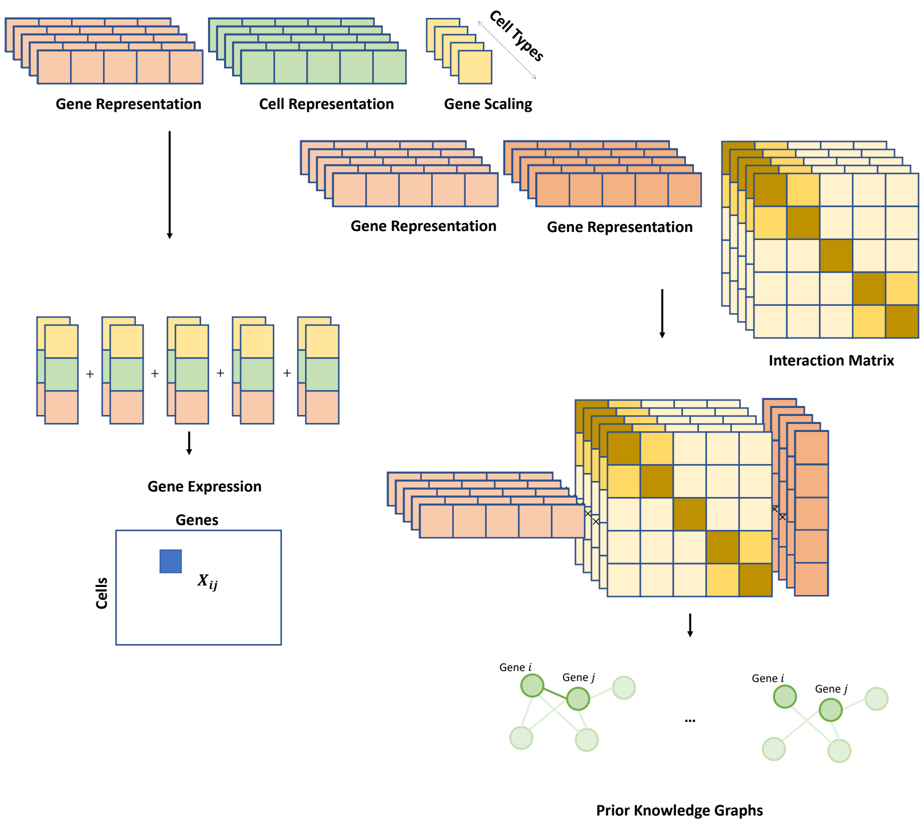

Supplementary Fig. 7 | Spectra model. The full Spectra model combines the components described in Methods Section 'Modeling gene expression as a low rank product' through Section 'The factor interaction matrix tunes the weight of the gene-gene prior'. In addition to it's global representation, each gene is also equipped with a per cell type representation. Each cell is also equipped with an additional per cell type representation that is non-zero only for the cells assigned cell type. The individual per cell type arms are aggregated into a single gene expression estimate for each cell and gene. For each cell type that has a prior information graph, a weighted inner product weighted by cell type specific factor interaction matrices estimates the edge probability for each pair of genes.

Supplementary Note

Spectra behaves like gene set averaging when $\lambda \to 0$

A standard real analysis argument shows this formally. For ease of exposition we assume that the gene scalings g are 1 and $\rho = \kappa = 0$. Simulations confirm the result. Recall that $B_{kl} \in [0,1]$ and $\theta_i \in \Delta^{K-1}$. We consider the result as $\tilde{\lambda} := 1/\lambda$ goes to ∞.

Theorem 1 *Suppose that $\mathcal{G}_1, \ldots, \mathcal{G}_K$ are a partition of $\{1, \ldots, p\}$ and A is constructed such that $A_{ij} = 1$ if $i \neq j \in \mathcal{G}_k$ for some k. Define $\mathcal{L}_1(\alpha, \theta) = \sum_{i=1}^{n} \sum_{j=1}^{p} \mathbf{X}_{ij} \log(\alpha_i^\top \theta_j) - \alpha_i^\top \theta_j$ and $\mathcal{L}_2(\theta) = \sup_B \sum_{i=1}^{p} \sum_{j=1, i\neq j}^{p} A_{ij} \log(\theta_i^\top B \theta_j) + (1 - A_{ij}) \log(1 - \theta_i^\top B \theta_j)$. We have $\lim_{\tilde{\lambda}\to\infty}$ argmax$_{\alpha_{ik}, \theta_{jk}} \mathcal{L}_1(\alpha, \theta) + \tilde{\lambda}\mathcal{L}_2(\theta) = \left(\frac{\sum_{j \in \mathcal{G}_k} X_{ij}}{|\mathcal{G}_k|}, \mathbf{1}[j \in \mathcal{G}_k]\right)$ (or some permutation)*

Lemma 1 *If each block is at least size 2, $\mathbf{1}[j \in \mathcal{G}_k]$ is a unique solution up to permutations $\sigma(k)$ for the loss $\mathcal{L}_2(\theta)$. Since θ is constrained to a compact set and $\mathcal{L}_2$ is continuous, if $\sum_{jk} |\theta_{jk} - \mathbf{1}[j \in \mathcal{G}_{\sigma(k)}]| > \epsilon$ we must have a $\delta > 0$ with $\mathcal{L}_2(\theta) < -\delta$*

Proof of lemma. From the strict convexity of the Bernoulli log likelihood we have that the maximum must satisfy $A_{ij} = \theta_i^\top B \theta_j$ for $i \neq j$. Define the following subsets of $\{1, \ldots, K\}$: $\tilde{\mathcal{G}}_k := \bigcup_{i \in \mathcal{G}_k} \{q : \theta_{iq} > 0\}$. Note that each $\tilde{\mathcal{G}}_i$ is nonempty. If one were empty we would have $\theta_i = 0$ for all $i \in \mathcal{G}_k$ which is impossible if each block has size at least 2.

Consider $(p, q) \in \mathcal{G}_i$ with $p \neq q$. By assumption $\theta_p^\top B \theta_q = 1$. Thus it must be the case that we have $B_{kl} = 1$ for some k, l (possibly $k = l$) and $\theta_{pk} > 0$, $\theta_{ql} > 0$. Further $k \notin \tilde{\mathcal{G}}_j$ for all $j \neq i$ and $l \notin \tilde{\mathcal{G}}_j$ for all $j \neq i$. To see this, we have by assumption, $\theta_{pk} > 0$ and $\theta_p^t B \theta_t = 0$ for all $t \notin \mathcal{G}_i$. If some such t had $\theta_{tl} > 0$ then $B_{kl}\theta_{pk}\theta_{tl} = 0$ a contradiction. This shows that k and l are both unique to $\tilde{\mathcal{G}}_i$. But this applies to each $i = 1, \ldots, K$. So $k = l$ and $|\tilde{\mathcal{G}}_i| = 1$ for all i. Then we have $B_{kk}\theta_{pk}\theta_{qk} = 1$ for a unique k and $B_{kk} = \theta_{pk} = \theta_{qk} = 1$.
Remark that $\tilde{\theta}_{jk} := \mathbf{1}[j \in \mathcal{G}_k]$ is a global minimizer of $\mathcal{L}_2(\theta)$. An upper bound of $\mathcal{L}_2(\theta)$ is:

$$\begin{aligned}\mathcal{L}_2(\theta) &\leq \tilde{\lambda} \sum_i \sum_j A_{ij} \log A_{ij} + (1 - A_{ij}) \log(1 - A_{ij}) \\ &= 0\end{aligned}$$

while setting $B = I$ gives $\tilde{\theta}_i B \tilde{\theta}_j = \mathbf{1}(\cup_{k=1}^{K} \{i, j \in \mathcal{G}_k\}) = A_{ij}$ matching the upper bound. $\mathcal{L}_2(\alpha, \theta)$ is convex in either argument and the solution to argmax$_\alpha \mathcal{L}_1(\alpha, \tilde{\theta})$ is given by $\tilde{\alpha}_{ik} := \frac{\sum_{j \in \mathcal{G}_k} X_{ij}}{|\mathcal{G}_k|}$, which is clear by the computation:

$$\begin{aligned}\mathcal{L}(\alpha, \tilde{\theta}) &= \sum_{i=1}^{n} \sum_{j=1}^{p} X_{ij} \sum_{k=1}^{K} \mathbf{1}[j \in \mathcal{G}_k] \log(\alpha_{ik}) - \sum_{k=1}^{K} \mathbf{1}[j \in \mathcal{G}_k] \alpha_{ik} \\ \frac{\partial \mathcal{L}(\alpha, \tilde{\theta})}{\partial \alpha_{ik}} &= \sum_{j=1}^{p} \mathbf{1}[j \in \mathcal{G}_k] \frac{X_{ij}}{\alpha_{ik}} - |\mathcal{G}_k|\end{aligned}$$

Now define $C := \mathcal{L}_1(\tilde{\alpha}, \tilde{\theta})$ and define $\kappa := \sum_{i,j}\big(X_{ij}\log(X_{ij}) - X_{ij}\big) \geq \mathcal{L}_1(\alpha, \theta)$ (with $x\log(x)$ defined as 0). κ is the unconstrained maximizer of the log likelihood function. For a given ϵ we can choose $\lambda_0 > \frac{\kappa - C}{\delta}$ where δ is chosen such that $\mathcal{L}_2(\theta) + \delta < \mathcal{L}_2(\tilde{\theta})$ for all θ such that $||\tilde{\theta} - P_\sigma(\theta)||_F > \epsilon$ for all column permutations P_σ (by the Lemma). For λ_0, if we have:

$$\mathcal{L}_1(\alpha, \theta) + \lambda_0 \mathcal{L}_2(\theta) > C + \lambda_0 \mathcal{L}_2(\tilde{\theta})$$

Then it follows that $||\tilde{\theta} - P_\sigma(\theta)||_F \leq \epsilon$ for some σ, since otherwise we would have $\lambda_0 \leq \frac{\kappa - C}{\delta}$. Further, for the chosen λ_0 the argmax is nonempty as $(\tilde{\theta}, \tilde{\alpha})$ is a maximizer outside of a compact set (it is standard to show that the maximizing α is a continuous function of θ and the set $||\tilde{\theta} - P_\sigma(\theta)||_F \leq \epsilon$ is compact so its image is compact). Since $\text{argmax}_\alpha \mathcal{L}_1(\alpha, \theta)$ is a continuous function ($\mathcal{L}_1$ is smooth and under mild assumptions on X has a unique minimizer for each θ) it follows that for an ϵ_2, ϵ can be chosen small enough so that $||\alpha - \tilde{\alpha}|| \leq \epsilon_2$.

Corollary 1 *For sufficiently large $\tilde{\lambda}$ there is a global optimum of $\mathcal{L}_1 + \tilde{\lambda}\mathcal{L}_2$ which is arbitrarily close to the gene set averaging solution.*

This corollary explains why Spectra converges extremely quickly for large values of $\tilde{\lambda}$.
