# Supplementary material for: Supervised discovery of interpretable gene programs from single-cell data
Source: Nat Biotechnol. 2023 Sep 21;42(7):1084–95. doi: 10.1038/s41587-023-01940-3 (PMC10958532; doi:10.1038/s41587-023-01940-3)
Supplement: Supplementary file 2 — Reporting Summary [file 41587_2023_1940_MOESM2_ESM.pdf]

nature portfolio

Corresponding author(s): Dana Pe'er

Last updated by author(s): Aug 7, 2023

Reporting Summary

Nature Portfolio wishes to improve the reproducibility of the work that we publish. This form provides structure for consistency and transparency in reporting. For further information on Nature Portfolio policies, see our Editorial Policies and the Editorial Policy Checklist.

Statistics

For all statistical analyses, confirm that the following items are present in the figure legend, table legend, main text, or Methods section.

n/a	Confirmed	
☐	☒	The exact sample size (*n*) for each experimental group/condition, given as a discrete number and unit of measurement
☐	☒	A statement on whether measurements were taken from distinct samples or whether the same sample was measured repeatedly
☐	☒	The statistical test(s) used AND whether they are one- or two-sided<br>*Only common tests should be described solely by name; describe more complex techniques in the Methods section.*
☐	☒	A description of all covariates tested
☐	☒	A description of any assumptions or corrections, such as tests of normality and adjustment for multiple comparisons
☐	☒	A full description of the statistical parameters including central tendency (e.g. means) or other basic estimates (e.g. regression coefficient) AND variation (e.g. standard deviation) or associated estimates of uncertainty (e.g. confidence intervals)
☐	☒	For null hypothesis testing, the test statistic (e.g. *F*, *t*, *r*) with confidence intervals, effect sizes, degrees of freedom and *P* value noted<br>*Give P values as exact values whenever suitable.*
☒	☐	For Bayesian analysis, information on the choice of priors and Markov chain Monte Carlo settings
☒	☐	For hierarchical and complex designs, identification of the appropriate level for tests and full reporting of outcomes
☐	☒	Estimates of effect sizes (e.g. Cohen's *d*, Pearson's *r*), indicating how they were calculated

Our web collection on statistics for biologists contains articles on many of the points above.

Software and code

Policy information about availability of computer code

Data collection	No software was used for data collection.
Data analysis	detailed data analysis in Supplementary Methods. Relevant software packages:<br>https://github.com/dpeerlab/spectra<br>https://github.com/wallet-maker/cytopus<br>GraphPad Prism 9 for Windows<br>Adobe Illustrator Creative Cloud v27.0<br>absl-py (1.0.0), anndata (0.8.0), arpack (3.7.0), cython (0.29.28), fsclvm (1.0.0.dev10), h5py (3.6.0), igraph (0.10.1), intervaltree (2.1.0), jsonpickle (2.1.0), jsonschema (3.2.0), jupyter (1.0.0), leidenalg (0.8.8), matplotlib (3.5.0), networkx (2.6.3), numba (0.54.1), numpy (1.20.3), opt_einsum (3.3.0), pandas (1.3.5), pip (22.1.2), python (3.7.6), python-igraph (0.10.1), pytorch (1.10.1), pyvis (0.1.9), scanpy (1.8.2), schpf (0.5.0), scikit-learn (1.0.2), scipy (1.7.3), seaborn (0.11.2), slalom (1.0.0.dev11), spectra (0.1.0), statsmodels (0.12.2), tqdm (4.62.3) umap-learn (0.5.2), zifa (0.1), anndata (0.8.0), cellrank (1.5.1), doubletdetection (2.5.2), graphviz (2.50.0), joypy (0.2.6), jupyter (1.0.0), kneed (0.7.0), leidenalg (0.8.10), notebook (6.4.12), numpy(1.21.6), numpy_groupies (0.9.17), palantir (1.0.1), pandas (1.4.2), phenograph (1.5.7), pickleshare (0.7.5), plotly (5.10.0), pygam (0.8.0), pygments (2.12.0), pygraphviz (1.9), python-utils (3.3.3), pyvis (0.3.0), r-base (4.1.3), rpy2 (3.5.1), scanpy (1.9.1), scikit-learn (1.1.1), scipy (1.8.1), seaborn (0.11.2), miloR (3.16), pytorch (1.7.0), matplotlib (3.5.1), cudatoolkit (10.2.89), h5py (3.3.0), hdf5 (3.3.0), igraph (0.9.4), networkx (2.5.1), numba (0.51.1)

For manuscripts utilizing custom algorithms or software that are central to the research but not yet described in published literature, software must be made available to editors and reviewers. We strongly encourage code deposition in a community repository (e.g. GitHub). See the Nature Portfolio guidelines for submitting code & software for further information.

Data

Policy information about availability of data

All manuscripts must include a data availability statement. This statement should provide the following information, where applicable:

- Accession codes, unique identifiers, or web links for publicly available datasets
- A description of any restrictions on data availability
- For clinical datasets or third party data, please ensure that the statement adheres to our policy

Count matrices for the PBMC and Zhang dataset were obtained from the Gene expression Omnibus (GEO, https://www.ncbi.nlm.nih.gov/geo/) using the following accession number: GSE178431, GSE169246, respectively. Count matrices of the Bassez and Caushi dataset were kindly provided by the authors and are also available at (http://biokey.lambrechtslab.org) and GEO (GSE173351), respectively. Raw read counts for the Bassez data are available in the European Genome-phenome Archive (EGA) (EGAS00001004809, EGAD00001006608). Count matrices for the Salcher atlas were obtained from Zenodo (https://doi.org/10.5281/zenodo.6411867). Gene sets from the cytopus knowledge base are available on github and zenodo (https://github.com/wallet-maker/cytopus, 10.5281/zenodo.7306238).

Human research participants

Policy information about studies involving human research participants and Sex and Gender in Research.

Reporting on sex and gender	One of four PBMC donors is of female sex in the PBMC dataset. All patients in the breast cancer datasets are of female sex. Male breast cancer is rare (<1% of all breast cancers) and this minority was not included. 9 of 16 and 133 of 297 patients with known sex status were of female sex in the Caushi and Salcher lung cancer studies, respectively. Gender identity was not reported.
Population characteristics	Population characteristics are outlined in Supplementary methods. Mean patient age: 61,47,68,60,28 and %female: 100,100,56,41, 25% and Union for International Cancer Control % stage 4: 0,53,0,10,0 for the Bassez, Zhang, Caushi, Salcher and Kartha data respectively.
Recruitment	Retrospective cohort study (Kartha et al, Caushi et al.). Prospective recruitment registered trial (Bassez et al.), prospective chart review unregistered trial (Zhang et al.). Meta-analysis (Salcher et. al). Full study details in the original publications: 10.1016/j.xgen.2022.100166, 10.1038/s41586-021-03752-4, 10.1016/j.ccell.2021.09.010 , 10.1038/s41591-021-01323-8 , 10.1016/j.ccell.2022.10.008
Ethics oversight	This manuscript includes multiple datasets approved by ethics oversight at the original authors' institutions:<br>local medical ethics committee of the University Hospitals Leuven (S60100),<br>Institutional Review Boards (IRB) at Johns Hopkins University (JHU) and Memorial Sloan Kettering Cancer Center,<br>Ethical Committee of National Cancer Center/Cancer Hospital, Chinese Academy of Medical Sciences (No. 18-216/1794), and<br>the institutional review board at Medical University Innsbruck, Austria (AN214-0293 342/4.5)

Note that full information on the approval of the study protocol must also be provided in the manuscript.

Field-specific reporting

Please select the one below that is the best fit for your research. If you are not sure, read the appropriate sections before making your selection.

☒ Life sciences ☐ Behavioural & social sciences ☐ Ecological, evolutionary & environmental sciences

For a reference copy of the document with all sections, see nature.com/documents/nr-reporting-summary-flat.pdf

Life sciences study design

All studies must disclose on these points even when the disclosure is negative.

Sample size	No sample size calculation was performed for this study. Sample size estimation for original studies can be retrieved from the original publications. 10.1016/j.xgen.2022.100166, 10.1038/s41586-021-03752-4, 10.1016/j.ccell.2021.09.010 , 10.1038/s41591-021-01323-8 , 10.1016/j.ccell.2022.10.008
Data exclusions	No data were excluded. In addition to the low quality cells removed by the original study authors we removed cells with low library size, low library complexity and high mitochondrial gene content as outlined in Methods under "Immunooncology datasets".
Replication	The manuscript validates the tumor reactivity, lysine metabolism and macrophage invasion factors on 3 to 4 datasets (including one atlas, all with independently fit Spectra models) for a total of >2,000,000 cells, 395 patients and 23 studies.
Randomization	No randomization was performed. This study is to explore biological aspects which do not require randomization or cannot be randomized such as time course comparisons within a patient (Figure 3,5) or associations with biological or behavioral variables such as cancer driver mutations or smoking (Figure 6). While time-course data is matched within a patient and does not required controlling covariates, datasets for

	which relevant biological or behavioral variables were available were too small to regress out covariates (only for 13 EGFR mutated patients both age and sex were available).
Blinding	The study is exploratory in nature therefore no blinding was performed.

Reporting for specific materials, systems and methods

We require information from authors about some types of materials, experimental systems and methods used in many studies. Here, indicate whether each material, system or method listed is relevant to your study. If you are not sure if a list item applies to your research, read the appropriate section before selecting a response.

Materials & experimental systems

n/a	Involved in the study
☒	☐ Antibodies
☒	☐ Eukaryotic cell lines
☒	☐ Palaeontology and archaeology
☒	☐ Animals and other organisms
☐	☒ Clinical data
☒	☐ Dual use research of concern

Methods

n/a	Involved in the study
☒	☐ ChIP-seq
☒	☐ Flow cytometry
☒	☐ MRI-based neuroimaging

Clinical data

Policy information about clinical studies

All manuscripts should comply with the ICMJE guidelines for publication of clinical research and a completed CONSORT checklist must be included with all submissions.

Clinical trial registration	NCT03197389
Study protocol	Study protocol for the Bassez et al. study can be found in the original publication. 10.1038/s41591-021-01323-8 . For all other studies no study protocols are available.
Data collection	DTHealth TrakCare System at National Cancer Center, Beijing, China (before 12/2021, period unknown). University Hospitals Leuven (01/18-02/20). Johns Hopkins Sidney Kimmel Comprehensive Cancer Center and Memorial Sloan Kettering Cancer Center (2015-2018). PBMC from the Kartha data were purchased from AllCells (before September 2022, period unknown) . Salcher metanalysis from multiple studies (collection dates before 2023, period unknown).
Outcomes	Outcomes measures were prospectively defined (clinicaltrials.gov identifier NCT03197389) for the Bassez study. The primary outcome measure was: PD-1 expression after a single dose of pembrolizumab. We do not know whether the authors have reported this outcome measure anywhere. All other studies were explorative in nature without registration of outcome measures.
